# A Randomized Controlled Trial of Acceptance and Commitment Therapy for Type 2 Diabetes Management: The Moderating Role of Coping Styles

**DOI:** 10.1371/journal.pone.0166599

**Published:** 2016-12-01

**Authors:** Zeinab Shayeghian, Hamidreza Hassanabadi, Maria E. Aguilar-Vafaie, Parisa Amiri, Mohammad Ali Besharat

**Affiliations:** 1 Research Center for Social Determinants of Endocrine Health & Obesity Research Center, Research Institute for Endocrine Sciences, Shahid Beheshti University of Medical Sciences, Tehran, Iran; 2 Department of Psychology, Kharazmi University, Tehran, Iran; 3 Department of Psychology, Tarbiat Modares University, Tehran, Iran; 4 Department of Psychology, University of Tehran, Tehran, Iran; Florida International University Herbert Wertheim College of Medicine, UNITED STATES

## Abstract

**Background and Aim:**

Evidence of the efficacy of existing psychological interventions for self-management in diabetes is limited. The current study aimed at assessing the effects of group-based ACT on self-management of patients with T2DM, considering the moderating role of coping styles.

**Methods:**

One hundred and six patients with type 2 diabetes were randomly assigned either to the education alone (*n* = 53) or to a combination of education and group-based acceptance and commitment therapy (*n* = 53) over a period of 10 sessions. In each group, 50 participants completed a 3 month follow-up assessment.

**Results:**

After 3 months, compared to patients who received education alone, those in the group-based acceptance and commitment therapy condition were more likely to use effective coping strategies, reported better diabetes self-care, and optimum glycated hemoglobin (HbA1C) levels in the target range.

**Conclusions:**

Consideration of the role of coping style for a more accurate evaluation of the effects of acceptance and commitment therapy may be a useful addition to services provided for patients with type 2 diabetes.

## Introduction

Type 2 diabetes mellitus (T2DM) is a multidimensional metabolic disease, characterized by increases in blood sugar or hyperglycemia and is caused by a disorder in insulin secretion or functioning or both [1]. The control of diabetes strongly depends on self-management [2], which demands attention being paid to self-care activities [3]. Although many kinds of psychological interventions to control diabetes have been effective, the benefits of these treatments are short lived [4,5]. Furthermore, previous research studies have been conducted with no clear depiction of the real social and cultural situations and contexts, making it difficult for those working in different healthcare settings to adapt the program to their own situations [6].

Besides the development of different types of interventions, a specific protocol developed especially for patients with diabetes within the acceptance and commitment therapy (ACT) framework has been compared to previous interventions and seems more suitable to the chronic nature of diabetes [3]. In spite of this, ACT for diabetes has not been used for diabetes extensively and the role of moderating factors in ACT therapy, like coping styles, has not been considered. For these reasons, the present study aimed at assessing the effects of group-based ACT on self-management of patients with T2DM, considering the moderating role of coping styles.

Acceptance and commitment therapy is a unique empirically based psychological intervention that uses mindfulness strategies and acceptance along with commitment and behavior change strategies, to increase psychological flexibility [7], and is considered to offer more advantages in comparison to other interventions; for instance, most psychological studies involving the application of cognitive-behavior therapy have concentrated on decreasing, changing or stopping negative thoughts related to diabetes. However, ACT focuses on the acceptance of thoughts that emphasize clarification of values and personal goals [3]. The emphasis of ACT on acceptance seems to be a fruitful avenue in the short-term treatment of diabetes because patients are constantly required to cope with facts and events that are part of the very nature of the disease, therefore increasing the opportunities for behavioral change [8,9,10]. To date no (or very few), studies has been published investigating the moderating role of coping styles on the effects of ACT on diabetes self-management, emphasizing the need for related data [11]

To our knowledge, in spite of the advantages of ACT, in comparison to other psychological interventions, the role of moderator variables in the process of testing the effectiveness of ACT in diabetes control has not been addressed in literature. Successful interventions in diabetes control definitely need to consider moderating factors [4] or factors that have a direct effect on the process of self-management, that eventually lead to blood glucose regulation. Moreover, considering the fact that diabetes is a complex disease and requires life-long self-caring activities [12] it may become a source of stress [13], which in turn, by having a detrimental effect on self-caring activities [14], can increase glycated hemoglobin in patients with diabetes [15]. Low psychological flexibility is associated with low self-care activity and increase patient distress [16]; However coping style can moderate these associations. For example, an ineffective coping strategy is associated with increases in stress, lack of regimen compliance, and weak glycemic control [17], all of which may interfere with the treatment of diabetes.

The stress-buffering model offers one mechanism that can be considered in the investigation of the effects of coping styles on glycemic control, which assumes a moderating role of certain coping styles against stress [18], such that patients who successfully cope with stressful events, are more inclined to comply with the treatment regimen in times of stress, and become less vulnerable to the detrimental effects of stress by reducing the likelihood that stress will lead to poor health [19]. Avoidance coping strategies (negative emotion-focused) are associated with negative psychological outcomes, poor treatment adherence and poor metabolic control, whereas, acceptance coping strategies (problem-focused and positive emotion-focused) are associated with better metabolic control [20]. Therefore, it is expected that coping strategies of patients with diabetes undergoing ACT could play a moderating role in the control of blood sugar.

Taking appropriate steps, a priority in the treatment and control of diabetes in order to prevent the devastating side effects of the disease, depends on the investigation of causal mechanisms of moderator variables in psychotherapy and the randomized controlled clinical trials as one of most powerful tools of intervention research [21] employed in this study. Although moderator analysis for treatment effects has been strongly advocated, there has been little formal emphasis on such analyses in randomized controlled trials (RCTs)[22]. Our objective was to assess the moderating role of coping styles in the relationship between group-based acceptance and commitment therapy and self-management of patients with T2DM. For this purpose, patients with T2DM participated in a 10-session group-based ACT and were compared with a group of patients with T2DM who did not attend ACT, within an RCT design. It was hypothesized that: 1) Patients who participated in a group-based ACT intervention and education will show greater improvement of self-management than those patients who received education alone, and this effect will still be observed after a 3- month follow-up. 2) ACT will be more effective in participants who used effective coping strategies than those who used ineffective coping strategies.

## Materials and Methods

### Participants

Patients with T2DM were recruited based on a convenience sampling procedure from the endocrine clinic of the Endocrine Department of Labbafinejad Hospital in Tehran (Iran) between February 2013 and January 2014. Inclusion criteria were age 40 to 60 years, T2DM diagnosed within 1–10 years and no change in diabetes medication for at least 3 months before entering the study. Exclusion criteria were hospitalization, diabetes complications e.g. nephropathy or neuropathy and emergency surgery. Based on these criteria, 133 eligible patients with T2DM were referred to researchers by the endocrinologist from the endocrine clinic, of whom, 75.19% (100 patients) completed the 3-month follow up, as be explained in the Procedure Section. Demographics and Clinical Characteristics of patients are shown in Table 1.

**Table 1:** Demographic and clinical characteristics of patients with type 2 diabetes (N = 100).

Characteristics	Groups			
	ACT (*n* = 50)	Control (*n* = 50)	Overall	*p*
Age in years, *M (SD)*	55.18 (8.26)	55.70 (8.98)	55.44 (8.44)	.76
Gender: *n* (%) female	33 (66)	27 (54)	60 (60)	.31
Education, *n* (%)				
High school and lower	29 (58)	25 (50)	54 (54)	
Diploma and Associate	19 (38)	19 (38)	38 (38)	.32
Bachelor and upper	2 (4)	6 (12)	8 (8)	
Marital status, *n* (%)				
Single	2 (4)	0 (4)	2 (2)	
Married	43 (86)	40 (80)	83 (83)	.15
Widowed	5 (10)	10 (20)	15 (83)	
Diabetes duration in years, *M (SD)*	4.90±1.40	4.54±1.54	4.22±1.49	.70
Body mass index in k/m^2^, *M (SD)*	29.24±4.56	29.45±4.76	29.46±4.62	.82
Hypertension in mmHg, *M (SD)*	13.25±2.01	13.66±1.93	13.46±1.96	.30
Insulin, *n* (%)	1 (2)	1 (2)	2 (2)	
Oral medication, *n* (%)	36 (72)	37 (74)	73 (73)	.97
Insulin + Oral medication, *n* (%)	13 (26)	12 (24)	25 (25)	

M±SD and number of subjects (%); ACT = Acceptance and Commitment Therapy; differences between ACT and control.

### Procedure

The design of the study was a pre-test, post-test and follow-up control-group randomized trial with a 50:50 allocation ratio between the intervention and the comparison groups. A randomized controlled trial was conducted in the Endocrine Department of Labbafinejad Hospital (in the city of Tehran, Islamic Republic of Iran) between February 2013 and January 2014. Before data collection, the study was approved by Ethics Committee of the Research Institute for Endocrine Sciences, Shahid Beheshti University of Medical Sciences. The authors confirm that all ongoing and related trials for this intervention are registered in the Iranian Registry of Clinical Trials at http://www.irct.ir with the following identification: IRCT2012091710860N1.

**Fig 1:**
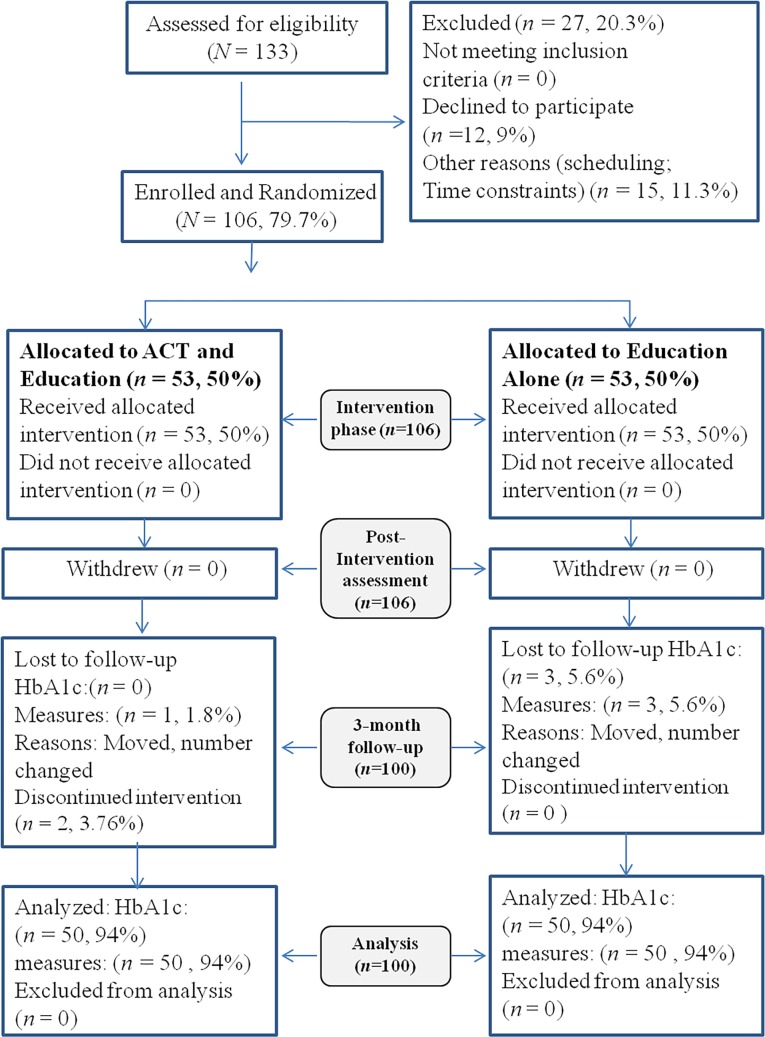
Attrition number for acceptance and commitment therapy and one-day workshop. ACT- Acceptance and Commitment Therapy; HbA1c- glycated hemoglobin.

One hundred thirty-three patients with T2DM were referred to researchers by the endocrinologist from the endocrine department of Labbafinejad hospital to participate in a 1-day educational workshop for self-management of diabetes. Among the 133 referred patients, 27 (20.3%) were excluded for the following reasons: Twelve participants (9%) declined to continue in the study due to limited time of participation or hard access to research location, eleven participants (8.27%) were omitted because they had incomplete questionnaires due to lack of sufficient time to respond or became sick; four patients (3%) were omitted because they were unpunctual and did not keep their appointments set for participation. The remaining 106 patients (79.7%) were randomly assigned (using the random number table) to one of two groups (*n* = 53 each). All patients provided written informed consent. Blood test assessments were provided for free as an incentive for participation. Patients were assessed prior to intervention (ACT or education) immediately following the 10-week intervention period, and at 3-month post-intervention. For the 3-month follow up period, of 106 participants, three patients (5.6%) in the control group, missed appointments and did not respond to telephone calls, and they could not hence complete the self-report measures or the HbA1c test. Also, three patients (5.6%) in the experimental group dropped out of treatment; one participant, had to take care of a patient at home and two other participants changed residence location. Eventually, the analysis was performed for 100 patients (75.19% of 133 referred patients; see Fig 1). Participants were randomly allocated in 2 groups, the ACT group and the control group.

### Intervention

The protocol for this trial and supporting CONSORT checklist are available as supporting information; see S1 Checklist, S1 CONSORT, S1 Protocol and S2 Protocol.

#### Treatment protocols

ACT and education. Participants randomly assigned to ACT (n = 53) attended a workshop, based on an ACT manual (Gregg, 2004; available at http://www.sjsu.edu/people/jennifer.gregg/). Eventually data of only 50 individuals in each group after initial sample (n = 53) was analyzed as 3 dropped out for various reasons. This study provided data at the 3-month follow-up appointment. The protocol used during the training program was based on the structure and format of the 10-session protocol contained in The ACT for diabetes, “The Acceptance and Commitment Therapy for Diabetes Self-Management”, which utilizes mindfulness meditation to enhance an individual’s ability to respond effectively to difficult thoughts and feelings across a variety of problems; this protocol includes exposure-based and experiential exercises, metaphorical uses of language, and methods such as mindfulness training, and it appears that the ACT model leads to effective treatment for diabetes [3].

#### One-day workshop

The control group (n = 53) randomly assigned to education with routine treatment, participated in a public workshop on diabetes control and they remained in a waiting list. In a 2-hour session the workshop covers information regarding the diabetes disease process, blood glucose monitoring, importance of nutrition and physical activity and ways of the prevention of complications.

### Measures

All participants were asked to complete a questionnaire on socio-demographic characteristics including gender, age, marital status, educational level and diabetes duration; weight and height were measured. Prior to beginning the trial, participants completed the following questionnaires: The Brief COPE, the Summary of Diabetes Self-Care Activities (SDSCA) and the Acceptance and Action Diabetes Question (AADQ). The SDSCA and AADQ were re-completed at post-treatment and at three month post-treatment (follow-up), while blood samples were obtained at the 3 assessment points (pretest, posttest and follow-up) and were provided for free as an incentive for participation. The primary medical outcome variable was HbA1C, which is commonly used in diabetes research. A change in the SDSCA was considered the primary outcome measure, while a change in the AADQ was the secondary outcome measure. The brief COPE questionnaire as a moderator helps explain individual differences in the effect of treatment and was completed at pretest.

#### Glycaemic hemoglobin

Glycated hemoglobin (HbA1C) is a form of hemoglobin that is measured to identify the average plasma glucose concentration over the previous 2 to 3 months in the form of a percentage value [3]. The normal range of HbA_1c_ in healthy individuals ranges between 4 to 6% (20–42 mmol), and any reading < 6.5 (< 48 mmol) is considered a good index of diabetes control, based on the American Diabetes Association [23].

#### Summary of diabetes self-care activities

Diabetes self-management was measured using the Summary of Diabetes Self-Care Activities (SDSCA) scale that assesses the seven aspects of the diabetes regimen: Blood-glucose testing, exercise, diet, medications, cigarette smoking, and foot care; response options range from 0 to 7, corresponding to the number of days in a week [24]. In the present study, the alpha Cronbach internal consistency coefficients for total SDSCA was 0.92 and for SDSCA subscales, it ranged from .84 to .94.

#### The acceptance and action diabetes question (AADQ)

Changes in ACT processes were measured by the Acceptance and Action Diabetes Questionnaire (AADQ). This 11-item Likert-type scale measures acceptance of diabetes-related thoughts and feelings and the degree to which they interfere with valued action. The AADQ has good psychometric properties in T2DM patients (i.e. Cronbach’s α = .94 [3].

#### The brief COPE questionnaire

This 28-item short version self-report scale comprised 14 coping strategies [25]. Items are rated on a 1–4 Likert-type scale [26]. Validity and reliability of this measure is adequate [27]. Based on research performed Western nations [18] and Iran [28], all the COPE subscales were grouped in two coping style strategies, labeled effective and ineffective coping styles. Active coping, Acceptance, Positive reframing, Planning, Religion, Humor, Use of emotional support and Use of instrumental support formed the category of effective coping style and Denial, Self-distraction, Substance use, Behavioral disengagement, Venting and Self-blame, the ineffective coping style. Reliability coefficient of internal consistency for the two subscales measuring effective coping (0.91) and ineffective coping (0.89) were good.

### Analytic strategy

Based on a meta-analysis by Brown [8] of diabetes interventions, the required sample size for the current study was calculated as 34 individuals per group, based on a power of 80% and a 2-tailed α of 0.05, considering HbA1C as the primary outcome. However, assuming a 10% loss to follow-up rate, the final sample size was estimated to be 45 subjects per group. The effect size for intervention and control group differences has been considered as 0.65 [8]. The autocorrelation between three time measures was considered as 0.5. The PASS11 (Power Analysis and Sample Size) software and repeated measures ANOVA design (group + time + group × time) have been conducted to calculate sample size. IBM SPSS Statistics 20 was used for the statistical analyses. In all analyses, p-values <0.05 were considered statistically significant. A pre-test evaluation was conducted for the control and experimental groups in terms of glycated hemoglobin, coping style, self-care activities score and acceptance. In order to assess the effects of the ACT intervention on self-management and the stability of the therapy, three repeated measures analysis of variance with post-test and follow-up scores were computed, controlling for pre-test effects (Table 2). This approach is recommended as a robust and reliable statistical strategy for analyzing the results of RCTs [29]. Thereafter, in order to evaluate the moderating role of coping styles (Effective, Ineffective, and Combined) in the relationship between group ACT with glycated hemoglobin, self-care activities and acceptance, three separate two-way analyses of variance were computed (Table 3). For determining the moderating effect of coping styles the computation of a simultaneous test procedure (STP) is recommended [30].

**Table 2:** Results of Repeated Measure ANOVA for effect of the ACT on HbA1c, self-care and acceptance.

			Pretreatment		Post-treatment		Follow-up		Repeated measure ANOVA	
Variables			*M (SD)*		*M (SD)*		*M (SD)*		*F*_(1,97)_	η^2^
	Group	ACT	7.46 (1.66)		7.10 (1.56)		7.03 (1.52)		32.36**	.25
		Control	7.61 (1.38)		7.66 (1.57)		7.81 (1.56)			
HbA1c	Time								5.64*	.05
	Time × HbA1c								4.66*	.05
	Time × Group								16.37**	.22
	Group	ACT	65.34 (18.11)		72.32 (24.98)		73.60 (27.32)		26.74**	.22
		Control	71.56 (17.96)		72.58 (18.84)		73.16 (20.40)			
Self-care	Time								2.65	.03
	Time × Self-care								4.17*	.04
	Time × Group								0.74	.01
	Group	ACT	52.18 (16.14)		61.10 (14.01)		62.56 (15.17)		76.75**	.44
		Control	53.94 (15.57)		53.40 (15.77)		54.08 (17.0)			
Acceptance and action diabetes	Time								2.57	.03
	Time × Acceptance								5.92*	.05
	Time × Group								1.15	.01

ACT = Acceptance and Commitment Therapy

* *p* < .01

** p < .001

**Table 3:** Results of Repeated Measure ANOVA for coping styles as a moderator between the ACT and HbA1c, self-care and acceptance.

		Pre-treatment		Post-treatment		Follow-up		Repeated measure ANOVA		
Variables		*M (SD)*		*M (SD)*		*M (SD)*				
		ACT	Control	ACT	Control	ACT	Control	*df*	*F*	η^2^
**HbA1c**								1	987.65**	.92
Groups								1	35.42**	.28
Coping Styles Group	Effective	6.78 (1.28)	6.92 (1.16)	6.20 (0.96)	6.73 (1.34)	6.09 (0.92)	6.88 (1.43)			
	Combined	7.92 (1.25)	7.49 (1.11)	7.71 (1.14)	7.62 (1.16)	7.59 (1.07)	7.63 (1.09)	2	14.77**	.24
	Ineffective	7.84 (1.05)	8.26 (1.43)	7.62 (1.91)	8.48 (1.55)	7.61 (1.84)	8.70 (1.42)			
Group × Coping Styles Group								2	0.54	.01
**Self-care activities**								1	602.77**	.87
Groups								1	21.51**	.19
Coping Styles Group	Effective	78.02 (12.39)	85.22 (13.00)	92.32 (21.01)	88.28 (13.47)	93.57 (22.62)	90.88 (15.52)			
	Combined	60.53 (17.01)	68.09 (14.45)	61.32 (19.33)	68.54 (12.60)	62.07 (20.98)	67.82 (12.71)	2	6.50**	.12
	Ineffective	55.39 (16.59)	61.67 (16.29)	58.78 (18.53)	61.23 (16.43)	60.83 (23.32)	60.76 (16.55)			
Group × Coping Styles Group								2	3.69*	.07
**Acceptance & action diabetes**								1	621.59**	.87
Groups								1	73.98**	.44
Coping Styles Group	Effective	57.00 (12.24)	60.33 (13.54)	66.00 (11.83)	61.22 (12.97)	67.63 (10.81)	62.56 (12.99)			
	Combined	57.54 (14.19)	53.64 (18.49)	62.69 (10.61)	52.45 (17.28)	64.07 (14.43)	53.73 (18.34)	2	2.88 ^a^	.06
	Ineffective	43.22 (17.79)	48.62 (14.15)	54.78 (16.34)	47.19 (14.86)	56.11 (17.83)	47.00 (16.75)			
Group × Coping Styles Group								2	2.59 ^a^	.05

ACT = Acceptance and Commitment Therapy; *df* error = 93

a = marginal significance

* *p* < 0.01

** p < 0.001

## Results

### Descriptive statistics and corrolations

Socio-demographic characteristics, anthropometrics and medical data for the control and experimental groups and overall (*60% female*) participants are shown in Table 1; eighty-three percent were married, 15% widowed or divorced and 2% single. Diabetes duration ranged between 1 to 7 years (mean 4.22 years). Fifty-four percent had primary school education, 38% held a high school diploma, and 8% had college degrees. The two groups did not differ in body mass index, medication–insulin protocols or any other demographic variables. Correlation analyses showed significant negative correlations between glycated hemoglobin level and self-care activities (*r* = -0.62, *p* < .01), effective coping styles (*r* = -0.50, *p* < .01) and acceptance (*r* = -0.48, *p* < .01). Ineffective coping style was significantly and positively correlated with HbA1c levels (*r* = 0.24, *p* < .05).

### Impact ACT on self-management and its stability

A repeated Measure ANOVA for evaluating the effect of group ACT on self-management and acceptance indices, controlling for pretreatment scores (see Table 2), revealed significant effects for glycated hemoglobin (*F*_1,97_ = 32.36; *p* < .001; partial η^2^ = .25), self-care activities (*F*_1,97_ = 26.74; *p* < .001; partial η^2^ = .22) and acceptance (*F*_1,97_ = 76.75; *p* < .001; partial η^2^ = .44) scores. Patients undergoing group ACT obtained significantly lower glycated hemoglobin, higher self-care activities and higher acceptance scores than the control group. Also, results indicated that the effects of group ACT remained stable three months after completion of therapy. Means and standard deviations of all measures assessed at pre-, and post-treatment and follow- up are shown in Table 2.

### Moderation analysis

In order to examine whether coping style moderated the association of group ACT with glycated hemoglobin, self-care activities and acceptance, three separate repeated measure ANOVA analyses were performed. A repeated Measure ANOVA analysis for evaluating the moderating role of coping styles in the relationship between group ACT and three indices of diabetes control, while controlling for pretreatment scores (Table 3), yielded statistically significant results only for self-care activities (*F*_1,93_ = 3.69; *p* < .01; partial η^2^ = .07). In the other words, the interaction between ACT and coping style was found to be significant only in relation to self-care activities, confirming the hypothesis regarding the moderating role of coping style in the relationship between ACT and self-care activities. Results were marginally significant for acceptance (*F*_1,93_ = 2.59; *p* = .06; partial η^2^ = .05) and non-significant for glycated hemoglobin (*F*_1,93_ = 0.54; *p* = .58; partial η^2^ = .01). Means and standard deviations of all variables assessed at pre- and post-treatment and follow- up in all subgroups are shown in Table 3.

**Fig 2:**
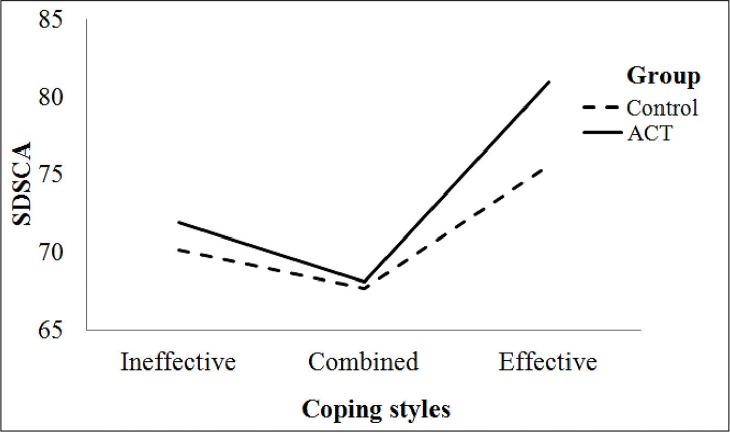
Moderator effects of coping styles on the relationship between acceptance and commitment therapy and self-care activates.

To further clarify the effects of significant interactions between ACT and coping style (Effective, Ineffective and Combined) on self-care activities, a simultaneous test procedure (STP) was performed. For this purpose three interaction factors, Group ACT × Effective Coping Style, Group ACT × Ineffective Coping Style, and Group ACT × Combined Coping Style were computed with self-care activities as the dependent variable. Results yielded one significant finding for Group ACT × Effective Coping Style (5 × *F*_0.15,5,97_/2 = 6.45), indicating that only effective coping style played a moderating role in the relationship between group ACT effects and self-care activities in T2DM patients; in other words, only those patients with an effective coping style significantly increased their self-care activities after undergoing ACT (Fig 2).

Fig 2 depicts changes in self-care activities from pre- to post-test in relation to the interaction of group ACT with each one of the three coping styles; as seen, only the group ACT × effective coping interaction term was associated with significant improvements in self-care activities; while the group ACT × ineffective coping style and the group ACT × combined coping style were not associated with such improvement. Furthermore, no significant differences in self-care activities among the three interactions terms (group ACT × coping styles), was observed in the control group. Given the current lack of data on moderators in the relationship between the ACT effect and diabetes control indices, these analyses were exploratory, but were not powered to make subgroup comparisons. Therefore, although these findings should be interpreted with caution, they none the less would be useful in future research.

## Discussion

The main purpose of the present research was to evaluate the effects of group-based ACT on self-management of patients with T2DM, considering the moderating role of coping styles. In general, the present study provides support for the hypothesized effect of ACT and offers additional evidence regarding mechanisms for the effectiveness of ACT in T2DM. Results indicated that group ACT in comparison with a one-day educational workshop was statistically more effective in increasing diabetes control indices of patients with T2DM. Moreover, group ACT effects on glycated hemoglobin remained stable after a 3-month follow-up period, a finding in line with those of the Gregg et al. research [3], which investigated the effectiveness of group ACT in improving glycated hemoglobin levels and self-care activities among 81 American patients (46.9% females; mean age 50.9 years) diagnosed with T2DM; they also found that group ACT effects remained stable after a three-month follow-up evaluation. The current clinical randomized control trial study emphasizes the importance of acceptance in self-management of diabetes. Initial emphasis on acceptance may facilitate later actions based on the values specified earlier, and in turn, acceptance and action based on these values improve performance [31]. Furthermore Hadlandsmyth et al. [32] in a study aimed at conceptualizing ACT concluded that ACT is a useful therapy for improving management of adolescent diabetes; they suggested that distressing personal experiences and avoidant behavior in patients with diabetes could lead to poor management of diabetes by reinforcing experiential avoidance and cognitive fusion, which are barriers to appropriate management of diabetes. Rosenzweig et al. [33] in a similar study provided evidence for the efficacy of mindfulness training in decreasing blood sugar among adults diagnosed with type 2 diabetes.

Moreover, additional findings of the present study indicate that a patient’s effective coping styles have a moderating role in the relationship between group ACT effects and self-care activities. Only the effective coping style and but not the ineffective or combined coping styles had a significant effect in strengthening the effects of group ACT for improving self-care activities. Coping strategies in changing life style conditions and the stress caused by these changes are intimately related to patients’ monitoring behavior, in particular to patients’ psychological states [34]. Fisher et al., [35] performed an evaluation of 186 research studies, published between 1990 and 2006, regarding the factors effective in diabetes management; in their review they highly emphasized healthy coping and found that in all the articles reviewed a notable relationship was found between coping styles, behavioral factors, quality of life and metabolic control. The common feature of effective coping strategies was dynamism. Dynamism provides requisite equipment’s for active coping with stressful situations. This condition may cause individuals to use cognitive and emotional skills flexibly for coping with problems and hassles, resulting in more satisfaction [36].

Yancura et al. [37] demonstrated that an effective coping style is a protective factor for metabolic energy consumption. Furthermore, diabetes self-care ability is affected by the individual patient’s adjustment with the disease and in turn the patient’s adjustment is considerably affected by her/his appropriate coping behavior [38].

In general, it is recognized that the utilization of effective coping strategies by T2DM patients is necessary and desirable [39]. Consequently, if patients rely on effective coping strategies in stressful situations, there is a higher probability that they will maintain their diet and that the stressors will not have a detrimental effect on their health [19]. According to the buffer model of stress, it is assumed that stressful conditions can detrimentally affect the involvement of the patients in self-care activities [14] and that the patient’s dominant coping style will moderate the effects of stress on self-care activities (diet, physical activity, and other disease- related recommendations) involvement, which in turn will exert an effect on metabolic energy functioning [17,40]. By identifying this moderator we are in position to gain further understanding of diabetes self-management in its broader sense.

Another result of the present research involved a direct effect of coping styles on glycated hemoglobin; although their moderating role in improving glycated hemoglobin, was not significant, Findings in line with those of the Graue et al. [41] study, which reported that effective coping strategies were directly related with better metabolic control of diabetes; in addition, in their study, poor metabolic control of T2DM patients was significantly associated with the use of ineffective coping strategies. Lustman and Gavard, [42] in their study showed that ineffective coping with stress and the use of ineffective coping styles had detrimental effects on the control of diabetes and therapy compliance. Coelho et al. [38] have also reported that ineffective coping strategies like avoidant coping are significantly associated with problems related to lack of adjustment to diabetes that had a negative impact on blood sugar control.

Some limitations of the present study need to be addressed. No follow-up assessment longer than 3-months was performed and the long-term effects of this interventional study could not be assessed, indicating the need for future studies to investigate these effects. It would be very interesting to see one-year and two-year follow-up assessments in intervention similar to the current one. Overall the main hypothesis of this study was confirmed, offering additional findings that elaborate on the therapeutic model used. The present study shows that effective coping style is more important than ineffective coping style in ACT for self-management of patients with T2DM.

## Supporting Information

S1 Checklist (DOC)

S1 Table Demographic and clinical characteristics of patients with type 2 diabetes. (DOCX)

S2 Table Results of Repeated Measure ANOVA for effect of the ACT on HbA1c, self-care and acceptance. (DOCX)

S3 Table Results of Repeated Measure ANOVA for coping styles as a moderator between the ACT and HbA1c, self-care and acceptance. (DOCX)

S1 Fig Attrition number for acceptance and commitment therapy and one-day workshop. (TIF)

S2 Fig Moderator effects of coping styles on the relationship between acceptance and commitment therapy and self-care activates. (TIF)

S1 CONSORT (DOC)

S1 Protocol (DOCX)

S2 Protocol (DOCX)
