# Supplementary material for: A Randomized Controlled Trial of Acceptance and Commitment Therapy for Type 2 Diabetes Management: The Moderating Role of Coping Styles
Source: PLoS One. 2016 Dec 1;11(12):e0166599. doi: 10.1371/journal.pone.0166599 (PMC5132195; doi:10.1371/journal.pone.0166599)
Supplement: S1 Table — (DOCX) [file pone.0166599.s002.docx]

**Table1.** Demographic and clinical characteristics of patients with type 2 diabetes (N=100)

| Characteristics | Groups | |  | |
| --- | --- | --- | --- | --- |
|  | ACT (*n* = 50) | Control (*n* = 50) | Overall | *p* |
| Age in years, *M (SD)* | 55.18 (8.26) | 55.70 (8.98) | 55.44 (8.44) | .76 |
| Gender: *n* (%) female | 33 (66) | 27 (54) | 60 (60) | .31 |
| Education, *n* (%) |  |  |  |  |
| High school and lower | 29 (58) | 25 (50) | 54 (54) |  |
| Diploma and Associate | 19 (38) | 19 (38) | 38 (38) | .32 |
| Bachelor and upper | 2 (4) | 6 (12) | 8 (8) |  |
| Marital status, *n* (%) |  |  |  |  |
| Single | 2 (4) | 0 (4) | 2 (2) |  |
| Married | 43 (86) | 40 (80) | 83 (83) | .15 |
| Widowed | 5 (10) | 10 (20) | 15 (83) |  |
| Diabetes duration in years, *M (SD)* | 4.90±1.40 | 4.54±1.54 | 4.22±1.49 | .70 |
| Body mass index in k/m^2^, *M (SD)* | 29.24±4.56 | 29.45±4.76 | 29.46±4.62 | .82 |
| Hypertension in mmHg, *M (SD)* | 13.25±2.01 | 13.66±1.93 | 13.46±1.96 | .30 |
| Insulin, *n* (%) | 1 (2) | 1 (2) | 2 (2) |  |
| Oral medication, *n* (%) | 36 (72) | 37 (74) | 73 (73) | .97 |
| Insulin + Oral medication, *n* (%) | 13 (26) | 12 (24) | 25 (25) |  |

*Note.* M±SD and number of subjects (%); ACT = Acceptance and Commitment Therapy; differences between ACT and control
