# Supplementary material for: A Randomized Controlled Trial of Acceptance and Commitment Therapy for Type 2 Diabetes Management: The Moderating Role of Coping Styles
Source: PLoS One. 2016 Dec 1;11(12):e0166599. doi: 10.1371/journal.pone.0166599 (PMC5132195; doi:10.1371/journal.pone.0166599)
Supplement: S2 Table — (DOCX) [file pone.0166599.s003.docx]

**Table 2.** Results of Repeated Measure ANOVA for effect of the ACT on HbA1c, self-care and acceptance

|  | | | Pretreatment | | Post-treatment | | Follow-up | | Repeated measure ANOVA | |
| --- | --- | --- | --- | --- | --- | --- | --- | --- | --- | --- |
| Variables | |  | *M (SD)* | | *M (SD)* | | *M (SD)* | | *F*_(1,97)_ | η^2^ |
|  | Group | ACT | 7.46 (1.66) | | 7.10 (1.56) | | 7.03 (1.52) | | 32.36^**^ | .25 |
|  |  | Control | 7.61 (1.38) | | 7.66 (1.57) | | 7.81 (1.56) | |  |  |
| HbA1c | Time | |  | |  | |  | | 5.64^*^ | .05 |
|  | Time × HbA1c | |  | |  | |  | | 4.66^*^ | .05 |
|  | Time × Group | |  | |  | |  | | 16.37^**^ | .22 |
|  | Group | ACT | 65.34 (18.11) | | 72.32 (24.98) | | 73.60 (27.32) | | 26.74^**^ | .22 |
|  |  | Control | 71.56 (17.96) | | 72.58 (18.84) | | 73.16 (20.40) | |  |  |
| Self-care | Time | |  | |  | |  | | 2.65 | .03 |
|  | Time × Self-care | |  | |  | |  | | 4.17^*^ | .04 |
|  | Time × Group | |  | |  | |  | | 0.74 | .01 |
|  | Group | ACT | 52.18 (16.14) | | 61.10 (14.01) | | 62.56 (15.17) | | 76.75^**^ | .44 |
|  |  | Control | 53.94 (15.57) | | 53.40 (15.77) | | 54.08 (17.0) | |  |  |
| Acceptance and action diabetes | Time | |  |  |  |  |  |  | 2.57 | .03 |
|  | Time × Acceptance | |  |  |  |  |  |  | 5.92^*^ | .05 |
|  | Time × Group | |  |  |  |  |  |  | 1.15 | .01 |

ACT = Acceptance and Commitment Therapy; * *p* < .01, *** p* < .001
