# Supplementary material for: A Randomized Controlled Trial of Acceptance and Commitment Therapy for Type 2 Diabetes Management: The Moderating Role of Coping Styles
Source: PLoS One. 2016 Dec 1;11(12):e0166599. doi: 10.1371/journal.pone.0166599 (PMC5132195; doi:10.1371/journal.pone.0166599)
Supplement: S3 Table — (DOCX) [file pone.0166599.s004.docx]

**Table 3.** Results of Repeated Measure ANOVA for coping styles as a moderator between the ACT and HbA1c, self-care and acceptance

|  | | Pre-treatment | | Post-treatment | | Follow-up | | Repeated measure ANOVA | | |
| --- | --- | --- | --- | --- | --- | --- | --- | --- | --- | --- |
| Variables | | *M (SD)* | | *M (SD)* | | *M (SD)* | |  |  |  |
|  | | ACT | Control | ACT | Control | ACT | Control | *df* | *F* | η^2^ |
| **HbA1c** | |  |  |  |  |  |  | 1 | 987.65^**^ | .92 |
| Groups | |  |  |  |  |  |  | 1 | 35.42^**^ | .28 |
| Coping Styles Group | Effective | 6.78 (1.28) | 6.92 (1.16) | 6.20 (0.96) | 6.73 (1.34) | 6.09 (0.92) | 6.88 (1.43) |  |  |  |
|  | Combined | 7.92 (1.25) | 7.49 (1.11) | 7.71 (1.14) | 7.62 (1.16) | 7.59 (1.07) | 7.63 (1.09) | 2 | 14.77^**^ | .24 |
|  | Ineffective | 7.84 (1.05) | 8.26 (1.43) | 7.62 (1.91) | 8.48 (1.55) | 7.61 (1.84) | 8.70 (1.42) |  |  |  |
| Group × Coping Styles Group | | |  |  |  |  |  | 2 | 0.54 | .01 |
| **Self-care activities** | |  |  |  |  |  |  | 1 | 602.77^**^ | .87 |
| Groups | |  |  |  |  |  |  | 1 | 21.51^**^ | .19 |
| Coping Styles Group | Effective | 78.02 (12.39) | 85.22 (13.00) | 92.32 (21.01) | 88.28 (13.47) | 93.57 (22.62) | 90.88 (15.52) |  |  |  |
|  | Combined | 60.53 (17.01) | 68.09 (14.45) | 61.32 (19.33) | 68.54 (12.60) | 62.07 (20.98) | 67.82 (12.71) | 2 | 6.50^**^ | .12 |
|  | Ineffective | 55.39 (16.59) | 61.67 (16.29) | 58.78 (18.53) | 61.23 (16.43) | 60.83 (23.32) | 60.76 (16.55) |  |  |  |
| Group × Coping Styles Group | | |  |  |  |  |  | 2 | 3.69^*^ | .07 |
| **Acceptance & action diabetes** | | |  |  |  |  |  | 1 | 621.59^**^ | .87 |
| Groups | |  |  |  |  |  |  | 1 | 73.98^**^ | .44 |
| Coping Styles Group | Effective | 57.00 (12.24) | 60.33 (13.54) | 66.00 (11.83) | 61.22 (12.97) | 67.63 (10.81) | 62.56 (12.99) |  |  |  |
|  | Combined | 57.54 (14.19) | 53.64 (18.49) | 62.69 (10.61) | 52.45 (17.28) | 64.07 (14.43) | 53.73 (18.34) | 2 | 2.88 ^a^ | .06 |
|  | Ineffective | 43.22 (17.79) | 48.62 (14.15) | 54.78 (16.34) | 47.19 (14.86) | 56.11 (17.83) | 47.00 (16.75) |  |  |  |
| Group × Coping Styles Group | | |  |  |  |  |  | 2 | 2.59 ^a^ | .05 |

ACT = Acceptance and Commitment Therapy; *df* error = 93; a = marginal significance; * *p* < 0.01, *** p* < 0.001
