# Supplementary figures and images for: A Randomized Controlled Trial of Acceptance and Commitment Therapy for Type 2 Diabetes Management: The Moderating Role of Coping Styles
Source: PLoS One. 2016 Dec 1;11(12):e0166599. doi: 10.1371/journal.pone.0166599 (PMC5132195; doi:10.1371/journal.pone.0166599)

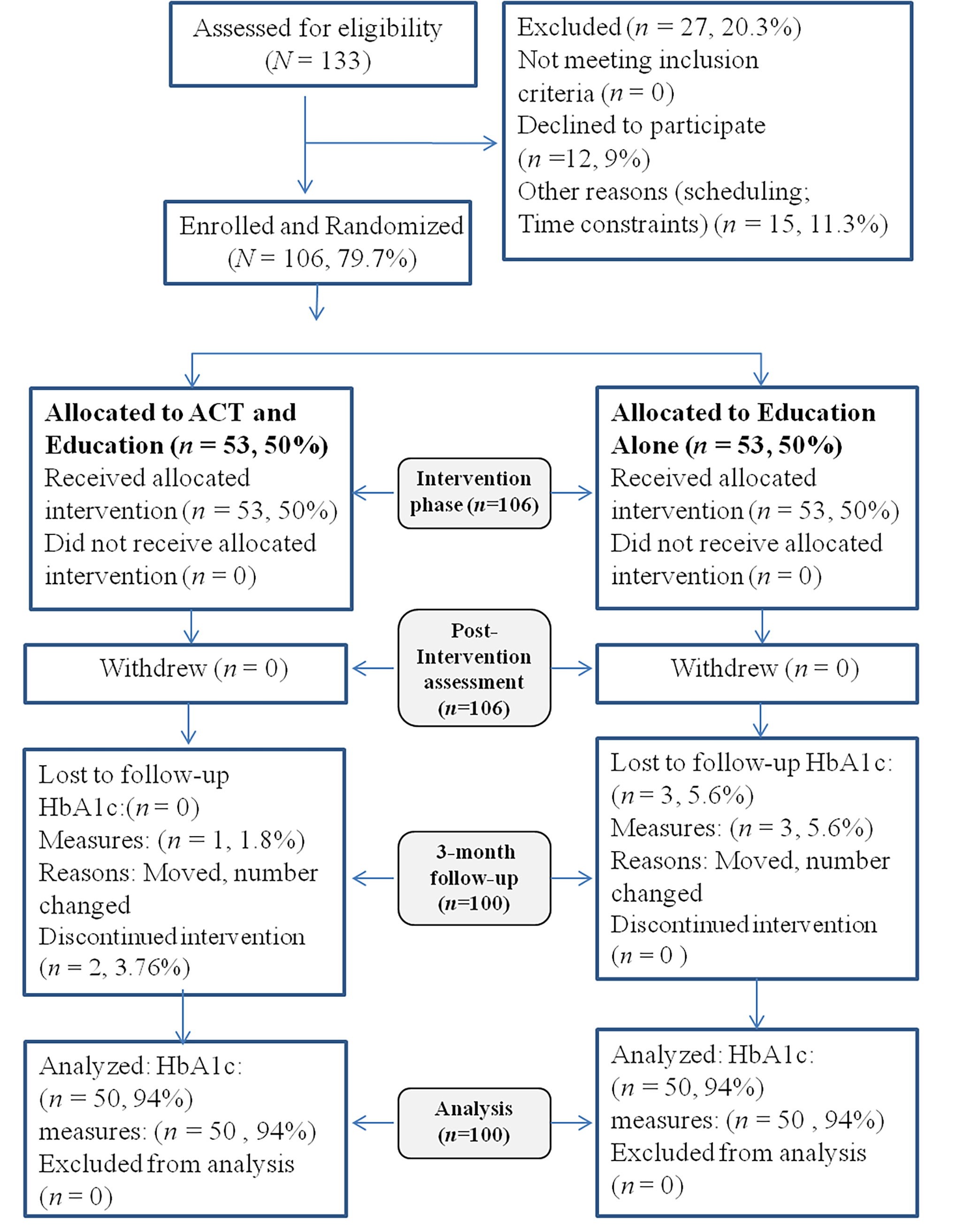

Supplement: S1 Fig — (TIF) [file pone.0166599.s005.tif]

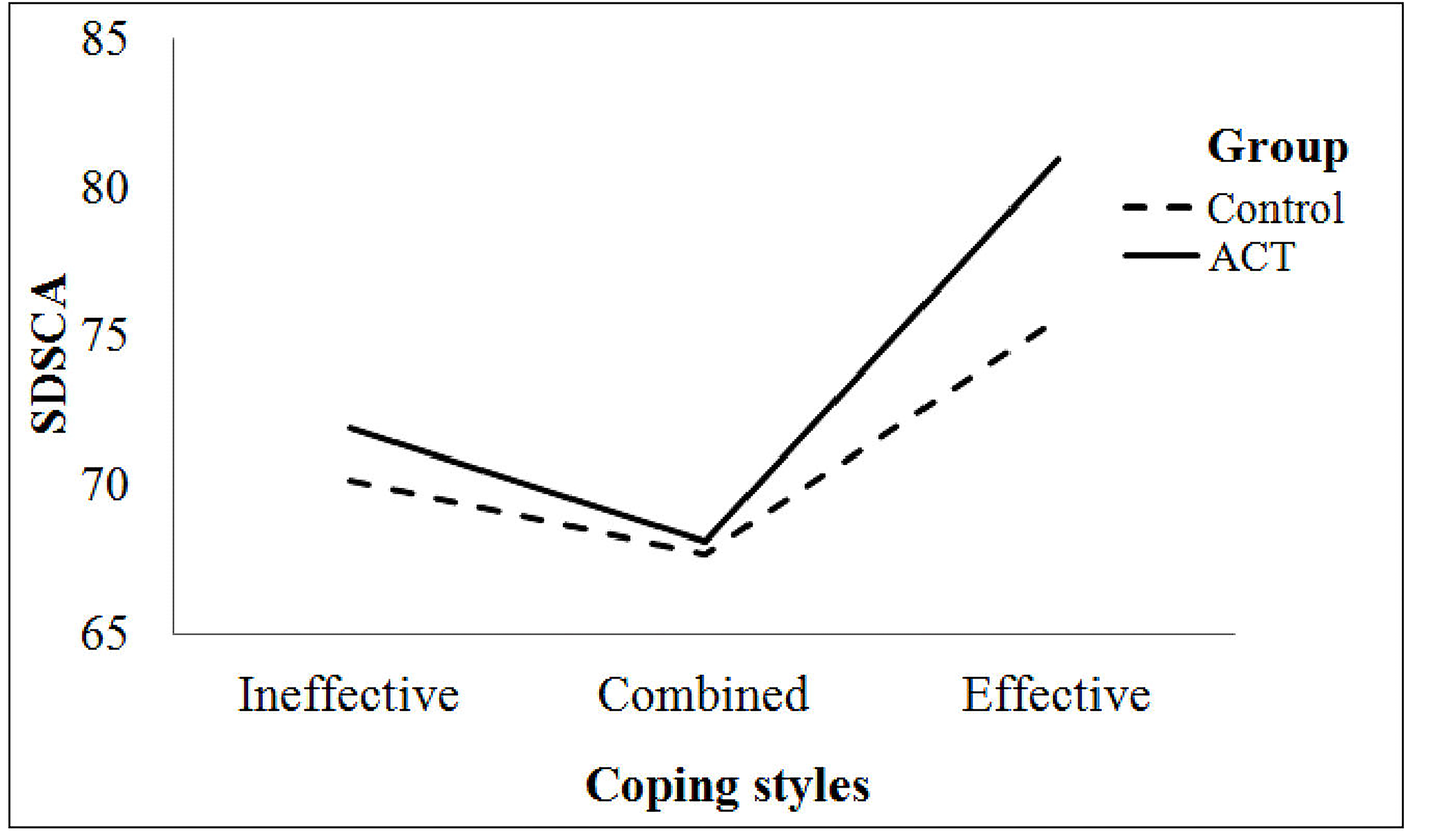

Supplement: S2 Fig — (TIF) [file pone.0166599.s006.tif]
