# Supplementary material for: A Randomized Controlled Trial of Acceptance and Commitment Therapy for Type 2 Diabetes Management: The Moderating Role of Coping Styles
Source: PLoS One. 2016 Dec 1;11(12):e0166599. doi: 10.1371/journal.pone.0166599 (PMC5132195; doi:10.1371/journal.pone.0166599)
Supplement: S1 CONSORT — (DOC) [file pone.0166599.s007.doc]

**
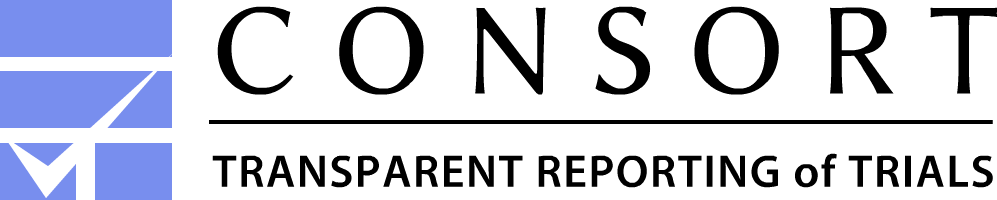
**

**CONSORT 2010 Flow Diagram**

**Allocation**

**Analysis**

**Follow-Up**

**Enrollment**

Assessed for eligibility (n=133)

Excluded (n=27, 20.3%)

♦  Not meeting inclusion criteria (n=0)

♦  Declined to participate (n=12, 9%)

♦  Other reasons (n=15,11.3%)

Analysed (n=50, 94%)
♦ Excluded from analysis (n=0)

Lost to follow-up (Moved) (n=1, 1.8%)

Discontinued intervention (number changed) (n=2, 3.76%)

Allocated to intervention (n=53, 50%)

♦ Received allocated intervention (n= n=53,50%)

♦ Did not receive allocated intervention (n=0)

Lost to follow-up (take care patients at home, changing residence location) (n=3. 5.6%)

Discontinued intervention (give reasons) (n=0)

Allocated to intervention (n=53, 50%)

♦ Received allocated intervention (n= n=53,50%)

♦ Did not receive allocated intervention (n=0)

Analysed (n=50, 94%)
♦ Excluded from analysis (n=0)

Randomized (n=106, 79.7%)
