# Supplementary material for: A Randomized Controlled Trial of Acceptance and Commitment Therapy for Type 2 Diabetes Management: The Moderating Role of Coping Styles
Source: PLoS One. 2016 Dec 1;11(12):e0166599. doi: 10.1371/journal.pone.0166599 (PMC5132195; doi:10.1371/journal.pone.0166599)
Supplement: S1 Protocol — (DOCX) [file pone.0166599.s008.docx]

**Clinical trial protocol**

**Group Acceptance and Commitment Therapy for Self-Management of Adult Patients with Type 2 Diabetes**

**Primary Investigator:**

Zeinab Shayeghian, PhD

Parisa Amiri, PhD

**TRIAL COORDINATORS:**

Kobra roohi gillani, MD.

**SUPERVISORS:**

Maria E. Aguilar-Vafaie, PhD

Mohammad Ali Besharat, PhD

Hamidreza Hassanabadi, PhD

Table of Contents

1.0 Introduction 3

2.0 Study Overview 4

2.1.1 Methodology 4

2.1.2 Components of the Treatment 5

2.1.3 Design 7

2.1.4 Patient Selection and Inclusion/Exclusion Criteria 7

2.1.5 Sample size determination 8

2.1.6 Statistical analysis 8

3.0 Justification of methodology 8

4.0 Feasibility 9

4.1.1 Study committe 9

4.1.2 Setting 9

4.1.3 Time Line 10

5.0 Ethics 10

6.0 Funding 10

Appendix A: Brief Cope 11

Appendix B: SDSCA 12

Appendix C: AADQ 13

References ….14

1. **INTRODUCTION**

According to the American Diabetes Association (2013) the economic costs of diabetes are high. The estimated total economic cost of diagnosed diabetes in 2012 is $245 billion, a 41% increase from our previous estimate of $174 billion (in 2007 dollars). Type 2 diabetes is a chronic disease associated with a ten-year-shorter life expectancy and the most common form of diabetes (Paris et al, 2001).This is partly due to a number of complications with which it is associated, including: two to four times the risk of [cardiovascular disease](https://en.wikipedia.org/wiki/Cardiovascular_disease), including [ischemic heart disease](https://en.wikipedia.org/wiki/Ischemic_heart_disease) and [stroke](https://en.wikipedia.org/wiki/Stroke); a 20-fold increase in lower limb [amputations](https://en.wikipedia.org/wiki/Amputations), and increased rates of [hospitalizations](https://en.wikipedia.org/wiki/Hospitalization) (Melmed et al, 2011). Diabetes control requires continuing medical care and patient self-management education to reduce the risk of long-term and acute complications (Jaser et al, 2012).

In addition to medical treatment, many kinds of psychological interventions have been used in the control of diabetes, including self-care education (Funnell & Anderson, 2004), behavior therapy (Tate, Jackvony & wing, 2003), and cognitive-behavior therapy (Snoke et al, 2001); these have been effective, but unfortunately mostly for short periods of time (Rubin & Napora, 2001); Furthermore, previous research studies have been conducted with no clear depiction of the real social and cultural situations and contexts making it difficult for those working in other healthcare settings to adapt the program to their own situations (Bastiaens et al, 2009).

Acceptance and commitment therapy in comparison to other interventions is more consistent with the chronic nature of the diabetes. it seems impossible to eliminate thoughts related to diabetes that are part of the very nature of the disease, However, most psychological studies involving the application of cognitive-behavior therapy have concentrated on decreasing, changing or stopping negative thoughts related to diabetes, but, Acceptance and commitment therapy focuses on the acceptance of thoughts that emphasize clarification of values and personal goals (Gregg et al, 2007).

Acceptance and commitment therapy were used successfully to lifestyle changes in behavioural problems (Hayes et al, 2004 ; Niemeier et al, 2012) but, to date only a limited number of studies has been published investigating the effects of Acceptance and commitment therapy on diabetes self-management (e.g. Gregg et al, 2007), emphasizing the need for related data. In spite of different studies on acceptance and commitment therapy, the role of moderator variables in the process of testing the effectiveness of ACT in diabetes control has not been addressed in the literature. Therefore, our purpose is assessment of one of the important moderators (coping style) in the effect of psychotherapy on self-management. Promising results support the continued use and development of the method.

***Purpose and benefits of the study***

This study has two purpose:1- To examine whether acceptance and commitment therapy works for participants with a diagnosis of type 2 diabetes and whether it provides clinically significant improvement in HbA1c reduction and improvement in self-management compared with the control group. 2- To assess the moderating role of coping styles in the relationship between acceptance and commitment therapy and self-management of patients with type 2 diabetes.

Perhaps the main contribution the study could give is that it opens up for other psychological modator and mediators in the effect of acceptance and commitment therapy on development of diabetes control. Therefore, this study may become important. this will be important information as many Mindfulness-Oriented Therapist already engaged in acceptance and commitment therapy

1. **PROPOSED STUDY**

This randomised controlled trial will evaluate the efficacy of acceptance and commitment therapy to effectively control diabetes. This will be an intervention trial using treatments and one-day educational workshop. The study sample will be randomised into two arms: treatment and control. The study population will consist of 100 patients with type 2 diabetes.

## Study Overview

### 2.1.1 Methodology

Setting and Participants: The setting for this study will be the Endocrine Department of Labbafinejad Hospital in Tehran. The study will involve patients with type 2 diabetes. The study population will be male and female, in the age groups from 40 to 60. Sampling and Recruitment: Recruitment for the study will be facilitated by Labbafinejad Hospital. Participants will be introduced to researchers by the endocrinologist from the endocrine department. Interested parties will be encouraged to contact the psychologist directly and provide their name, phone number and preferred time of contact. Interested participants will be given an Information Kit, which will include: a sample consent form, a Backgrounder explaining the purpose of the study and contact list and phone numbers of the psychologist and researchers involved in the study.

Interested participants will be encouraged to ask questions to their Endocrinologist and directly to the Psychologist. The participants will be introduced to researchers by the endocrinologist from the endocrine department to participate in a 1-day educational workshop for self-management of diabetes,and all participants will be received a one-day workshop about diabetes control and will be encouraged to complete several questionnaires on socio-demographic characteristics, Summary of Diabetes Self-Care Activities (SDACA), Beck Depression Inventory, Acceptance and Action Diabetes Questionnaire and Brief COPE Questionnaire. then weight and height will be measured and blood samples will be obtained. Eligible participants will be randomly assigned to either the control or treatment groups (using the random number table), and will be booked for further appointment times.

On subsequent visits, the intervention group patients will receive treatments including group acceptance and commitment therapy (ACT) for diabetes management in addition to the one day workshop. the ACT will be delivered during 10, 2-hour sessions. the control group patients will receive one day workshop to help diabetes control and they will be remained in a waiting list and will be received same treatment after the end of study (because of clinical ethical concern, but these result will not be entered into analysis). In one day workshop, both groups will be encouraged to engage in an active exercise routine. Both groups continue will be medications (e.g., Metformin), but they can be not changed medications and dosages during the study. Information of both groups will be obtained by questionnaires and results from blood tests at the points, before beginning therapy, after completing therapy sessions, and last, at a three-month follow-up. Blood test assessments will be provided for free as an incentive for participation.

**2.1.2 Components of the Treatment**

The group acceptance and commitment therapy (ACT) will be delivered during 10, 2-hour sessions and each session of study is one week.

Across problems in living, ACT is a treatment that is difficult to deliver with integrity. The application of ACT to diabetes is no different, and contains specific challenges as well. The goal of the treatment is to compassionately assist patients in dealing with any thoughts, feelings, worries, anxieties, or fears they may have related to their disease and its maintenance in order to help them live their life in a valued direction related to their diabetes. A key first step in this holistic approach to diabetes self-management is to provide adequate information about how to care for one’s diabetes in order to address the specific motivational and acceptance pieces laid out in the treatment. Thus, the first half of the treatment provides specific information regarding the management of diabetes and the second half of the treatment addresses motivational and acceptance issues related to engaging in self-management behaviors. The treatment is divided into 5 main modules:

Module I: Education and Information

Module II: Food, Diabetes, and Your Health

Module III: Exercise and Diabetes

Module IV: Coping and Stress Management

Module V: Acceptance and Action

Each module can be compared to two treatment session and will be administered at weekly intervals. In the first three modules, patients are given broad information about diabetes and its complications, and are given specific information about the effects of high and low glucose on one’s body. In the second module, this information is tied specifically to the intake of food and information is provided about how to eat in order to prevent surges and dips in blood glucose. In the third module, information is provided about the impact of exercise on blood glucose, and setting up an exercise routine. Once the broad and specific information on diabetes is given, the second half of the treatment addresses more psychological and motivational barriers to effective self-management. The fourth module addresses avoidance coping as it relates to diabetes, and particularly addresses avoidance of negativelyevaluated thoughts and feelings in diabetes, and how they may interfere with effective self-management. The fifth module integrates the didactic and acceptance elements of the treatment and focuses patients on moving in the direction of their values. In addition to being specifically addressed in the fifth module, values are targeted throughout the treatment and are a major component of the intervention. Specifically, directing patients toward values-directed behavior change in diabetes self-management is the overall task of the treatment, with diabetes-related information and information about accepting negatively-evaluated thoughts and feelings (Gregg, 2004; available at http://www.psych.sjsu.edu/_jgregg).

Overall structure for each session:

| Session | Activity & Exercises | Purposes |
| --- | --- | --- |
| 1 | A general introduction of all the members of the group  Activity: self-assessment of diabetes care | Member recognition to facilitate of therapeutic relationship  Identification of information level of group about diabetes control |
| 2 | Share nutritional information in Group  Exercise: Values and choices in nutrition | Internalization of nutritional information and its effect on blood sugar |
| 3 | Share physical activity information in Group  Exercise: A choice of physical activity | Internalization of physical activity Information and its effect on blood sugar |
| 4 | Share information about value of diabetes control in Group  Exercise: Talking with your tombstone | Cognition of key values and goals in diabetes control and specific actions related to those goals |
| 5 | A discussion about values  Tichener's Milk, Milk, Milk Exercise | Improving control of diabetes on the basis of values or the ability to distinguish the literal meaning of words and values |
| 6 | A discussion about experiential avoidance  Exercise: The Polygraph Metaphor | Recognizing negative thoughts and process of experiential avoidance |
| 7 | Share the experience of cognitive avoidance  Exercise: Chinese Handcuff Metaphor | Improving recognizing experiential avoidance and appropriate coping |
| 8 | A discussion about acceptance of negative thoughts related to disease  Exercise: Chessboard Metaphor | Internalization of acceptance process during self-care activities and adjusting to diabetes |
| 9 | A discussion about the ingredients of acceptance  Activity: goal identification | Improving acceptance of diabetes-related negative feeling and thoughts |
| 10 | A discussion about commitment to managing diabetes  Activity: Stand and commitment to change behavior | Make a commitment to managing your diabetes |

Follow-up data will be collected at 3 month post treatment via questionnaire package containing Summary of Diabetes Self-Care Activities (SDACA), Acceptance and Action Diabetes Questionnaire and Brief COPE Questionnaire. Generally, endocrinlogist appointments is every 3 months, therefore appointments will be synchronized with time of post test and follow up that it will be faciliteted the collection of data.

### 2.1.3 Design

Single blinded, randomised, controlled intervention trial..

### 2.1.4 Patient Selection and Inclusion/Exclusion Criteria

one hundred patients with type 2 diabetes will be introduced to researchers by the endocrinologist. Therefore, Participants will be chosen based on convenience sampling procedure Participants will be selected according to the following criteria:

**Inclusion criteria**

1) Age 40-60 at study entry

2) Men and women

3) Written and informed consent.

4) Patients must have an Endocrinologist that they have seen in every 3 months.

**Exclusion criteria**

1) Have other kinds of diabetes (type 1 and Gestational diabetes)

2) If they had been hospitalized

3) Lack of satisfaction could not collaborate or continue in the study.

4) Any current serious disorders determined to be clinically significant to the study.

**2.1.5 Sample size determination**

According to a meta-analysis study in diabetes interventions; Brown (1990), the required sample size, based on a power of 80% and a 2-tailed α of 0.05, was calculated 35 individuals per group. However, assuming a 10% loss to follow-up rate, we need 45 subjects per group to commence the trial.

**2.1.6 Statistical Analysis**

# Demographic characteristics among two groups will be compared using chi-square test and student’s t-test. In order to assess the effects of the group ACT intervention, differences between the two groups for glycated hemoglobin, self-care activities score and acceptance will be assessed, after controlling for pre-test scores on these variables and For this purpose, three one-way covariance analyses will be computed. Then, In order to assess the stability of the therapy, a repeated measures analysis of covariance with post-test and follow-up scores will be computed, controlling for pre-test effects. Finally, in order to evaluate the moderating role of coping styles in the relationship between group ACT with glycated hemoglobin, self-care activities and acceptance, three separate two-way analyses of covariance will be computed.

# Justification of Methodology

The study trial shall involve 100 patients with type 2 diabetes as determined above. The trial shall last 22 weeks (10 weeks treatment time and 12 weeks follow-up) which we realized enough time to determine whether a clinically significant effect will be produced. We employed a randomized controlled design to determine if any change is significantly different from a control group not receiving the acceptance and commitment therapy. Control group allow us to determine if variability in diabetes control within individuals has overshadowed the impact of potential confounders. Examples of factors that could influence variability within individuals include age, sex, educational level, diabetes duration and medical treatment.

# Feasibility

**3.1.1 Study committee:**

Mohammad Ali Besharat, PhD. is a licensed clinical Psychologist. He has extensive experience consulting and psychological treatments and he has experience in trial design and conduct, interviewing skills and clinical skills.

Parisa Amiri, PhD, is a Health Educator. She has extensive experience in eduacational and clinical research..

Kobra roohi gillani, MD.is a Endocrinologist and is a Clinical Trial Coordinator at labafinejad hospital. She has experience in trial design and conduct, drug efficacy studies and clinical skills.

Hamidreza Hassanabadi, PhD. is a Educational Psychologist and is an expert in statistics. He has experience in trial design, research methodology and design.

Maria E. Aguilar-Vafaie, PhD, She has experience in trial design and conduct, drug efficacy studies, interviewing skills, and clinical skills.

Zeinab Shayeghian, PhD. is a Health Psychologist with clinical and research experience.

**3.1.2 Setting:**

The Endocrinology department of Labbafinejad hospital is an ideal center from which to adequately supervise and conduct the study. Study participants will be patients with type 2 diabetes recruited from Endocrinology department through specialist referral to researchers and talks led by the trial investigators. Participants will be fully informed of the treatment they may receive and must give informed consent before enrolment.

**3.1.3 TIME LINE**

The subjects will be directly involved in the study for twelve weeks. We intend on recruiting for the trial immediately upon approval of the trial by Ethics Committee of the Research Institute for Endocrine Sciences, Shahid Beheshti University of Medical Sciences.

Recruitment should take three to six weeks and if possible be complete early February. We will commence with treatment after recruitment of eligible participants, which is projected to end of February. The trial itself will be completed for 6 months.

1. **ETHICS**

We have passed this study through the Ethics Committee of the Research Institute for Endocrine Sciences, Shahid Beheshti University of Medical Sciences for ethics approval. Informed consent will be obtained from all enrolled subjects. All authors declare that they have no conflict of interest.

1. **FUNDING**

Part of the financial support for this project is provided by Tarbiat Modares University.

**APPENDIX A**

**Brief COPE**

These items deal with ways you've been coping with the stress in your life since you found out you were going to have to have this operation.  There are many ways to try to deal with problems.  These items ask what you've been doing to cope with this one.  Obviously, different people deal with things in different ways, but I'm interested in how you've tried to deal with it.  Each item says something about a particular way of coping.  I want to know to what extent you've been doing what the item says.  How much or how frequently.  Don't answer on the basis of whether it seems to be working or not—just whether or not you're doing it.  Use these response choices.  Try to rate each item separately in your mind from the others.  Make your answers as true FOR YOU as you can.

 1 = I haven't been doing this at all
 2 = I've been doing this a little bit
 3 = I've been doing this a medium amount
 4 = I've been doing this a lot

| 1.  I've been turning to work or other activities to take my mind off things.  2.  I've been concentrating my efforts on doing something about the situation I'm in.  3.  I've been saying to myself "this isn't real.".  4.  I've been using alcohol or other drugs to make myself feel better.  5.  I've been getting emotional support from others.  6.  I've been giving up trying to deal with it.  7.  I've been taking action to try to make the situation better.  8.  I've been refusing to believe that it has happened.  9.  I've been saying things to let my unpleasant feelings escape.  10.  I’ve been getting help and advice from other people.  11.  I've been using alcohol or other drugs to help me get through it.  12.  I've been trying to see it in a different light, to make it seem more positive.  13.  I’ve been criticizing myself.  14.  I've been trying to come up with a strategy about what to do.  15.  I've been getting comfort and understanding from someone.  16.  I've been giving up the attempt to cope.  17.  I've been looking for something good in what is happening.  18.  I've been making jokes about it.  19.  I've been doing something to think about it less, such as going to movies,   watching TV, reading, daydreaming, sleeping, or shopping.  20.  I've been accepting the reality of the fact that it has happened.  21.  I've been expressing my negative feelings.  22.  I've been trying to find comfort in my religion or spiritual beliefs.  23.  I’ve been trying to get advice or help from other people about what to do.  24.  I've been learning to live with it.  25.  I've been thinking hard about what steps to take.  26.  I’ve been blaming myself for things that happened.  27.  I've been praying or meditating.  28.  I've been making fun of the situation. |  |
| --- | --- |

**Appendix B**


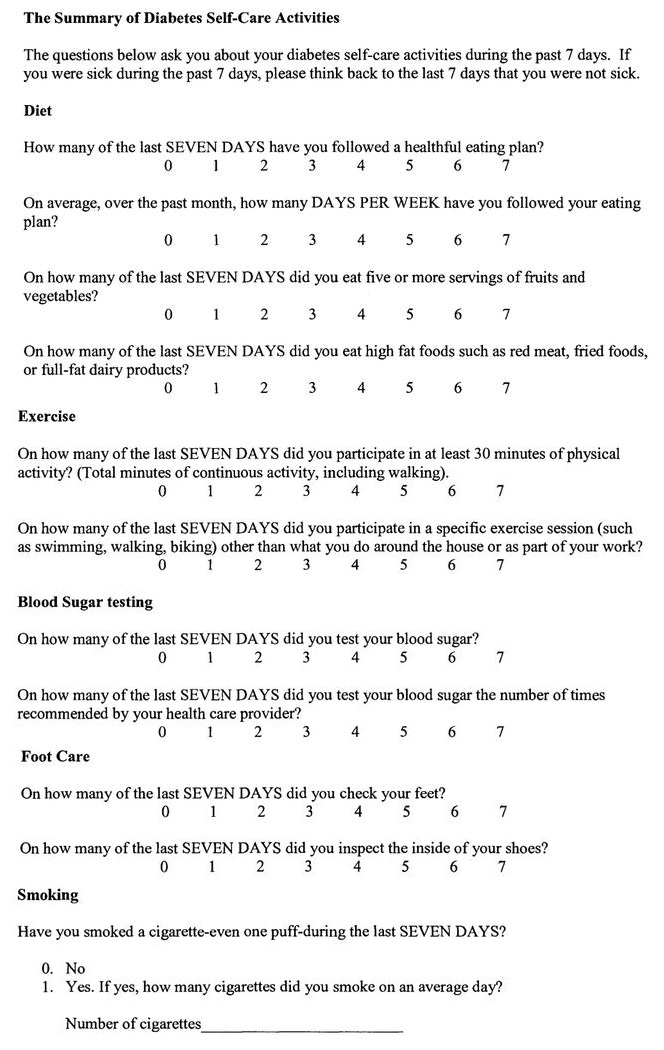


**Appendix c**

**The Acceptance and Action Diabetes Question**

**REFERENCES**

American Diabetes Association. (2013). Economic Costs of Diabetes in the U.S. in 2012. *Diabetes* Care, 36, 4, 1033-1046.

Brown SA. (1990). Studies of educational interventions and outcomes in diabetic adults: a meta-analysis revisited. Patient education and counseling, 16(3):189-215.

Cohen J. (1992). A power primer. Psychological bulletin.112(1):155.

Paris, R. M., Bedno, S. A., Krauss, M. R., Keep, L. W., & Rubertone, M. V. (2001). Weighing in on type 2 diabetes in the military: characteristics of U.S. military personnel at entry who develop type 2 diabetes. *Diabetes Care,24*, 1894–1898.

Melmed, S., Polonsky, K. S., Larsen, P. R., & Kronenberg H. M. (2011).*Williams textbook of endocrinology*. (12th ed.). Philadelphia, PA: Saunders Elsevier.

Jaser, S. S., Faulkner, M .S., Whittemore, R., Jeon, S., Murphy, K., Delamater, A., et al. (2012). Coping, self-management, and adaptation in adolescents with type 1 diabetes. *Annals of behavioral medicine : a publication of the Society of Behavioral Medicine, 43*, 311-9.

Funnell, M. M. & Anderson, R. M. (2004). Empowerment and self-management of diabetes. *Clinical diabetes,* 22, 123-7.

Tate, D. F., Jackvony, E. H., & Wing, R. R. (2003). Effects of Internet behavioral counseling on weight loss in adults at risk for type 2 diabetes: a randomized trial. *JAMA, 289,* 1833-1836.

Snoek, F. J., van der Ven, N. C., Lubach, C. H., Chatrou, M., Ader, H. J., Heine, R. J., et al. (2001). Effects of cognitive behavioural group training (CBGT) in adult patients with poorly controlled insulin-dependent (type 1) diabetes: a pilot study. *Patient education and counseling, 45,* 143-148

Rubin, R. & Napora, J. (2001). *Behavior change*. In: J. F. Marion KP, W. H. Polonsky, P. Yarborough, & V. Zamudio (Eds), A Core Curriculum for Diabetes Education, editor. Chicago: IL: American Association of Diabetes Educators.

Bastiaens, H., Sunaert, P., Wens, J., Sabbe, B., Jenkins, L., Nobels, F., et al. (2009). Supporting diabetes self-management in primary care: pilot-study of a group-based programme focusing on diet and exercise. *Primary care diabetes, 3,* 103-109.

Gregg, J. A., Callaghan, G. M., Hayes, S. C., Glenn-Lawson, J. L. (2007). Improving diabetes self-management through acceptance, mindfulness, and values: a randomized controlled trial. *Journal of consulting and clinical psychology, 75,* 336-43.

Hayes, S. C., Wilson, K. G., Gifford, E. V., Bissett, R., Piasecki, M., Batten, S. V. & et al. (2004). A preliminary trial of twelve-step facilitation and acceptance and commitment therapy with polysubstance-abusing methadone-maintained opiate addicts. *Behavior therapy, 35*, 4, 667-88.

Niemeier, H. M., Leahey, T., Reed, K. P., Brown, R. A., & Wing, R. R. (2012). An acceptance-based behavioral intervention for weight loss: a pilot study. *Behavior therapy, 43*, 2,427-35.
