# Supplementary material for: A Randomized Controlled Trial of Acceptance and Commitment Therapy for Type 2 Diabetes Management: The Moderating Role of Coping Styles
Source: PLoS One. 2016 Dec 1;11(12):e0166599. doi: 10.1371/journal.pone.0166599 (PMC5132195; doi:10.1371/journal.pone.0166599)
Supplement: S2 Protocol — (DOCX) [file pone.0166599.s009.docx]

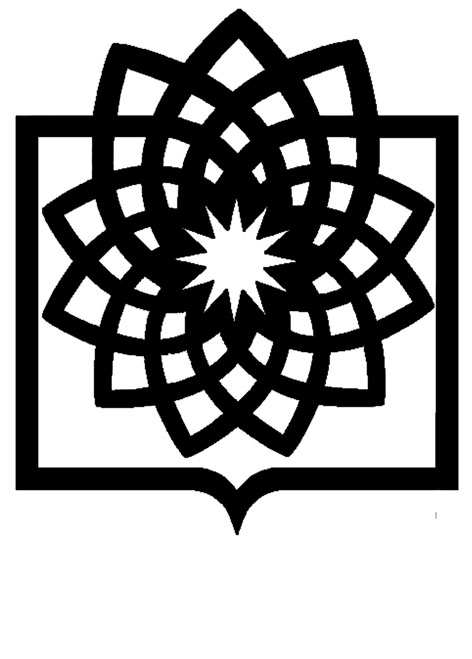
بنام خداوند بخشنده مهربان

###### جمهوري اسلامي ايران

### وزارت بهداشت , درمان و آموزش پزشكي

**دانشگاه علوم پزشكي وخدمات بهداشتي درماني شهيد بهشتي**


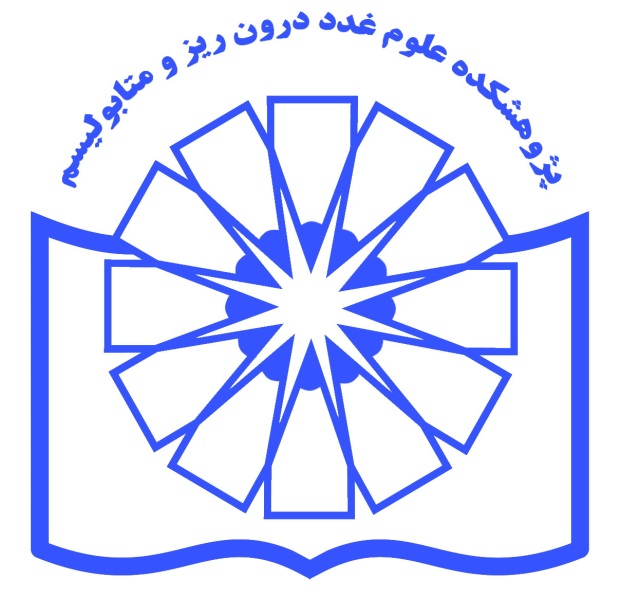
پژوهشکده علوم غدد درون ريز و متابوليسم

معاونت پژوهشي – مديريت امورپژوهش

فرم پيش نويس طرح پژوهشي

(PROPOSAL)

| **عنوان**: نقش تعدیل­کننده سبکهای مقابله­ و حمایت اجتماعی در تأثیر درمان گروهی پذیرش و تعهد بر هموگلوبین گلیکوزیله، کیفیت زندگی و خودمراقبتی در بیماران مبتلا به دیابت نوع II |
| --- |
| **Title:** **The Moderating role of coping style and social support in effect of ACT on HbA1c, quality of life and self care in type II diabetics** |

نام و نام خانوادگي طرح دهندگان^1^ :

**دکتر پریسا امیری، دکتر زینب شایقیان**

دانشكده/ مركز تحقيقاتي: پژوهشکده علوم غدد درون ریز و متابولیسم، مرکز تحقیقات پیشگیری و درمان بیماری های متابولیک

قسمت اول- توضيح نكات لازم و ضروري

توجه : پاسخ بسياري از پرسشهاي شما درباره مندرجات اين فرم در مطالب زير ارائه شده است، بنابراين خواهشمند است قبل از تكميل اين فرم مطالب زير رابه دقت مطالعه فرموده ودر نظر داشته باشيد.

1. مطابق با آيين نامه اجرايي طرحهاي تحقيقاتي روند رسيدگي به طرحهاي پيشنهادي در **دانشكدهها و مراكز تحقيقاتي مصوب** دانشگاه و شوراي گسترش دانشگاه هاي علوم پزشكي مشابه يكديگر بوده و در مورد مراكزي كه وابسته به دانشكده ها ميباشند از طريق دانشكده مربوطه عمل ميشود.
2. **خلاصه روند بررسي طرحهاي** تحقيقاتي بدين ترتيب است ، براين اساس اين فرم بايد پس از تكميل جهت بررسي و طي مراحل تصويب به معاونت پژوهشي دانشكده مورد نظر طرح دهنده تحويل شود.
3. تكميل و ارائه پيش نويس طرح به مرکز تحقيقاتي زير مجموعه و ثبت آن
4. ارائه طرح پيشنهادي به شوراي پژوهشي مرکز مربوطه جهت بررسي و و انجام تغييرات احتمالي در مرکز مربوطه
5. تصويب موضوع در شوراي پژوهشي مرکز
6. ارائه طرح مصوب به شوراي پژوهشي پژوهشکده جهت ادامه روند بررسي
7. تصويب موضوع در شوراي پژوهشي پژوهشکده
8. ارائه طرح مصوب پژوهشکده به حوزه معاونت پژوهشي دانشگاه، در صورتي که اعتبار طرح از حد معيني بالاتر باشد.
9. ثبت در فهرست نوبت شورا و ارجاع به كارشناسان جهت بررسي هاي كلي، در صورت ارائه طرح به دانشگاه.
10. تكميل اطلاعات و قرارگرفتن طرح در دستور كار شوراي پژوهشي دانشگاه، در صورت ارائه طرح به دانشگاه.
11. تصويب موضوع در شوراي پژوهشي دانشگاه، در صورت ارائه طرح به دانشگاه.
12. اعلام به مجري و مقدمات قرارداد …
13. در مواردي كه اجراي طرح پيشنهادي مستلزم **همكاري بخشها يا سازمانهاي ديگر** باشد, طرح دهنده بايد قبلا" نظرموافق سازمانهاي مربوطه را كسب نموده و موافقت نامه كتبي ايشان را ضميمه اين پيش نويس نمايند.
14. كليه طرحهايي كه به تصويب شوراي پژوهشي پژوهشکده ميرسد بر اساس قراردادي كه بين پژوهشکده و مجري طرح منعقد مي‏شود قابل اجرا خواهد بود. بنابراين **پژوهشکده هيچگونه مسئوليتي در برابر فعاليتهاي قبل ازتصويب طرح وآنچه كه خارج ازمحدوده قرارداد منعقده انجام پذيرد نخواهد داشت**.
15. طرح دهندگان ملزم به رعايت كليه **ضوابط و قوانين مندرج در آئين نامه طرحهاي تحقيقاتي پژوهشکده** ميباشند. لذا پيشنهاد ميگردد تا مجريان و طرح دهندگان محترم جهت آگاهي ازمفاد آيين نامه مذكور به دفتر مديريت پژوهشي پژوهشکده يا مراكز تحقيقاتي مراجعه نمايند.
16. چنانچه انجام طرح پژوهشي در مرحله‏اي از پيشرفت آن اعم از اينكه به نتيجه نهايي رسيده يا نرسيده باشد، **منجر به كشف يا اختراع و يا تحصيل حقوقي شود**، مجري طرف قرارداد موظف است مراتب را كتبا" به پژوهشکده اطلاع دهد. در اين رابطه حقوقي كه در اثر اجراي طرح تحقيقاتي ايجاد گرديده است با توجه به متن قرارداد منعقده و يا متمم آن مشخص ميگردد.
17. در صورت تمايل مجري به انتشار يا ارائه نتايج حاصله در داخل يا خارج از كشور ( بجز نشريات دانشگاهي و علمي پژوهشي ), لازمست قبلا" نظر موافق پژوهشکده را در اين زمينه جلب نمايد. بديهي است كه **ذكر حمايت مالي و همكاري پژوهشکده در اجراي طرح براي انتشارات** مذكور الزامي خواهد بود.
18. كليه **تجهيزات و لوازم مصرفي و غيرمصرفي باقيمانده** از اجراي طرح كه از محل اعتبار آن تهيه شده است، پس از اتمام اجراي طرح متعلق به پژوهشکده بوده وهرگونه تصرفي در آنها منوط به كسب مجوزهاي قانوني است.
19. در صورتيكه قراردادي در مورد تجهيزات و لوازم و موادي كه از محل اعتبار پژوهشي تهيه شده است بين پژوهشگر و سازمانهاي ديگر و دانشگاه منعقد شده باشد مطابق اين قرارداد عمل خواهد شد.
20. در صورتيكه هريك از بندهاي 2و3و4 و 12و … رعايت نشود بررسي طرح ممكن نبوده و مسئوليتي از اين بابت متوجه شوراهاي پژوهشي گروه دانشكده و دانشگاه نمي باشد.

**راهنماي تكميل اين فرم**

1. اين فرم بايد به زبان فارسي (ودرصورت لزوم انگليسي) تايپ شده و فاقد هرگونه ابهامي، تكميل گردد. بنابر اين **مديريت پژوهشي پژوهشکده ميتواند از پذيرفتن فرمهايي كه به نحو نامطلوب تكميل شده است، خودداري نمايند.**
2. كليه قسمتهاي فرم بايد به نحو مناسب تكميل شده و طرح دهنده بايد به كليه نكات وتذكرات متن فرم توجه كامل داشته باشند تا**هيچ موردي بي جواب و بدون علامت و توضيح نباشد**.
3. در پاره اي از موارد كه فضاي كافي براي توضيحات مد نظر طرح دهنده وجود ندارد، وي ميتواند **توضيحات اضافه را در برگه أي بصورت ضميمه و با اشاره** به بند و قسمت مورد نظر به فرم اضافه نمايند. چنانچه طرح دهنده از شكل رايانه اي اين فرم استفاده مينمايد هيچ محدوديتي در فضاهاي پيش بيني شده وجود ندارد.
4. براي ارائه فرم رايانه اي طرح ، اين فرم بايد در قالب نرم افزار95 Microsoft Word و يا ويرايش هاي بالاتر از آن باشد.
5. هنگام ارائه اين فرم تكميل صفحه **خلاصه مشخـصات طرح الزامي است.**
6. هنگام ارائه اين فرم **جدول همكاران اصلي طرح** (بند 12) **بايد تكميل شده و به امضا و تاييد فرد مورد اشاره رسيده** باشد.
7. لازم است طرح دهنده **براي تكميل قسمت روش اجراي طرح** به جدول نوع تحقيق ( بند25 و 26) توجه كامل داشته و **كليه موارد اشاره شده** در بند مربوطه را توضيح دهد.
8. لازم است طرح دهنده توضيح كاملي در رابطه با ابزار جمع آوري اطلاعات (پرسشنامه و يا …) ارائه نموده و نمونه اي از آن را ضميمه نمايد.
9. در صورتيكه ملا حظات اخلاقي براي اجراي طرح وجود دارد لازم است طرح دهنده توضيح كاملي در باره اين نكات ارائه نموده و نمونه اي از رضايت نامه مورد استفاده براي طرح را ضميمه نمايد.
10. در صورتيكه محدوديتهايي براي اجراي طرح تصور ميشود لازم است طرح دهنده به اين محدوديتها اشاره نموده و توضيح كاملي براي مقابله با اين محدوديتها ارائه نمايد.
11. زمان شروع طرح، بعد از تصويب آن بوسيله شوراي پژوهشي پژوهشکده و با هماهنگي مدير اجرايي طرح و حوزه مديريت امور پژوهشي، از هنگام تامين اعتبار در نظر گرفته ميشود
12. دريافت گزارشهاي علمي و اجرايي طرح با توجه به جدول گانت طرح مصوب صورت ميپذيرد. براين اساس لازم است طرح دهنده در جدول مذكور زمان ارائه گزارشهاي پيشرفت طرح و محتواي پيش بيني شده گزارش خود را مشخص نمايد.
13. هزينه هاي كارمندي (پرسنلي) با توجه به **حجم فعاليت** (در نظر گرفتن ساعات كار براي افراد شاغل در طرح) و **جدول زمان بندي** اجراي طرح و **پيوست شماره يك** (كه فعلا مد نظر قرار دارد) تكميل ميشود.
14. طرح دهنده بايد توجه داشته باشد كه حد اكثر ساعات كار براي افراد شاغل در طرح مطابق قوانين اداري واستخدامي 64 ساعت در هر ماه است. رقم حق الزحمه ساعتي افراد با توجه به موقعيت استخدامي و رتبه علمي، در پيوست شماره يك آمده است. بنابر اين طرح دهنده ميتواند با تغيير در ساعات كار رقم كلي حق الزحمه افراد همكار را تغيير دهد
15. **هرگونه نقص يا اشتباهي درمحاسبه هزينه هاي** پيش نويس كه در تصميمات متخذه دخالت داشته باشد به **عهده تكميل كننده فرم است** و دانشگاه تعهدي در خصوص تامين كسري موارد اشتباه شده ندارد.
16. در موارديكه مجري محترم در تكميل اين فرم نيازمند **راهنمايي** باشد ، اين حوزه با هماهنگي قبلي به **نشاني انتهاي اين صفحه** در خصوص راهنمايي‏هاي مورد نظرآمادگي كامل دارد.

### توضيح مفاهيم

| شماره | كلمه | مفهوم |
| --- | --- | --- |
| 1 | طرح دهندگان | فرد يا افرادي هستند كه پيشنويس طرح را تهيه نموده و معمولا اجراي تحقيق نيز بوسيله ايشان انجام ميپذيرد. بر اين اساس و با توجه به آيين نامه طرحهاي تحقيقاتي در اين نوشتار عبارات مجريان يا مجريان اصلي و طرح دهندگان معادل يكديگرهستند. |
| 2 | مدير اجرايي طرح | فردي است كه ازبين مجريان طرح انتخاب شده ومسوليت اجراي طرح از نظر مالي ، حقوقي واداري بعهده ايشان است. بين مدير اجراي طرح وسايرمجريان امتيازخاصي وجود ندارد و مديراجراي طرح صرفا مسئول اجرايي وطرف مذاكره و عامل اجراي طرح شناخته ميشود.. يك طرح تحقيقاتي نميتواند بيش از يك مديراجرايي داشته باشد. |
| 3 | همكاران اصلي طرح | همكاراني هستند كه حضور شخص يا همكاري تخصص ايشان در انجام طرح ضروريست |
| 4 | طرح كاربردي | طرحي است كه داراي نتايج بالفعل بوده و نتايج حاصل از انجام آن بلا فاصله پس از اتمام طرح قابل استفاده باشد |
| 5 | طرح بنيادي | طرحي است كه داراي نتايج بالقوه بوده و نتايج حاصل از انجام آن بلا فاصله پس از اتمام طرح قابل استفاده نباشد |
| 6 | طرح جامعه نگر | طرحي است كه بر اساس نياز بهداشتي درماني جامعه ، در تلاش براي رفع مشكل ويا يافتن پاسخ سوال مشخصي باشد |
| 7 | HSR | Health System Research تحقييقاتي را شامل ميشود كه در قالب طرحهاي جامعه نگر ارائه ميشوند |
| 8 | هدف اصلي طرح | general objective هدفي است كه طرح در انتها بدنبال دستيابي به آن است. اين هدف بايد با انجام طرح قابل حصول باشد |
| 9 | اهداف فرعي طرح | Specific objectives اهدفي هستند كه قبل يا همگام با هدف اصلي طرح حاصل خواهند شد. |
| 10 | اهدف كاربردي طرح | Applied objectives اهدافي هستند كه بصورت عملي پس از انجام طرح بدست آمده و جزو نتايج بالفعل طرح ميباشند. |
| 11 | فرضيات | Hypothesis انتظارات و پيشفرضهايي است كه طرح دهنده بر اساس آن اقدام به تنظيم پيش نويس طرح نموده است. |
| 12 | ملاحظات اخلاقي | Ethical points Or Ethics Considerations كليه اصول و مباني اخلاقي، انساني، مذهبي، و… است كه بايد در حين پژوهش از سوي آزمايشگر بر روي آزمودني اعم از انسان و حيوان و … رعايت شود |
| 13 | جدول زمان بندي مراحل اجراي طرح | Gantt Chart جدولي است كه طي آن محقق ابتدا و انتهاي فعاليتهاي اجرايي و طول زمان مراحل مختلف پژوهش را در آن مشخص ميكند. |

**قسمت دوم - خلاصه مشخـصات طرح**

| **عنوان**: نقش تعدیل­کننده سبکهای مقابله­ و حمایت اجتماعی در تأثیر درمان گروهی پذیرش و تعهد بر هموگلوبین گلیکوزیله، کیفیت زندگی و خودمراقبتی در بیماران مبتلا به دیابت نوع II |
| --- |
| **Title:** **The Moderating role of coping style and social support in effect of ACT on HbA1c, quality of life and self care in type II diabetics** |

مدير اجرايي طرح^2^ : دکتر زینب شایقیان و دکتر پریسا امیری همکاران اصلي: دکتر کبری روحی گیلانی و دکتر مریم وفایی

دانشـكده / مركز تحقيقاتي : **پژوهشکده علوم غدد درون ریز و متابولیسم**

گـروه : **مرکز تحقیقات پیشگیری و درمان بیماری­های متابولیک**

محيط پژوهش : **بیمارستان لبافی نژاد** مدت اجرا : **24 ماه**

خلاصه ضرورت اجرا و اهداف كاربردي طرح :

مشکلات مزمن سلامت، بیماریهایی هستند که قابل درمان نیستند، بلکه باید توسط بیمار و متخصص سلامت کنترل شوند. به رغم موانع متعدد و مواجه با مشکلات در این فرایند، دانشمندان از درمان این بیماریها ناامید نشده و به طور مستمر برای کنترل این بیماریها در تلاشند. دیابت، به عنوان یک بیماری کشندۀ خاموش شناخته شده است که سالانه مسئول مرگ 4 میلیون نفر در جهان به شمار می­رود از میان بیماریهای مزمن، دیابت، شایع­ترین بیماری متابولیک است که منجر به بروز عوارضی چون بیماریهای قلبی، رتینوپاتی، نوروپاتی، نفروپاتی، کاتاراکت و غیره می­باشد. پیشگیری از این عوارض نیازمند توجه به عوامل متابولیک، روانی، اجتماعی و فردی به منظور خودمراقبتی بیمار است و مراقبت موفق دیابت، نیازمند توانایی در هدف­گذاری و تصمیم­گیری مؤثر است که متناسب با الگوی زندگی بیماران باشد. خودمراقبتی موفق نیز با سطح پایین هموگلوبین گلیکوزیله (HbA1c) همراه است که نشاندهندۀ احتمال کاهش عوارضِ در حال پیشرفت دیابت است. از سوی دیگر، استرسهاي مزمن و روزمره در شیوع دیابت، اختلال­های روانشناختي، مثل افسردگی و بيماريهاي جسمي، مثل بیماریهای عروق قلبی نقش دارد که به اهمیت فرایند مقابلۀ بیماران اشاره دارد. علاوه براین، حمایت اجتماعی می­تواند تأثیرات سوءِ ناشی از بیماری مزمن را کاهش دهد و به بیماران کمک نماید تا سازگاری بهتری را با بیماری خودشان داشته باشند. گرچه تأثیرات آن بر زنان و مردان متفاوت بوده اما پیامدهای روانشناختی و اجتماعی دارد. در تحقیق حاضر، جهت دستیابی به هدف درمان دیابت که حفظ هموگلوبین گلیکوزیله نزدیک به دامنۀ طبیعی و پیشگیری از عوارض آن است، درمان پذیرش و تعهد به عنوان مداخلۀ مؤثر در نظر گرفته شده است. به این منظور 100 نفر از بیماران مبتلا به دیابت نوع II، بر اساس معیارهای ورودی به طور تصادفی، به دو گروه مداخله و کنترل تقسیم می­گردند. آزمودنی­های گروه آزمایش، علاوه بر دارو درمانی جاری و کارگاه آموزشی یک روزه، در 10 جلسۀ یک بار در هفته درمان پذیرش و تعهد شرکت می­کنند. آزمودنی­های گروه کنترل، دارو درمانی جاری را به علاوه یک کارگاه آموزشی یک روزه دریافت می­کنند. در شروع مطالعۀ آزمودنی­های هر دو گروه پرسشنامه کیفیت زندگی، فرم کوتاه سبک­های مقابله­ای، مقیاس خود مراقبتی دیابت، پرسشنامه حمایت اجتماعی و پرسشنامه پذیرش و عمل دیابت را تکمیل می­کنند و آزمایش هموگلوبین گلیکوزیله (HbA1c) از آنها گرفته می­شود. تمام آزمودنی­ها 3ماه بعد از ورود به مطالعه، مجدداً پرسشنامه­های قبلی را تکمیل خواهند کرد به همراه آزمایش خون مجدد. نهایتاً، تحقیق با تحلیل داده­ها از طریق آزمون تحلیل کوواریانس و اندازه های اثر مکرر اثر درمان تعهد و پذیرش بر بهبود نشانه­های بیماری دیابت ارزیابی خواهد شد. هدف از مطالعۀ حاضر، بررسی اثر درمان پذیرش و تعهد بر بهبود هموگلوبین گلیکوزیله، کیفیت زندگی و خودمراقبتی بیماران مبتلا به دیابت نوع II، با در نظر گرفتن نقش تعدیل­کنندۀ دو متغیر سبک­های مقابله­ای و حمایت اجتماعی می­باشد.

**قسمت سوم – اطلاعات مربوط به طرح پژوهشي**

1. عنوان طرح به فارسي : نقش تعدیل کننده سبکهای مقابله و حمایت اجتماعی در تأثیر درمان گروهی پذیرش و تعهد بر هموگلوبین گلیکوزیله، کیفیت زندگی و خودمراقبتی در بیماران مبتلا به دیابت نوع II

The Moderating role of coping style and social support in effect of ACT on HbA1c, quality of life and self care in type II diabetics

1. نوع طرح :

كاربردي^4^ بنيادي^5^ بنيادي-كاربردي جامعه نگر^6^(HSR) ^7^

**تعريف مسأله، اهداف، و سوالات تحقيق:**

مشکلات مزمن سلامت، بیماریهایی هستند که قابل درمان نیستند، بلکه باید توسط بیمار و متخصص سلامت کنترل شوند. با این حال بشر از درمان این بیماریها ناامید نشده و و برای یافتن راه بهبود آنها در تکاپوست. اما درمان بیماریهای مزمن، مثل سکته مغزی، سرطان، دیابت و بیماریهای قلبی هزینۀ بالایی را بر دوش دولت­ها گذاشته است. به علاوه 60% از مرگ و میرهای جهان ناشی از این بیماریها می­باشد (پریودینی، پریستی، رابیتی، میسلی و مدراتو، 2011). هر بیماری مزمن، مثل سکتۀ مغزی، سرطان، بیماریهای قلبی و تنفسی مزمن، دیابت، آرتروز و مانند اینها می­توانند دهه­ها به طول انجامد و اغلب ریشه­اش به سنین جوانی برمی­گردد (پریودینی و همکاران، 2011). زمان طولانی که برای این بیماریها سپری می­گردد ممکن است تهدیدی برای پیشگیری از علائم یا بدتر شدن بیماری یا کیفیت زندگی بیماران باشد. شرایط محیطی و ویژگیهای فردی هر دو از لحاظ سبک زندگی و عوامل روانشناختی، می­توانند اثر بیماری بر کیفیت زندگی افراد را تعدیل کند (پینکاس، بورتن، ووگن و فیلد، 2002). بنا به اظهارات کرتیس (2000)، همۀ این بیماریها، عوامل روانشناختی دارند که در هنگام شروع، کنترل و درمان آنها دخالت می­کنند. از آنجائیکه این بیماری­های جدید زندگی، از نظر ماهیتی چندعاملی هستند، روانشناسی نیز مانند پزشکی، نقش مهمی در پیشگیری، کنترل و درمان آنها ایفا می­کند.

از میان بیماریهای مزمن، دیابت، شایع­ترین بیماری متابولیک با شیوعی رو به افزایش، ازیاد 122درصدی جمعیت مبتلایان طی سال­های 2025- 1995، ایجاد هزینه­های مستقیم (15% - 5/2% کل بودجه بهداشتی) و غیر مستقیم و پنهان، پدیدآورنده­ی عوارضی چون انواع بیماریهای قلبی، رتینوپاتی^[[1]](#footnote-1)^، نوروپاتی^[[2]](#footnote-2)^، نفروپاتی^[[3]](#footnote-3)^، کاتاراکت و غیره^[[4]](#footnote-4)^، مسئول چهار میلیون مرگ در سال، 9% کل مرگ­های جهان، شیوعی برابر با 2/7% در جمعیت بالای 30 سال تهران، وجود حداقل 2 میلیون مبتلا در ایران که در نیمی از موارد از بیماری خود بی اطلاعند، می­باشد (دلاوری و همکاران، 1383). حدود 5/22%- 5/14% از افراد بالای30 سال، عدم تحمل گلوکز^[[5]](#footnote-5)^ دارند که حدود یک چهارم آنها در آینده دچار دیابت آشکار خواهند شد (مرکز تحقیقات غدد ایران، 2002). با توجه به این که 50 درصد دیابتی­ها از دیابت خود اطلاع ندارند و از طرف دیگر سن ابتلا به دیابت در ایران 10 تا 15 سال کمتر از استاندارد جهانی است (نخعی، 1387). پس انتظار می­رود که شیوع واقعی دیابت در ایران بیش از رقم­های گزارش شده باشد. به طور کلی دیابت در بزرگسالان در موارد بسیاری تشخیص داده نشده و مورد درمان قرار نمی­گیرد (اسنوک، 2005).

ديابت از گروه بيماري­هاي متابوليك و يك اختلال چند­عاملي است كه با افزايش قند خون يا هيپرگليسمي^[[6]](#footnote-6)^ مشخص مي­شود و ناشي از اختلال ترشح و يا عمل انسولين و يا هر دو آنها است (فاور، 2008) و به عنوان یک بیماری کشندۀ خاموش از آن نام برده شده است (دلاوری و همکاران، 2003). تقریباً 90 درصد بزرگسالان دیابتی، مبتلا به دیابت نوع II هستند (اسنوک، 2005). دیابت نوع II، بیماری است که به شدت با سبک زندگی مرتبط است و مؤلفه­های رفتاری و هیجانی بسیار قوی دارد و عوارض آن به طور مشخص بر کیفیت زندگی بیماران تأثیر دارد (اسنوک و اسکینر، 2006). در همه جوامع میزان شیوع دیابت نوع II با افزایش سن، بیشتر می­شود و این بیماری در مردان شایع­تر است و با کاهش امید به زندگی به میزان متوسط 10 سال همراه است و از طرفی با گذر زمان اثرات درازمدت دیابت بر دستگاه قلب و عروق، کلیه­ها، شبکیۀ چشم و دستگاه عصبی محیطی، با افزایش قابل توجه در شیوع و مرگ­و­میر همراه خواهد بود (ساکو، 2004).

گرچه تابلوهای بالینی بسیاری از عوارض دیابت شامل بیماری کلیوی برگشت­ناپذیر، نابینایی، انفارکتوس میوکارد^[[7]](#footnote-7)^، بیماری­های عروق کرونری^[[8]](#footnote-8)^ (CDH) و محیطی^[[9]](#footnote-9)^ (PVD) و مغزی^[[10]](#footnote-10)^ (CVD) (یاداو، تیواری و داناراج، 2008) با افزایش سن در غیاب دیابت هم دیده می­شوند، اما همبودی این دو رخداد سبب تشدید همه این عوارض می­شود (اسنوک، 2005). دیابت نوع II اغلب با سایر عوامل خطرساز آرترواسکلروز^[[11]](#footnote-11)^ مثل چاقی، فشار خون بالا و چربی خون بالا همراه است (دی­کاستر و کامینگ، 2004). مجموعه این عوامل توسط ریون (1988)، تحت عنوان "سندرم X "^[[12]](#footnote-12)^ نامیده شده­اند. با وجود عوارض خطرناک دیابت مسئلۀ پیشگیری بسیار با اهمیت می­گردد اما به دلایل متعدد، سازوکارهای پیشگیری اولیه تأثیر چندانی در کاهش میزان بروز دیابت در ایران نداشتند و افراد عموماً، بعد از ابتلا به دیابت، اقدام به پیگیری فعالیت­های درمانی می­کنند (عزیزی، گویا، وزیریان، دولتشاهی و حبیبیان، 2003).

اولین گام ضروری توانمندسازی بیمار، آموزش خودمراقبتی^[[13]](#footnote-13)^ دیابت است که بدون این آموزش، بیماران نمی­توانند به طور آگاهانه، مراقبت بر خود داشته باشند. توانمندسازی در آموزش خودمراقبتی دیابت، به دنبال افزایش دانش، مهارت مراقبت خود، خودآگاهی و احساس استقلال فردی است تا بیماران را در پذیرش مراقبت فردی از دیابت توانا سازد (فانل و اندرسون، 2004). مراقبت موفق دیابت، نیازمند توانایی در هدف­گذاری و تصمیم­گیری مؤثر است که متناسب با الگوی زندگی بیماران باشد و بسیاری از عوامل متابولیک، روانی، اجتماعی و فردی در آن مد نظر گرفته شود (فانل و اندرسون، 2004). روش­های مداخله­ای که بیماران را قادر سازد تا تصمیمات آگاهانه در مورد اهداف، روشهای درمان و رفتارهای مراقبت از خود گرفته و برای مدیریت روزانۀ دیابت برای خود احساس مسئولیت کنند، در کمک کردن به بیماران برای مراقبت از خود مؤثر هستند.

در دیابت نوع II، مشکل عدم وجود علائم در مرحلۀ اولیه در برخی بیماران، منجر به جدی نگرفتن بیماری و عوارض آن می­گردد. به علاوه، شیوع اختلالات روانپزشکی به ویژه اضطراب و افسردگی در افراد مبتلا به دیابت بزرگسالان مانند سایر افراد مبتلا به بیماری­های طبی مزمن نسبتاً بالاست (اندرسون، فریدلند، کلوز و لاستمن، 2001). در بزرگسالان مبتلا به دیابت، افسردگی موجب افت احساس خوشایند جسمی و هیجانی و قطع روش معمول مراقبت از خود (دی­کروت، اندرسون، فریدلند، کلوز و لاستمن، 2001)، همکاری کمتر در پیروی از رژیم غذایی و میزان بالاتر HbA1c، عوارض بیشتر و هزینه­های بالاتر مراقبت از سلامت است (پیروت^[[14]](#footnote-14)^، رابین و سمینریو، 2002). درحالیکه خودمراقبتی در دیابت نوع II نیازمند توجه بیمار به رژیم غذایی، فعالیت بدنی، نظارت بر هموگلوبین گلیکوزیله و انطباق با داروهای تجویز شدۀ دیابت است. خودمراقبتی موفق با سطح پایین هموگلوبین گلیکوزیله (HbA1C) همراه است که نشاندهندۀ احتمال کاهش عوارضِ در حال پیشرفت دیابت است (گریک و همکاران، 2007). اما به طور کل، آموزش صِرف خودمراقبتی دیابت، برای بهبود پیامدهای روانی، اجتماعی و متابولیک، فقط در مدتِ کوتاه مؤثر بوده است (نوریس و همکاران، 2001 و 2002).

علاوه بر این، خودمراقبتی در دیابت، مستلزم مقابله با فشارهای روانشناختی است که می­تواند میزان هموگلوبین گلیکوزیله را پایین نگه دارد و دیابت را کنترل کند (رابین و ناپورا، 2001). استرسهاي مزمن و روزمره که هر دو در بيماريهاي جسمي و ناراحتيهاي روانشناختي نقش مهمي دارند، دیابت را به شدت تحت تأثیر خود قرار می­دهند و از بین بیماریهای مزمن، ديابت نسبت به اثرات استرس كاملاً حساس است (تیلور، 2003). قند خون بالا و پایین بر استرس زندگی افراد مبتلا به دیابت می­افزاید. قند خون بالا، منجر به کاهش سطح انرژی (لین^[[15]](#footnote-15)^، کاتن و ووکورف، 2004) و عملکرد ضعیف­تر شناختی (سامرفیلد^[[16]](#footnote-16)^، دیری و فریر، 2004) می­گردد که عمدتاً با میزان بالاتر آشفتگی هیجانی و کیفیت پایین­تر زندگی همراه است. در مقابل، قند خون پایین­تر از حد تعادل نیز با آشفتگی همراه است (کاکس^[[17]](#footnote-17)^، گاندر-فردریک و مک­کال، 2002). اثرات حاد قند خون پایین از آشفتگی گذرا تا ناراحتی متغیر است و بر رفتار بیمار اثر می­گذارد. به علاوه برخی بیماران از کاهش قند خون بسیار می­ترسند و برخی عمدتاً قند خون را به اندازه کافی بالا نگه می­دارند تا احتمال قند خون پایین را غیر­محتمل سازند اما به صورت چشمگیری خطر عوارض دیابت را افزایش می­دهند (رابین، 2002). و این خود کنترلیهای ناآگاهانه ممکن است به جای پیشگیری از عوارض منجر به تشدید عوارض گردد، بنابراین، توجه به آموزش و مداخلۀ مناسب بسیار مهم و ضروری است. ابتلاي به بيماري ديابت و الزام بيمار به مراقبت­هاي خاص از خويش موجب چالش­هاي فراواني در زندگي روزمره مي­شود كه استفاده از رفتارهاي مقابله­اي را به منظور سازگاري ضروري مي­سازد (گافول و واندل، 2006).

استرس حاد، سبب افزایش ضربان قلب، پاسخ گالوانیکی پوست، انقباض سیستم عروق محیطی، افزایش سطح فعالیت ماهیچه­های اسکلتی، افزایش تولید هورمون­های هیپوفیزی، کتکولامین­ها و توقف ترشح انسولین می­گردد و این کارکردها خود منجر به بالا نگهداشتن سطح هموگلوبین گلیکوزیله می­شود و استرس در افراد دیابتیک به عنوان محرک هایپرگلایسمی^[[18]](#footnote-18)^ در نظر گرفته می­شود (لاستمن و جفری، 2012). علاوه بر اثرات مستقیمی که به آن اشاره شد، اثر غیر مستقیم استرس بر قطع روش معمول مراقبت از خود است (رابین و پیروت، 2001). فرد ديابتي ممكن است به علت استرس، مراقبت در خصوص استفاده از بعضي غذاها را از ياد ببرد يا مصرف داروي خود را فراموش كند كه نتيجۀ آن تأثير بر ميزان هموگلوبین گلیکوزیله­اش خواهد بود (سیبل، 2010). از طرفی شيوه­هاي مقابله­ با استرس مي­توانند نقش مهمي در سير، كنترل، درمان و سازگاري رواني- اجتماعي بيمار با ديابت داشته باشند (تونکی، مسابک، انجین گوک و کَتلا، 2008). مقابله با استرس و به کارگیری سبک­های مقابله­ای اجتناب^[[19]](#footnote-19)^ و انکار^[[20]](#footnote-20)^، ممکن است اثر متضادی بر کنترل دیابت و پذیرش قوانین درمان داشته باشند (لاستمن و جفری، 2012). نتايج پژوهشي بر روي 123 بيمار ديابتي غير وابسته به انسولين، نشان داد كه بيشتر بيماران ديابتي از سبك انطباقي اجتنابي استفاده مي­كنند كه خود منجر به كيفيت نامناسب زندگي مي­شود (کلهو، اموریم و پراتا، 2003). و همین اثرات منفی که دیابت بر سلامت عمومي و احساس خوب بودن فرد دارد بر كيفيت زندگي­اش اثر می­گذارد (لاستمن، اندرسن، فریدلند، دی­گروت و کارنی، 2000). در پژوهش انجام شده توسط گري (2000) مشخص شد كه در بيماران ديابتي عامل استرس­زا معمولاً فرآيندي مزمن است كه طي آن شخص مجبور مي­شود با آن انطباق يابد و اين انطباق مستلزم متابوليسم ويژه و پيامدهاي رواني - اجتماعي خواهد بود. بنابراین در افراد داراي کنترل ضعيف و مشکلات مقابله، تا زماني که مسايل روانشناختي مد نظر قرار نگیرند، آموزش و ابزارهاي مدرن مديريتِ ديابت مؤثر نخواهد بود (ون­در­ون، لباچ، هوگنلست، ون ایپرن، ترومپ- وور، وریند و همکاران، 2005).

همچنین دیابت یک بیماری خانوادگی است، زیرا بر کسانی که فرد مبتلا به دیابت را دوست دارند، با او زندگی می­کنند و یا از او مراقبت می­کنند، تأثیر زیادی می­گذارد و چگونگی پاسخدهی همه این افراد بر چگونگی احساس فرد دیابتی در برقراری رابطه با دیگران و بر چگونگی مراقبت او از خودش تأثیر می­گذارد. بیمارانی که احساس عدم حمایت یا کشمکش می­کنند، دیابت را یک منبع اصلی آزردگی می­دانند (اسنوک و اسکینر، 2006). شفر و همكاران (1986) در مطالعۀ اثر رفتارهای حمایت­کننده خانواده بر افراد ديابتي، نشان دادند که افرادی كه ارتباط ضعيف­تر و نامناسب­تر با خانواده داشتند، كمتر از رژيم ديابت تبعيت می­کردند و در نتيجه از كنترل متابوليك نامطلوبتري نیز برخوردار بودند. هم حمايت اجتماعی عمومي و هم حمايت مربوط به ديابت با تبعيت از رفتارهاي خودمراقبتي در بيماران ديابتي همبستگي دارد و حمايت اجتماعي، به عنوان عامل تسهيل­كننده رفتار بهداشتي شناخته شده است (گیلبرند و استیوسون، 2006). البته برخی تحقیقات به اثر متفاوت حمایت اجتماعی بر زنان و مردان پرداخته­اند، کاپلان، شرلی و هارتول (1987) درتحقیق خود با هدف بررسی اثر حمایت اجتماعی در زنان و مردان مبتلا به دیابت نوع II، 32 مرد و 44 زن را مورد بررسی قرار دادند و نشان دادند که کنترل هموگلوبین گلیکوزیله با رضایت از حمایت اجتماعی در زنان رابطۀ مثبت و معناداری داشت درحالیکه در مردان این رابطه منفی و معنادار بود. هیتزمن و کاپلان (1984)، در مطالعه­ای با هدف بررسی حمایت اجتماعی و جنسیت در افراد مبتلا به دیابت نوع II به نتایج مشابه و همسو با مطالعۀ قبل رسیدند

به طور کل، حمایت اجتماعی می­تواند تأثیرات سوء ناشی از بیماری مزمن را کاهش دهد و به بیماران کمک نماید تا سازگاری بهتری را با بیماری خودشان داشته باشند و افرادی که از حمایت اجتماعی بالایی برخوردارند، بهتر قادر خواهند بود با رخداد­های استرس­زای زندگی سازگاری کنند. همچنین حمایت اجتماعی باعث کاهش آثار منفی استرس­های فراوانی که از محیط اجتماعی کسب می­شود، می­گردد و به تبع آن بر کیفیت زندگی اثر مستقیم و مثبت خواهد داشت (کالیگان و موریسی، 1993). وانگ، وو و لی (2003)، با روش فراتحلیلی 182 پژوهش را در این زمینه بررسی کرده و دریافتند که حمایت اجتماعی، پیش­بین­کنندۀ قوی سلامت در زندگی است. ون، شپرد و پارکمن (2004) نیز با بررسی اثر حمایت اجتماعی بر افراد دیابتی بیان داشتند که دریافت حمایت اجتماعی خصوصاً از جانب خانواده، میزان بهبودی و سازگاری بیماری­های مزمنی مانند دیابت را تسهیل کرده و سبب ارتقای رفتارهای خودمراقبتی و در نتیجه موجب رعایت بهتر رژیم درمانی و کنترل مناسب هموگلوبین گلیکوزیله شود.

حمایت اجتماعی در حکم تعدیل­کنندۀ استرس­های زندگی عمل ­می­کند و سه بعد حمایت اجتماعی عبارتند از: حمایت هیجانی (صمیمیت و قوت قلب دادن) که از خانواده، دوستان، همسر و گروه­های اجتماعی فراهم می­گردد؛ حمایت ملموس (در اختیار گذاشتن کمک­ها و خدمات) که شامل وسایلی ضروری مثل لباس، غذا و سرپناه است و حمایت اطلاعاتی (نصیحت و بازخورد) که شامل اطلاعات و توصیه­هایی است در مورد شیوۀ مراقبت از خود و داشتن فردی همدل که به حرفمان گوش دهد (فرانکن، 2003 ص 529). راتوس معتقد است که حمایت اجتماعی اثرات نامطلوب فشار روانی را از پنج طریق تعدیل و تضعیف می­کند: توجه عاطفی (شامل گوش دادن به مشکلات افراد و ابراز احساسات همدلی، مراقبت، فهم و قوت قلب دادن)، یاری­رسانی (ارائه حمایت و یاری که به رفتار انطباقی می­انجامد)، اطلاعات (ارائه راهنمایی و توصیه جهت افزایش توانایی مقابله­ای افراد)، ارزیابی (ارائه پسخوراند از سوی دیگران در زمینه کیفیت عملکرد منجر به تصحیح عملکرد)، جامعه­پذیری (دریافت حمایت اجتماعی از این طریق ایجاد می­گردد) و در نتیجه اثرات سودمندی به دنبال می­آورد (راتوس، 1990).

با وجود اهمیت این مسئله اما تا همین اواخر، تحقیقات به دورنمای بیماران و توانمندی مراقبت از دیابت، توجه کافی را نداشتند (هیرچ، 2003) حتی به نقش شکل­های خانوادگی و دیگر فرم­های حمایت اجتماعی در مورد دیابت توجه اندکی شده است (فیشر و بارتز، 1998). مطالعات فراوانی پیامد مداخلات مختلف و شیوه­های متنوع و رو به رشد درمانی را ارزیابی کرده­اند و بر اهمیت تشخیص و استفاده از راهبردهای درمانی مؤثر در بزرگسالان مبتلا به این بیماری مخرب، تأکید ورزیده­اند (اسنوک و اسکینر، 2005). هدف درمان دیابت، حفظ هموگلوبین گلیکوزیله نزدیک به دامنۀ طبیعی (130-70 و کمتر از 7%) است که برای پیشگیری از عوارض کوتاه­مدت و بلند­مدت ضروری است (سیبلدس، گادک و چدس، 2006). کنترل میزان هموگلوبین گلیکوزیله در این بیماران نیز تحت تأثیر عوامل مختلفی از قبیل عوامل زیستی، روانی و اجتماعی می­باشد (شاو، گلنت، جکوم و اسپوکن، 2006). از طرفی این بیماران به طور شایع دچار احساس شکست و ناامیدی به دلیل جدال با بیماری و برنامه­ریزی درمان آن گردیده و احساس خوب بودن روحی و اجتماعی آنان تحت تأثیر قرار می­گیرد و اغلب انگیزۀ کافی مراقبت از خود و کنترل دقیق بیماری را ندارند (پلونسکی، 2002) و با کمال تعجب، شواهد پژوهشی حاکی از آن است که بیش از 50 درصد بیماران دیابتی و دارای فشار خون بالا پذیرای دستورات دارویی خود نیستند (کرتیس، 2000). گرچه سودمندي انجام فعاليت بدني در كنترل و مديريت ديابت نوع IIبه خوبي اثبات شده است، اما مطالعات متعدد نشان داده­اند اکثر افراد ديابتي از فعاليت بدني كافي نیز بهره نمی­برند (بلتر، 2001). كاهش فعاليت بدني، نیز باعث افزايش مقاومت به انسولين به عنوان يك فاكتور مشخص پيشرفت ديابت نوع II محسوب مي­شود (یو، وانگ، لی و کیم، 2003). و از آنجا که مراقبت دیابت نیز بر تمام جنبه­های زندگی شخص اعم از تغذیه، ورزش، شغل، تفریح و زندگی خانوادگی و اجتماعی تأثیر می­گذارد (رابین،2000). و تحقیقات بسیاری موانع محیطی و فردی مراقبت مطلوب را معرفی کرده­اند که نقش مهمی در بروز رفتارهای بهداشتی دارد (فیشر، 2006 و گاسگو، توبرت و گیلت، 2001). درمان موفق دیابت، نیازمند تحول تعدیل­کننده­های رفتارهای خودمراقبتی و مداخلات روانی- اجتماعی است که خودمراقبتی مؤثر را ترویج دهد و حفظ کند (رابین و ناپورا، 2001). درحالیکه مطالعات مربوط به اثربخشی اکثر درمان­های روانشناختی، با وجود به دست آوردن موفقیت­های کوتاه­مدت، نشان داده­اند که نتایج این درمان­ها همواره به صورت بلندمدت موفق و مطلوب نبوده است (نوریس و همکاران،2002؛ استن­استورن و همکاران، 2003؛ سورویت و همکاران، 2002). بر این اساس، این سوال مطرح می­گردد که چه عواملی ممکن است نتایج درمان پذیرش و تعهد را به عنوان یکی از درمان­های روانشناختی در مورد بیماران مبتلا به دیابت نوع IIتحت تأثیر قرار دهد؟ لذا مسالۀ اصلي پژوهش حاضر، بررسي متغیرهايي است که به طور خاص در بيماران ديابتي، ممکن است در فرايند و نتيجه درمان نقش تعيين کننده، داشته باشند و با توجه به يافته­هاي پيش گفته در مورد نقش تاثيرگذار دو متغير سبک­هاي مقابله و حمايت اجتماعي، پيش­بيني مي­شود که اين متغيرها مي­توانند نقشي تعيين­کننده در نتايج درمان پذیرش و تعهد بر شاخص­هاي سلامت بيماران مبتلا به ديابت نوع II داشته باشند. بررسي نقش تعديل­کننده سبک­هاي مقابله و حمايت اجتماعي بر نتايج درمان پذیرش و تعهد بيماران مبتلا به ديابت نوع II مستلزم اجراي اين مدل درماني به عنوان يک مداخله ترجيحي براي اين دسته از بيماران نيز هست.

بعد از بیان اهمیت و ضرورت مسئله، در بخش بعدی به اهم درمان­هایی که در زمینۀ دیابت نوع II به کار رفته است می­پردازیم و در راستای هدف مورد نظر تحقیق در قسمت بعدی، درمان پذیرش و تعهد و تحقیقات صورت گرفته در کاربرد این درمان بر روی دیابت نوع II، ارائه خواهد شد.

**اهمیت و ضرورت مسئله**

ديابت به عنوان يکی از بيماري­های مزمن، از نظر مديريت بيماري و لزوم خودمديريتي، پيچيده­ترين بيماري محسوب می­گردد (سریدهر و مادهو، 2002). اهمیت پرداختن به این بیماری به دلیل شیوع و عوارض برگشت­ناپذیر آن مثل بیماری کلیوی برگشت­ناپذیر، رتینوپاتی، انفارکتوس میوکارد^[[21]](#footnote-21)^، کاتاراکت و قطع عضو (یاداو، تیواری و داناراج، 2008) می­باشد که آن را به یکی از مهمترین مشکلات بهداشتی-درمانی و اجتماعی- اقتصادی جهان تبدیل کرده است و بر اساس پیش­بینی سازمان بهداشت جهانی انتظار می­رود، جمعیت بیماران دیابتی از 171 میلیون نفر در سال 2000 به 366 میلیون نفر در سال 2030 برسد و هزینه­های کمرشکنی را به بار آورد به طوریکه در آمریکا هزینۀ درمانی بیماران دیابتی در سال 2007 معادل با 174 بیلیون دلار برآورد شده است (انجمن دیابت امریکا، 2008). بنابراین، شیوع فزایندۀ دیابت در دنیا، اداره و درمان دیابت و عوارض آن نیازمند صرف هزینه­های قابل توجهی از جانب بیماران و سیستم بهداشتی- درمانی جامعه می­باشد كه كيفيت زندگي بيماران را به ‌شدت كاهش مي‌دهد (ليو^[[22]](#footnote-22)^ و همكاران، 2010) و اهمیت پرداختن به این مسئله را پر رنگ­تر می­سازد.

علاوه بر اقدامات بسیاری که در زمینه­های پیشگیری و درمانی دیابت صورت گرفته است، تاکنون با شیوع رو به فزونی این بیماری مواجه هستیم (ساکو، 2004) که ضرورت یافتن راهکارهای مؤثرتر درمانی در این زمینه را بیشتر می­کند. بنا به گفتۀ فرانکلین (2008)، 95% مراقبت و درمان دیابت در افراد بزرگسال توسط خود بیمار انجام می­گیرد، لذا برای درمان بیماری دیابت باید ابعاد روانشناختی آن، مد نظر قرار گیرد چرا که فرد مرکز اصلی کنترل و تصمیم­گیری در درمان روزانۀ بیماری خود است. علاوه بر این، مطالعات مختلف تأييد نموده­اند كه با وجود اهمیتِ سازگاري بيمار و مراقبت وي از خويش در نتايج، داشتن اطلاعات به تنهائي كفايت نمي­كند بلكه توانائي بيمار در مقابلۀ مؤثر با استرس­هاي حاصل از ديابت در زندگي روزمره از اهميت ويژه­اي برخوردار است (اسچروس و ریدر، 1997). بنابر این، با توجه به اینکه عواملي به جز خودمراقبتی مي­توانند اثر منفي بر کنترل قند خون داشته باشند، در نظر گرفتن عوامل رواني- اجتماعي مؤثر بر کنترل مناسب قند خون، نقش مهمي در درمان روانشناختي بيماران، افزايش خودمراقبتی، پيشگيري از عوارض بيماري و ارتقاي کيفيت زندگي بيماران دارد. بنابراین طبق مطالعات مطرح شده در بیان مسئله، ضرورت توجه به نقش سبک­های مقابله­ای و حمایت اجتماعی در اثر درمان­های روانشناختی حس می­گردد که اجرای مدل درمانی پذیرش و تعهد همزمان با بررسی نقش تعدیل­کنندۀ سبک­های مقابله­ای و حمایت اجتماعی، می­تواند هم پاسخ سوال اصلی پژوهش را مشخص کند و هم شواهد تجربی لازم برای چگونگی تجدید نظر مدل درمانی پذیرش و تعهد به منظور کارآمدسازی این مدل فراهم نماید.

**مروری بر درمان­های دیابت:**

هدف درمان ديابت، پيش­گيري از عوارض آن به همراه حفظ كيفيت زندگي مطلوب بيماران است (گافول و واندل، 2006). امروزه الگوی غالب مورد استفاده در مراکز بهداشتی و درمانی، مدل پزشکی است. اساس این مدل، تشخیص بیماری­ها، یافتن عامل بیماری­زا، برطرف کردن عامل و درمان بیماری و یا برطرف کردن نشانه­های بیماری است (چین و کارمر، 1999). شاخص مهم جسمی کنترل گلوکز در این بیماران، هموگلوبین گلیکوزیله یا HbA1c است که برای پیشگیری از عوارض دیابت، نیازمند حفظ میزان قند هموگلوبین در پایین­تر از 7% است (هوسیاکس و همکاران، 2010). از مهمترین داروهای تجویز شده برای کاهش هموگلوبین گلیکوزیله عبارتند از: "سولفونیل اوره" این دارو برای افراد ديابتي نوع II كه ديابت آنها قبل از 40 سالگي تشخيص داده شده است، همچنين افرادي كه مدت ابتلا به ديابت در آنها از زمان شروع مصرف دارو كمتر از 5 سال بوده و افرادي كه داراي قند خون ناشتاي كمتر از 300 هستند، بهتر جواب داده است (مرادیان،1996)، "متفورین" از گروه بیگوانیدها می­باشند که اثری در افزایش اثر انسولین ندارد و مقاومت به انسولین را کاهش می­دهد (ملچیور و جابر، 1996). "مهارکننده­های آلفا گلوکوزیداز" دی­ساکاریدازها را مهار می­کنند و از تبدیل کربوهیدرات به گلوکز ساده ممانعت می­کند و سبب می­گردد گلوکز بعد از خوردن غذا افزایش نیابد (کمپل، وایت و کمپل، 1996)، "تیازولیدیندیون­ها" داروهای حساس­كننده­ انسولين هستند که باعث افزايش جذب گلوكز توسط ماهيچه­هاي اسكلتي مي­شود (اسپرانو و سیتن، 1998) و "گلینیدها" باعث تحريك ترشح انسولين مي­شوند (ملیسی، 2003). از جملۀ درمان­های پزشکی برای کنترل دیابت نوع II، که معروفترین آنها نیز می­باشد، درمان با انسولین است. با این حال کنترل عوارض ناشی از تعادل نامناسب هموگلوبین گلیکوزیله با تزریق انسولین و هشیاری دائم نسبت به این قضیه، کار بسیار دشواری است (اکبرزاده، 1384) و بسیاری از افراد به دلیل ترس از تزریق انسولین (ملما، اسنوک، هین و وندر پلوگ، 2001) یا باورهای غلط در مورد انسولین (هاک، امرسون، دنیسون، ناوسا و لویت، 2005) و عوامل روانشناختی دیگر از تزریق انسولین خودداری می­کنند (کابالرو، 2006)

یکی دیگر از روشهای درمان طبی دیابت، پیوند جزایر لانگرهانس پانکراس است که البته استانداردسازی و بهینه نمودن شرایط جداسازی و تلخیص سلول­های جزایر لانگرهانس که از مراحل مهم پیوند است، دشوار و خطرناک است و برای این پیوند باید شرایط لازم وجود داشته باشد (ایبکوکورو، آواچی، یامادا، فوجیموتو، لمینو و اویازر و همکاران، 2002). و با وجود اين كه در سال­هاي اول برخي از بيماران دريافت­كننده­ي جزاير لانگرهانس نياز به انسولين نداشتند، در پيگيري طولاني­مدت ديده شد كه جزاير لانگرهانس پيوند شده فعاليت خود را از دست دادند و بيماران ناچار به استفاده­ي مجدد از انسولين شدند (شاپیرو، ریکوردی، هرینگ، آچینکلوز، لیندبلند، ربرتسون و همکاران، 2006).

مطالعه درمان طبی دیگر، بررسی اثر متابولیک ویتامین D فعال در بیماران دیابتی نوع II است (چایو، چو و ساد، 2004). بنکداران و افخمی­زاده (1389)، در مطالعۀ خود، نشان دادند که با اینکه مصرف فرم فعال ویتامین D در بیماران دیابتی موجب بهبود میزان قند خون ناشتا، هموگلوبین گلیکوزیله و میزان مقاومت به انسولین گردید ولی تنها اثرات درمانی بر کاهش میزان کلسترول LDL و فشار خون دیاستولی پس از درمان مؤثر بود و درمان با کلسیتریول اثر معناداری بر کنترل هموگلوبین گلیکوزیله در بیماران دیابتی نوع II نداشت.

رژيم درماني، بخش مهمي از برنامه­ي درمان بيماران مبتلا به ديابت نوع IIرا تشكيل مي­دهد که در آن کاهش دريافت قندهاي ساده، چربيهاي اشباع، کلسترول، افزايش دريافت ميوه­ها و سبزیجات و فيبرهاي غذايي توصيه مي­شوند و اين مداخلات سبب بهبود سطح ليپيدهاي سرم، تنظيم گلوکز خون، کاهش فشار خون، حفظ يا کاهش وزن بدن و کاهش عوارض ناشي از ديابت مي­شوند (رودریگاس و کاستلانوس، 2000). علی­رغم شواهد فراوان مبني بر تأثير رژيم درماني بر ديابت، تغيير رژيم غذايي و حفظ آن براي بيماران مبتلا به ديابت مشكل است و حتي در بسياري موارد اين بيماران به دليل عدم آگاهي کافي، با اين توصيه­ها مخالفت می­کنند (گلاسگو، هامپسن، استرچر و روگییرو، 1997). در حال حاضر، الگوی مدل پزشکی، پاسخ­گوی بسیاری از نیازها و مشکلات دیابت نیست (چین و کارمر، 1999). علاوه بر آن، بروز مشکلات روحی، هیجانی و اجتماعی مانع انجام و تداوم مراقبت­های پزشکی می­شود (پلونسکی، 2002).

برای درمان این اختلال علاوه بر درمان­های دارویی، درمان­های روانشناختی متعددی نیز در طول سال­های متوالی ابداع شده است و الگوهای مختلفی برای آموزش بیماران و تغییر رفتار آنان استفاده شده است. اهم درمان­های روانشناختی دیابت؛ خودمدیریتی، مدیریت استرس^[[23]](#footnote-23)^ و آرام­سازی^[[24]](#footnote-24)^ و درمان شناختی­رفتاری^[[25]](#footnote-25)^ بوده­اند که به هر کدام از آنها به طور مختصر و خلاصه می­پردازیم.

آموزش خودمدیریتی^[[26]](#footnote-26)^ دیابت، پایه و اساس دیدگاه توانمندسازی است و هدف از آموزش بیماران، کمک به آنها برای گرفتن تصمیماتی در مورد مراقبت از خود و دستیابی به اهداف، ارزشها و انگیزش­هاست (فانل، سیدی­ای و اندرسون، 2004). آموزش خودمدیریتی، به بیماران اطلاعاتی پزشکی راجع به رژیم و ورزش می­دهد و همراه با آموزش تنظیم قند خون است (بودنهیمر، لوریگ، هولمن و گرامبچ، 2002). گرچه این تمرینها اثرات مثبتی بر کنترل دیابت دارند، اما بسیاری بیماران در اجرای این مراقبت­ها قصور می­کنند (رابین و ناپورا، 2001). باستین و همکاران (2009)، به مطالعۀ اثر آموزش خودمدیریتی دیابت پرداختند و برای این منظور برنامۀ آموزش خودمدیریتی را به مدت 3 ماه (هر هفته 2 ساعت)، بر 44 بیمار مبتلا به دیابت نوع II اعمال کردند و نشان دادند که میزان هموگلوبین گلیکوزیله (به میزان 6/0) کاهش یافت و بعد از 12 ماه نیز ادامه داشت، اما کاهش، بعد از 18 ماه پایدار نماند و نتیجه اینکه برای پایداری اثر مداخله لازم است که این روش به صورت طولانی­مدت و دوره­ای ادامه پیدا کند. آموزش­های خودمدیریتی بسیاری برای کنترل و درمان دیابت صورت گرفته­اند، اما مطالعات نوریس و همکاران (2001 و 2002) با ارزیابی آزمایش­هایی که طی سال­های 1980 تا 1999 برروی آموزش خودمدیریتی برای افراد مبتلا به دیابت نوع II صورت گرفته بود، نشان دادند که آموزش صِرف خودمدیرتی دیابت، برای بهبود پیامدهای روانی، اجتماعی و متابولیک، فقط در مدت کوتاه مؤثر بوده است که به گفتۀ آنها عوامل مؤثری ممکن است سبب این نتایج باشند مثل: 1- اثر میانجی عوامل روانشناختی حاکم، 2-ویژگیهای مداخلات مثل ارتباط فرهنگی، 3- عوامل زمینه­ای مثل ساختار سیستم سلامت و وابسته­های مراقبت اولیه. با اینکه نتایج کوتاه مدت معنادار بودند اما ثابت گردیده است که حفظ کنترل و درمان این بيماري مستلزم رفتارهاي خودمراقبتي ويژه در تمام عمر است (گیلبرند و استیونسون، 2006). اسکینر و همکاران (2006) در مطالعه خود 236 بیمار مبتلا به دیابت نوع II را طی 3ماه در برنامه مداخله­ای خودمدیریتی و آموزش دیابت شرکت دادند و نشان دادند که وقتی برنامه مداخله­ای خودمدیریتی با آموزش­های دیابت همراه شد، توانست سبب کنترل هموگلوبین گلیکوزۀ بیماران گردد. ایشان ذکر می­کنند این روش تنها زمانی نتیجه می­دهد که دقیقاً طبق فرمول­بندی نظری به آن عمل گردد. بنابراین روش مذکور هزینه بردار است و طبق گزارش اسکینر و همکاران (2006) نیازمند استفاده از 4 متخصص (1متخصص رژیم دیابت، 2 مشاور پرستار دیابت، یک روانشناس بالینی و یک روانشناس سلامت) است. لذا با امکانات موجود در بیمارستان­های ایران شاید به صرفه و عملی نباشد.

روش دیگر، مدیریت استرس است. آموزش مدیریت استرس معمولاً شامل آرام­سازی ماهیچه­ای پیشرونده همراه با یا بدون پسخوارند زیستی، تصویرسازی ذهنی، تنفس دیافراگمی و آموزش در زمینۀ تغییر پاسخ­های رفتاری، شناختی و فیزیولوژیکی به استرس است (سورویت، توبرت، زوکر، مک­کاسکیل و پارخ، 2002). با توجه به اینکه در برخی مطالعات رابطۀ معناداری بین استرس و میزان قند خون وجود دارد (لاستمن و جفری، 2012؛ رابین و پیروت، 2001؛ ون­در­ون و همکاران، 2005). شاید به نظر برسد که برنامه­ها و مداخلات مدیریت استرس می­توانند در کنترل هموگلوبین گلیکوزیله مؤثر واقع شوند. به این منظور به بررسی برخی از این مطالعات می­پردازیم؛ استن­استورن و همکاران (2003)، با هدف بررسی مدیریت استرس بر کنترل قند خون افراد دیابتیک، برنامۀ 14 هفته­ای (2 ساعت در هفته) مدیریت استرس به همراه تکنیک آرام­سازی را برای دو گروه 31 نفره ترتیب دادند؛ بررسی­های ایشان نشان داد که گروه مداخله، به طور معناداری تغییرات مثبت خلقی را تجربه کردند اما هیچ تفاوت معناداری در میزان هموگلوبین گلیکوزیله رخ نداد و تفاوت معناداری نیز میان تغییرات خلقی و تغییرات قند خون حاصل نشد، ایشان بیان داشتند که طبق یافته­های به دست آمده نمی­توان انتظار داشت که آموزش آرام سازی و مدیریت استرس وقتی به عنوان تنها درمان روانشناختی انجام پذیرد، نتیجه مطلوبی بر کنترل هموگلوبین گلیکوزیله داشته باشد و ایشان پیشنهاد می­کند که بهتر است این درمان­ها به منظور کنترل استرس بیمار، مکمل درمان­های دیگر روانشناختی مد نظر قرار گیرند. سورویت و همکاران (2002)، نیز با نمونۀ 180 نفری بیماران مبتلا به دیابت نوع II (30 سال به بالا) همین مطالعه را انجام دادند و به نتایج مشابه رسیدند و بعد از انجام مداخلۀ مدیریت استرس و پیگیری یک­ ساله، نشان دادند که تفاوت معناداری بین گروه­های آزمایش در میزان کنترل هموگلوبین گلیکوزیله مشاهده نشد. کارلسون و همکاران (2003) اثر تکنیک­های مدیریت استرس را بر روی 63 نفر از افراد مبتلا به دیابت نوع II در سنین 25 تا 70 سال، با هدف کاهش استرس­های همراه دیابت، بررسی کردند و نشان دادند که گروه آزمایش بعد از شش جلسۀ مدیریت استرس، به طور معناداری نسبت به گروه کنترل استرس خود را مدیریت می­کردند و مراقب دیابتشان بودند، اما وقتی تغییرات در پیش­آزمون و پس­آزمون مقایسه شد این یافته­ها حمایت نشدند و مقایسۀ پیش­آزمون و پس­آزمون تفاوت معناداری را بین سبکهای مقابله­ای هیجان­محور و مسئله­محور در مقابل استرس، نشان داد که ممکن است اشاره داشته باشد به اینکه افراد شرکت­کننده در برنامه مداخله پذیرفته­اند که بصورت فعالتر عمل کنند. تاکنون پژوهشهای زیادی در مورد اثر آموزش تکنیک­های مدیریت استرس، مانند آرام­سازی و پسخوراند زیستی برای بیماران دیابتی نوع I و II، صورت گرفته است و برخی مطالعات شواهدی در تأیید اثر این برنامه­های مداخله ارائه داده­اند (سارویت، ونتیلبرگ، زوکر، مک کاسکیل، پارخ، فینگلس و همکاران، 2002 و عطاری، صفریپور، امینی و حقیقت، 2006)، در حالیکه برخی مطالعات دیگر به چنین شواهدی دست نیافتند و نتایج در این زمینه متفاوت است (فینگلوس، هاستد و سارویت، 1987 و جابلن، نابیلف، گیلمور و رزنتا، 1997). درمان شناختی­رفتاری اساساً جهت درمان اختلال­های خلقی بسط یافت (بک، 1988) و به طور موفقیت­آمیزی در گروه­های متفاوت بیماریهای مزمن اعمال شده است (ولاین و مورلی، 2005). باور اصلی درمان شناختی­رفتاری این است که احساسات، شناخت و رفتارهای ناسالم انسان، توسط یادگیری کسب می­گردد و همینطور نیز می­تواند یادگیری­زدایی^[[27]](#footnote-27)^ گردد (کُرِی، 2008 ص 347) و این درمان برای دیابت توسط اسنوک و همکارانش در سال 1999 مطرح شد. هدف درمان شناختی­رفتاری، کمک به بیماران برای تشخیص شناخت­های ناکارآمد، آزمون آنها در مقابل واقعیت و تغییر دادن آنهاست (اسنوک و همکاران، 2001). درمانگران شناختی­رفتاری، با استناد به اینکه افسردگی (اندرسون و همکاران، 2001؛ دی­گروت و همکاران، 2001) و اضطراب (بویل، الن و میلر، 2004) در بیماران دیابتی شایع است و این افسردگی و اضطراب است که مانع انجام فعالیت­های خودمراقبتی دیابت می­گردد (دی­کروت، اندرسون، فریدلند، کلوز و لاستمن، 2001؛ پیروت^[[28]](#footnote-28)^، رابین و سمینریو، 2002)، لذا اکثر درمان­های شناختی­رفتاری بر درمان افسردگی (رابین و پیروت، 2001) و اضطراب (بویل، الن و میلر، 2004) افراد دیابتی انجام گرفته است و یافته­ها نیز گویای تأثیر CBT بر درمان افسردگیِ افراد دیابتی دارد (لاستمن، گریفت، فریدلند، کیسل و کلوز، 1998 به نقل از رابین و پیروت، 2001). درمان شناختی­رفتاری ، به بیماران کمک می­کند تا از الگوهای افکار علیهِ خود^[[29]](#footnote-29)^ و رفتارهای منفی^[[30]](#footnote-30)^ اجتناب کنند. البته گرچه رهایی از افسردگی ممکن است سبب بهبود کنترل قند خون گردد اما افسردگی و کنترل ضعیف قند خون مترادف نیستند و اگرچه کنترل قند خون و افسردگی رابطه دارند، اما بسیاری از افراد که کنترل ضعیفی دارند، افسرده نیستند و بسیاری از افسرده­ها ممکن است به شیوۀ مؤثری، دیابتشان را مدیریت کنند، نتایج در مورد اضطراب هم مانند افسردگی بود (رابین و پیروت، 2001). مداخلات مبتنی بر درمان شناختی­رفتاری، قبلاً به طور موفقیت­آمیزی در تغییر رفتار سالم به کار رفته است (هابیس و ساتن، 2005). اختصاصاً در مورد دیابت نیز طیف گسترده­ای از درمانهای رفتارگرا انجام شده­اند که برخی نتایج امیدوارکننده­ای را نشان داده­اند، اما اثرات بلند­مدت قانع­کننده­ای را ارائه ندادند (ون­در ون، هوگنسلد، ترامپت­وور، توایسک، وندر پولگ و هین، 2005). اسنوک و همکاران (2001)، برای بررسی اثر درمان گروهی شناختی­رفتاری بر بیماران مبتلا به دیابت، 107 بیمار دیابتیک با کنترل پایین قند خون را بررسی کردند و دریافتند که بعد از 6 هفته (هر جلسه 5/1 ساعته)، هموگلوبین گلیکوزیله تنظیم شد، اما تحلیل بیشتر داده­ها نشان داد که بهبود کنترل هموگلوبین گلیکوزیله تنها در بیمارانی که افسردگی شدید داشتند مؤثر بود. علاوه بر این اسماعیل، وینکلی و راب­هسکت (2004)، در مطالعۀ فراتحلیل خود با هدف بررسی اثر درمان شناختی­رفتاری بر بهبود هموگلوبین گلیکوزیله در افراد مبتلا به دیابت نوع دو، 25 مطالعه را در زمینۀ مداخلات روانشناختی بررسی کردند و دریافتند که آشفتگیهای^[[31]](#footnote-31)^ روانشناختی و افسردگی به طور معناداری در گروه مداخلات نسبت به گروه کنترل، کاهش داشت اما تأثیر معنادار پایداری بر کنترل هموگلوبین گلیکوزیله و کاهش وزن مشاهده نشد و ایشان بیان داشتند که درمانهای روانشناختی می­تواند در بهبود برخی از ویژگیهای مشخص دیابت اثرگذار باشد و نیاز به یافتن درمانهای روانشناختی مؤثرتر در این زمینه وجود دارد. ون­در ون و همکاران (2005) با بررسی درمان گروهی شناختی­رفتاری بر 107 بیمار دیابتیک، نشان دادند که درمان شناختی­رفتاری سبب بهبود خودکارآمدی^[[32]](#footnote-32)^، استرس همراه دیابت و خلق طی سه ماه پیگیری شد، اما سبب بهبود کنترل هموگلوبین گلیکوزیله بیماران نشد، درحالیکه فرض می­شد که بهبود در خودکارآمدی آنها به کنترل هموگلوبین گلیکوزیله منجر گردد، اما این نتیجه به دست نیامد و با اینکه به نظر می­رسید بیماران به اهمیت مسئلۀ خودمراقبتی واقف شدند اما به سختی، تن به انجام آنها می­دادند و اعتماد کمی در اعمال رفتارهای مورد نیاز داشتند. همچنین آمسبرگ، اندربرو، ردلینگ، لیسپرس، لینز، اندرسن و جکوبسن (2009)، در مطالعه­ای با هدف بررسی اثر درمانهای شناختی­رفتاری بر دیابت، یک برنامۀ 48 هفته­ای برای 69 بیمار دیابتی ترتیب دادند و نهایتاً دریافتند که بسیاری از این برنامه­ها به قدر کافی پاسخگوی نیازهای بیماران نبودند و فقدان برنامه ساختاریافته جهت تغییر و حفظ رفتار سالم وجود دارد و شاید یکی از علل عدم معناداری یافته­ها این باشد که برخی مطالعات به اندازه کافی بر عناصر خاص رفتار تمرکز نداشتند. رولنیک، میلر و باتلر (2008) نیز بیان داشتند که وقتي براي ميزان پاسخ باليني، از معيار و مقیاس دقيق­تری استفاده شود، تعداد قابل توجهي از بیماران به درمان­هاي معمول از جمله درمان شناختی­رفتاری يا تغيير سبك زندگي پاسخ درماني خوبي نمي­دهند.

با وجود کاربرد درمان­های مختلف، افزايش شيوع ديابت، زنگ خطري از كنترل نامطلوب بیماری است (فیشر، 2006 و گاسگو، توبرت و گیلت، 2001). در سه دهۀ گذشته، با وجود پیشرفت­های قابل توجهی که در روش­های درمانی دیابت صورت گرفته است، اما پیامدهای درمانی بیماران هنوز فاصلۀ بسیاری از سطح مطلوب درمان، در کشورهای در حال توسعه دارند (ماروگسان، شوبانا و سنهالات، 2009) و نیاز به بررسی و مطالعۀ درمانهای مختلف برای یافتن کارآمدترین و با صرفه­ترین درمان دیابت، همچنان حس می­شود. پس از بررسی مطالعات مختلف در زمینۀ مداخلات درمانی دیابت، در پژوهش حاضر، قصد داریم که درمان پذیرش و تعهد را به عنوان یک تحول جدید روان­درمانی، به دلایل زیر برای درمان دیابت نوع II مطرح ­کنیم. اول اینکه چنانچه ذکر گردید، مطالعات فراتحلیل نوریس و همکاران (2001 و 2002) نشان داد که در درمان­های روانشناختی قبلی دیابت به عوامل زمینه­ای (بافتی) توجهی نشده است، درحالیکه درمان پذیرش و تعهد مبتنی بر بافت­شناسی است (هسکر، 2010). دوم؛ تحقیقات لاپالانین و همکاران (2007)، نشان داد که درمان پذیرش و تعهد نسبت به درمان­های دیگر روانشناختی دیابت، هزینۀ کمتری می­طلبد و سوم؛ تحقیقات لاستمن و جفری (2012) نشان دادند که اجتناب از بیماری در بیماران دیابتیک بسیار زیاد است که منطبق بر اصول اولیۀ درمان پذیرش و تعهد است که مشکل اصلی اختلالات را اجتناب تجربی (هیز،2004) می­داند.

**درمان پذیرش و تعهد**^[[33]](#footnote-33)^ **(**ACT**):**

نسل اول رویکردهای رفتاری در تقابل با رویکرد اولیۀ روان تحلیلی بر پایه­­ی دیدگاه­های شرطی کلاسیک و عاملی در دهه­های 1950 و 1960 مطرح شدند. نسل دوم این درمان­ها بر نقش باورها، شناخت­ها، طرحواره­ها و نظام پردازش اطلاعات در ایجاد اختلالات روانی تأکید داشتند و اینکه در روان­درمانی باید با تکنیک­های مختلف به تغییر یا تعدیل این باورهای منفی یا حذف کلی آنها پرداخت. از رویکردهای برجسته­­ی نسل دوم می­توان به شناخت درمانی بک و درمان عقلانی- عاطفی الیس اشاره کرد. امروزه نسل سوم این درمان­ها بیشتر مطرح است که آن­ها را می­توان تحت عنوان کلی "مدل­های مبتنی بر ذهن آگاهی و پذیرش^[[34]](#footnote-34)^" نامید، مانند: شناخت درمانی مبتنی بر ذهن­آگاهی^[[35]](#footnote-35)^MBCT ، کاهش استرس مبتنی بر ذهن­آگاهی^[[36]](#footnote-36)^MBSR ، رفتار درمانی دیالکتیکیDBT ^[[37]](#footnote-37)^ و درمان پذیرش و تعهدACT که هیز از آن به عنوان موج سوم^[[38]](#footnote-38)^ درمان­های شناختی و رفتاری یاد می­کند (هیز، 2004). درمان پذیرش و تعهد، مبتنی بر بافت­شناسی^[[39]](#footnote-39)^ و ترکیب شده در تئوری چهارچوب ارتباط^[[40]](#footnote-40)^ (RFT) است (هسکر، 2010). درمان پذیرش و تعهد همچنین با دیدگاه ذهن­آگاهی شباهت دارد، زیرا سعی می­کند پذیرای تجارب روانشناختی با دیدی باز، عاری از قضاوت و بدون ارزیابی باشد، یعنی تجربۀ رویدادها بدون تلاش برای کنترل، تغییر یا سرکوب کردن آنها (هیز و داکوورث، 2006). گاهی اوقات ACT را خارج از یا متضاد با CBT می­دانند (هافمن و اسموندسن، 2008) اما ACT بخشی از خانواده بزرگ درمانهای شناختی و رفتاری (فورمن و هربرت، 2009) و دیدگاه علم رفتاری زمینه­ای^[[41]](#footnote-41)^ (هیز، لیوین، پلامب-ویلارداگ، ویلات و پیستورلو، 2011) است. گرچه درمان پذیرش و تعهد شباهت­های بسیاری با درمان شناختی­رفتاری دارد، اما تفاوت­های آشکاری نیز با هم دارند. گادیانو (2009) در مطالعه خود به بررسی و بازنگری مطالعات در هر دو درمان پرداخت (برای مثال؛ در ACT 2 مطالعه در زمینه افسردگی، 3تا در اضطراب، 2تا در بیماریهای مزمن پزشکی، 2تا با علائم سایکوتیک، 2تا در اعتیاد، 1 مطالعه در درد مزمن و 1مطالعه در اختلال شخصیت مرزی و در CBT 2 مطالعه در افسردگی و 11 مطالعه در اختلال اضطراب، بررسی شدند) و بیان داشت که 38/0 از مطالعات ACT نتایج متفاوتی از CBT به دست آوردند؛ در حقیقت بیشتر تحقیقات ACT، اعمال مداخلۀ درمانی بر مشکلات مقاوم­تر را نسبت به CBT در بردارد مطالعات CBT 5/4 برابر ACT هزینه بردارند که ممکن است این عوامل ناشی از دشواری روش­شناختی میان این دو مداخله باشد. ACT سبب بهبود انعطاف­پذیری شناختی می­شود اما CBT سبب بهبود اعتماد به نفس (لاپالانین، لتونن، اسکارپ، توبرت، اجانن و هیز، 2007)

طبق دیدگاه شناختی­رفتاری، به بیماران کمک می­کند تا از الگوهای افکار علیهِ خود^[[42]](#footnote-42)^ و رفتارهای منفی^[[43]](#footnote-43)^ اجتناب کنند (رابین و پیروت، 2001). درحالیکه طبق دیدگاه ACT یکی از مشکلات اصلی اختلالات، اجتناب تجربی^[[44]](#footnote-44)^(فعالیتی به منظور تغییر شکل، محتوا، فراوانی یا مدت یک تجربۀ شخصی ناخواسته) است. این اجتناب تجربی به دو صورت نمایان می­شود؛ فرونشانی رفتاری^[[45]](#footnote-45)^ یا انجام اعمالی جهت فرار از تجارب آزاردهنده یا فاصله­گیری از شرایطی که با آغاز آن تجارب ذهنی به نحوی ارتباط دارند (پورفرج عمران، 1390). در درمان پذیرش و تعهد، این اجتناب، فرایند متضاد با پذیرش است و مخالف با درمان است (هیز و همکاران، 2011). طبق دیدگاه آگاهی، وقتی که فردی به محتوای تفکرات منفی عمیقاً باور دارد، ممکن است انعطاف پذیری روانشناختی و رفتاری­اش در پاسخ به این افکار کاهش یابد. مثلاً اگر یک فرد دیابتی عمیقاً این باور را داشته باشد که دیابتش منجر به مرگ زودرس او می­گردد، به احتمال بیشتری ممکن است از این افکار اجتناب کند و ممکن است فعالیت­های خود مراقبتی دیابت را نادیده بگیرد برای اینکه احساس ترسی نداشته باشد. این اجتناب تجربی زمانی اتفاق میافتد که فرد تمایلی ندارد تا در تماس با رویدادهای خصوصی خاص (مثل درد روانشناختی و بدنی، افکار، احساسات، حافظه و غیره) بماند (گریک و همکاران، 2010).

درمان مبتنی بر تعهد و پذیرش، شش فرایند مرکزی؛ پذیرش^[[46]](#footnote-46)^، عدم همجوشی^[[47]](#footnote-47)^، لحظۀ حال^[[48]](#footnote-48)^، خود به عنوان بافت یا زمینه^[[49]](#footnote-49)^، ارزشها^[[50]](#footnote-50)^ و عمل متعهدانه^[[51]](#footnote-51)^ دارد (پریودینی و همکاران، 2011). هدف اصلی ACT ایجاد انعطاف پذیری روانی^[[52]](#footnote-52)^ است، یعنی ایجاد توانایی انتخاب عملی در بین گزینه­های مختلف که مناسب­تر باشد، نه اینکه عملی صرفاً جهت اجتناب از افکار، احساسات، خاطره­ها یا تمایلات آشفته­ساز انجام شود یا بر فرد تحمیل گردد (فورمن و هربرت، 2009). یا افزایش توانایی فرد برای دادن پاسخ مؤثر به افکار و احساسات گوناگونی که هنگام بروز مشکلات ظاهر می­شوند (گریک و همکاران، 2010).

در این درمان، ابتدا سعی می­شود، پذیرش روانی^[[53]](#footnote-53)^ فرد در مورد تجارب ذهنی (مانند افکار و احساسات) افزایش یابد و متقابلاً اعمال کنترلی نامؤثر کاهش یابد. به بیمار آموخته می­شود که هر نوع عملی جهت اجتناب یا کنترل این تجارب ذهنی ناخواسته بی اثر است یا اثر معکوس دارد و موجب تشدید آن­ها می­شود و باید این تجارب را بدون هیچ گونه واکنش درونی یا بیرونی جهت حذف آن­ها، به طور کامل
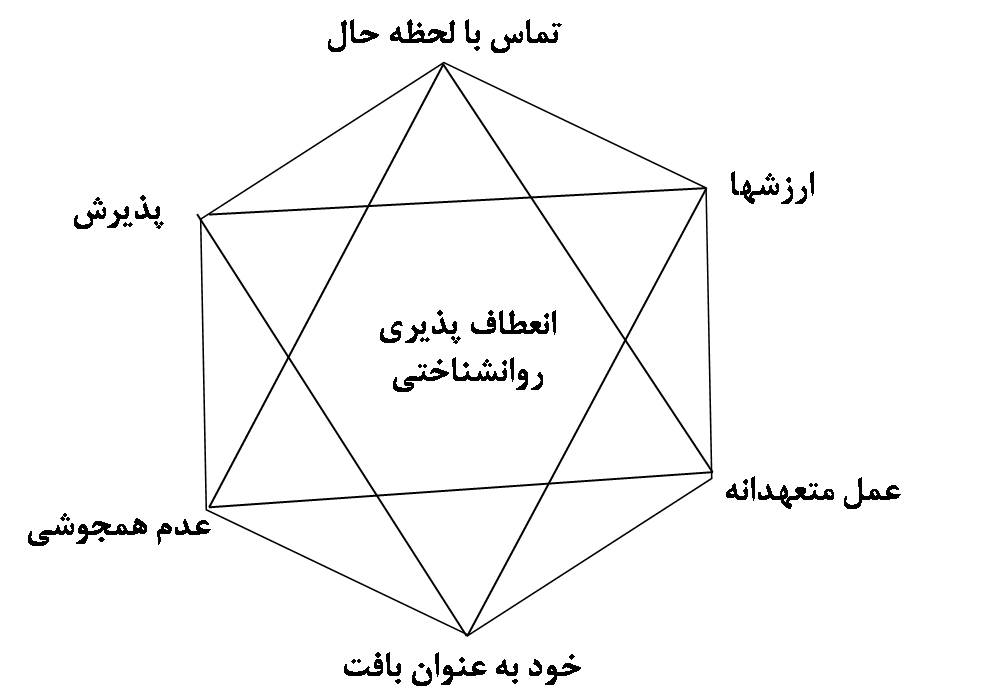
پذیرفت. در قدم دوم، بر آگاهی روانی^[[54]](#footnote-54)^ فرد در لحظۀ حال افزوده می­شود؛ یعنی فرد از تمام حالات روانی، افکار و رفتار خود در لحظۀ حال آگاهی می­یابد. در مرحلۀ سوم، به فرد آموخته می­شود که خود را از این تجارب ذهنی جدا سازد، به نحوی که بتواند مستقل از این تجارب عمل کند. چهارم، تلاش برای کاهش تمرکز مفرط بر خودتجسمی^[[55]](#footnote-55)^ یا داستان شخصی (مثل قربانی بودن) که فرد برای خود در ذهنش ساخته است. پنجم، کمک به فرد تا اینکه ارزش­های شخصی اصلی خود را بشناسد و به طور واضح مشخص سازد و آن­ها را به اهداف رفتاری خاص تبدیل کند (روشن­سازی ارزش­ها). در نهایت، ایجاد انگیزه جهت عمل متعهدانه^[[56]](#footnote-56)^ یعنی فعالیت معطوف به اهداف و ارزش­های مشخص­شده به همراه پذیرش تجارب ذهنی. این تجارب ذهنی می­توانند افکار افسرده­کننده، وسواسی، افکار مرتبط به حوادث، هراس­ها و یا اضطراب­های اجتماعی باشند (پورفرج عمران، 1390). درمان پذیرش و تعهد به افراد می­آموزد که از طریق تمرکز آگاهانه­تر بر فرایند تفکرشان و ایجاد ارتباط اینها با هدف مبتنی بر عمل احساسات خود را بپذیرند، خنثی کنند و یا از محتوای افکارشان خلاص شوند. در کل، درمان پذیرش و تعهد، در تلاش است تا به افراد بیاموزد که تفکرات و احساسات خود را تجربه کنند به جای اینکه آنها را تغییر دهند یا متوقف سازند (گریک و همکاران، 2007). در این درمان­ها به جای تغییر شناخت­ها سعی می­شود تا ارتباط روان­شناختی فرد با افکار و احساساتش افزایش یابد. مزیت عمدۀ درمان پذیرش و تعهد، نسبت به روان­درمانی­های دیگر، در نظر گرفتن جنبه­های انگیزشی به همراه جنبه­های شناختی، به جهت تأثیر و تداوم بیشتر اثر درمان است (پورفرج عمران، 1390).

به دلایلی که ذکر شد، این درمان برای بهبود شاخصهای جسمانی و روانشناختی بیماران مبتلا به دیابت نوع II پیشنهاد گردیده است، درمان پذیرش و تعهد ابتدا توسط استیوان هیز در دهۀ 1980 برای درمان اختلال­های مختلف معرفی گردید و شواهد تجربی در مورد تأثیر این روش درمانی بر اختلالات مختلف، رو به افزایش است برای مثال کارایی این روش درمانی در مورد اختلالاتی مثل؛ افسردگی (لاپالانین و همکاران، 2007؛ کانتر، بارچ و گینور، 2006)، درد مزمن (واولز و تامپسون، 2011)، PTSD (اورسیلو و باتن، 2005)، وسواس (توهیگ، هیز و ماسودا، 2006)، سوء مصرف مواد (گیفورد، کلنبرگ و هیز، 2004)، فرسودگی شغلی (بوند و بنس، 2003) و پسیکوزها (باچ و هیز، 2002) نشان داده شده است. درمان پذیرش و تعهد در بسیاری از بیماریهای جسمانی مزمن، نتایج مثبتی را در برداشته است حتی زمانی که به شکل بسیار مختصر ارائه گردیده است (هیز، 2004). اما این درمان برای دیابت نوع II، اولین بار توسط گریک (2004) برای طرح رسالۀ دکتری پیشنهاد گردید و وی اثر این درمان را در کاهش میزان هموگلوبین گلیکوزیله نشان داد و بعد از آن مطالعات در این زمینه تنها توسط گریک و همکارانش صورت گرفته است مانند؛ گریک و همکاران (2007) در مطالعۀ خود با هدف بررسی اثر درمان ACT بر بهبود هموگلوبین گلیکوزیله و خودمراقبتی، 81 بیمار دیابتی نوع II را مورد مطالعه قرار دادند و در پیگیری سه ماهه دریافتند که درمان سبب بهبود هموگلوبین گلیکوزیله و خودمراقبتی بیماران دیابتی شد. از سال 2004 تاکنون تحقیقات اندکی در این زمینه صورت گرفته است اما همانطور که در بیان مسئله نیز ذکر کردید جایگاه دو متغیر تعدیل­کنندۀ مهم سبک­های مقابله و حمایت اجتماعی نادیده گرفته شده است که در تحقیق حاضر سعی می­گردد نقش این دو متغیر در درمان فوق مد نظر قرار گیرد تا بتوان مدل جامع­تری برای درمان دیابت نوع II ارائه داد.

**سوال­های پژوهش:**

- آیا درمان مبتنی بر پذیرش و تعهد بر بهبود هموگلوبین گلیکوزیله بیماران دیابتی نوع II مؤثر است؟
- آیا درمان مبتنی بر پذیرش و تعهد بر بهبود خودمراقبتی بیماران دیابتی نوع II مؤثر است؟
- آیا درمان مبتنی بر پذیرش و تعهد بر بهبود کیفیت زندگی بیماران دیابتی نوع II مؤثر است؟
- آیا حمایت اجتماعی و سبک­های مقابله­ای در اثر درمان پذیرش و تعهد بر هموگلوبین گلیکوزیله، خودمراقبتی و کیفیت زندگی بیماران مبتلا به دیابت نوع II نقش تعدیل­کننده دارند؟

**اهداف پژوهش :**

**هدف کلی:** تعیین میزان اثر مدل درمان مبتنی بر پذیرش و تعهد بر بهبود هموگلوبین گلیکوزیله و شاخص­های روانی (خود مراقبتی، کیفیت زندگی) بیماران دیابتی نوع II با در نظر گرفتن نقش تعدیل­کنندگی سبک­های مقابله­ای و حمایت اجتماعی.

**اهداف جزئی:**

- تعیین میزان اثر درمان مبتنی بر پذیرش و تعهد بر بهبود هموگلوبین گلیکوزیله بیماران دیابتی نوع II
- تعیین میزان اثر درمان پذیرش و تعهد بر بهبود خود مراقبتی بیماران دیابتی نوع II
- تعیین میزان اثر درمان پذیرش و تعهد بر بهبود کیفیت زندگی بیماران دیابتی نوع II
- تعیین نقش تعدیل­کنندۀ سبک­های مقابله­ای در تأثیر درمان پذیرش و تعهد بر شاخص­های هموگلوبین گلیکوزیله، کیفیت زندگی و خودمراقبتی بیماران دیابتی نوع II
- تعیین نقش تعدیل­کنندۀ حمایت اجتماعی در تأثیر درمان پذیرش و تعهد شاخص­های هموگلوبین گلیکوزیله، کیفیت زندگی و خودمراقبتی بیماران دیابتی نوع II

**فرضيه­ها/ پيش فرض­ها:**

- میزان بهبود هموگلوبین گلیکوزیله بیماران دیابتی نوع II در گروه درمانی پذیرش و تعهد از گروه کنترل بیشتر است.
- میزان بهبود خود مراقبتی بیماران دیابتی نوع II در گروه درمانی پذیرش و تعهد از گروه کنترل بیشتر است.
- میزان بهبود کیفیت زندگی بیماران دیابتی نوع II در گروه درمانی پذیرش و تعهد از گروه کنترل بیشتر است.
- سبک­های مقابله­ای، تأثیر درمان پذیرش و تعهد بر شاخص­های هموگلوبین گليكوزيله، كيفيت زندگي و خودمراقبتي بيماران مبتلا به ديابت نوع II را تعديل مي­کند.
- حمایت اجتماعی، تأثیر درمان پذیرش و تعهد بر شاخص­های هموگلوبین گليكوزيله، كيفيت زندگي و خودمراقبتي بيماران مبتلا به ديابت نوع II را تعديل مي­کند.

**مواد و روش انجام تحقيق:**

**ابزار پژوهش:**

1. آزمایشهای تشخیصی HbA1C: هموگلوبين گليكوزيله (HbA1C)كسري از هموگلوبين است كه به آهستگي و طي فرآيندي غير آنزيمي از هموگلوبين و گلوكز شكل مي­گيرد. از آنجا كه گلبولهاي قرمز به گلوكز نفوذپذير هستند، هموگلوبين گليكوزيله در طي دوره زندگي گلبولهاي قرمز تشكيل مي­شود و ميزان آن بستگي مستقيم به غلظت گلوكز موجود در خون دارد. بنابراين، هموگلوبين گليكوزيله تاريخچه گلايسميكي از 120روز گذشتۀ فرد فراهم مي­آورد. هموگلوبين گليكوزيله تقريباً بايد هر 3 ماه اندازه­گيري شود تا وضعيت مديريت ديابت و كنترل گلايسميك بيمار مورد بررسي قرار گيرد. آزمايش HbA1C براي پايش اثربخشي درمان و به عنوان شاخصي براي تعيين نياز به تعديل در برنامه درماني به كار مي­رود (بروک، کلایتن و برون، 2006).
2. مقیاس کوتاه فعالیت­های خود مراقبتی دیابت (Summary of Diabetes Self-Care Activities): مقیاس SDSCA، شاخص خودتوصیفی معتبری برای خودمراقبتی دیابت است، 25 آیتم دارد که جنبه­های رژیم کلی، رژیم خاص، تست ورزش، گلوکز خون، مراقبت از پا و سیگار کشیدن را می­سنجد. نتایج 7 مطالعه نشان داد که این مقیاس از اعتبار و روایی مناسبی برخوردار است و نسبت به تغییر حساس است (توبرت، گلاسگو و هامپسون، 2002). رحیمیان­بوگر و همکاران (1390) در بررسی ابتدایی، آلفای کرونباخ کل مقیاس و خرده مقیاس­های هفتگی، ماهانه و سالانۀ را به ترتیب 95/0، 95/0، 84/0 و 74/0 به دست آورد که نشاندهندۀ همسانی درونی قابل قبول این مقیاس است.
3. پرسشنامه پذیرش و عمل دیابت Acceptance and Action Diabetes Questionnaire: تغییرات در فرایند درمان پذیرش و تعهد به وسیلۀ AADQ سنجیده می­شود. این پرسشنامه متشکل از 11 سوال است که احساسات و افکار مرتبط با دیابت را بر اساس پذیرش و ارزش عمل می­سنجد. آلفای کرونباخ ابزار 94/0 به دست آمده است که نشان­دهندۀ پایایی درونی خوبی است (گریک و همکاران، 2007).
4. کیفیت زندگی مشخصۀ دیابت: diabetes-dependent quality of life (ADDQoL) The audit of این ابزار جهت سنجش کیفیت زندگی افراد مبتلا به دیابت ساخته شده است (وانگ و یه، 2012). این مقیاس اختصاصی 19 آیتم دارد (اسپیت، رینی و بارنارد، 2009). نمره­گذاری از 1+ (اثر مثبت) تا 3- (اثر بسیار منفی) است (بردلی و اسپیت، 2002). این مقیاس از روایی و اعتبار مناسبی برخوردار است (گارات، اسمیت و فیتزپاتریک، 2002). آلفای کرونباخ آن در نسخۀ ایرانی 90/0 و در بازآزمایی ، 69/0 به دست آمده است که نشان می­دهد ابزار از پایایی خوبی برخوردار است (پورشریفی، 1386).
5. مقیاس چند­بعدی حمایت­اجتماعی ادراک­شده Multidimensional Scale of Perceived Social Support: یک مقیاس خود توصیفی 12 سوالی است که توسط زیمت و همکاران (1988) طراحی شده است. این مقیاس علاوه بر حمایت اجتماعی به طور کل، حمایت خانواده، دوستان و افراد مهم دیگر زندگی فرد را می­سنجد. ضریب آلفای آن در دامنه­ی 85/0 تا 91/0و با روش بازآزمایی 72/0 تا 85/0 است (ادواردز، 2004). در ایران نیز روایی و اعتبار آن به گزارش سلیمی و همکاران (1388)، روی نمونۀ 436 دانشجویان، مناسب گزارش شده است و آلفاي کرونباخ براي هر یک از ابعاد به ترتیب 86/0، 86/0 و 82/0 بود.
6. فرم کوتاه پرسشنامۀ سبک­های مقابله­ایThe Brief COPE Questionnaire : فرم ابتدایی پرسشنامه توسط کارور و همکاران (1989) در 60 آیتم طراحی شد. در تحقیق حاضر برای ارزیابی سبک­های مقابله­ای از فرم 28 سوالی آن استفاده ­می­گردد که در سال 1996 توسط کارور طراحی شد، آلفای کرونباخ محاسبه شده برای کل آزمون و زیر مقیاس­ها از 56/0 تا 89/0 به دست آمد. نتایج تحلیل عاملی و بازآزمایی، روایی و اعتبار این آزمون را نشان داد (فیلون،کواکس، گاگنون و اندلر، 2002).

**روش انجام تحقیق :**

پژوهش حاضر، کارآزمایی بالینی است.100 نفر از بیماران مبتلا به دیابت نوع II که بر اساس علائم جسمانی و آزمایش خون توسط متخصص مربوطه تشخیص دیابت نوعII داده شده اند، به طور تصادفی در دو گروه زیر قرار میگیرند:

گروه اول: درمان­های دارویی و کارگاه یک روزه دیابت

گروه دوم: درمانهای دارویی و کارگاه یک روزه دیابت به علاوه درمان مبتنی بر پذیرش تعهد.

کارگاه یک روزه دیابت: کارگاه آموزشی یک روزه شامل مدیریت و مراقبت تغذیه، بهبود فعالیت بدنی، تنظیم هموگلوبین گلیکوزیله، استفاده منظم از داروهای تجویز شده است.

**جامعه آماري و تعداد نمونه:**

در مطالعۀ حاضر، افراد مراجعه­کننده به آزمایشگاه بیمارستان لبافی­نژاد، پس از بررسی­های اولیه و انجام آزمایشها و تشخیص دیابت نوع II (در صورت تمایل به شرکت در تحقیق) توسط متخصص مربوطه، به محقق ارجاع داده می­شوند. محقق از بیماران دیابت نوع II، مصاحبۀ بالینی و ویزیت اولیه انجام می­دهد و 100 نفر از افرادی را که معیارهای ورودی زیر را داشته باشند، با اخذ رضایت آگاهانه، وارد مطالعه می­کند:

1. دامنۀ سنی 60- 40 سال
2. لازم است آزمودنیها در طول اجرای پژوهش از برنامه­های روان درمانی و تغییر درمان­های دارویی در خارج از مداخلۀ آموزشی و درمانی پژوهش استفاده نکنند.

معیارهای خروجی:

1. ابتلا به انواع دیگر دیابت
2. ابتلا به عوارض دیابتی شدید که مانع از انجام تحقیق می­گردد.

جمعيت مطالعه (Study population) :

کلیه بیماران مبتلا به دیابت نوع II مراجعه­کننده به بخش غدد و متابولیسم بیمارستان لبافی­نژاد.

شيوه نمونه گيري (Sampling Method) :

روش نمونه­گیری در تحقیق حاضر، نمونه­گیری هدفمند در دسترس است،

حجم نمونه و نحوه محاسبه آن (Sample Size) :

روش نمونه­گیری در تحقیق حاضر، نمونه­گیری هدفمند در دسترس است، ابتدا از بین بیمارانی که به بیمارستان لبافی­نژاد مراجعه می­کنند و از بین بیماران دیابتی تشخیص داده شده با در نظر گرفتن معیارهای ورودی،100 نفر از کسانی که تمایل به شرکت در تحقیق را دارند (با اخذ رضایت کتبی) از طریق جایگزینی تصادفی به دو گروه مداخله (گروهی که درمانهای دارویی و آموزش جاری دپارتمان دیابت را به علاوه درمان پذیرش و تعهد دریافت می­کنند) و کنترل (گروهی که تنها درمانهای دارویی و آموزش جاری دپارتمان دیابت را دریافت می­کنند) تقسیم می­شوند. با استناد به باسا^[[57]](#footnote-57)^ (1995)، که تغییرات میانگین HbA1c را قبل و 3 ماه بعد از یک برنامه مداخله به میزان (7/1=1/7-8/8) و جهت انحراف معیار، میزان (2/2) را محاسبه نموده است (05/0=α و 2/0=β) و با استفاده از فرمول­های زیر نمونۀ لازم برای این بررسی 35 نفر محاسبه شد که با در نظر داشتن دو متغیر تعدیل کننده و احتساب افت آزمودنی برای هر گروه نمونه حدود 50 نفر مد نظر قرار گرفت.

$d=\frac{\mu_{1}-\mu_{2}}{\delta\sqrt{2}}$ $n=\frac{(z_{1-\frac{\propto}{2}}+{z_{1-\beta})}^{2}}{d}$

$d=\frac{8.8-7.1}{2.2\sqrt{2}}=\frac{1.7}{3.112}=0.5464$ $n=\left( \frac{1.96+1.28}{0.5464} \right)^{2}=35$

تمامی آزمودنی­ها در هر دو گروه در بدو ورود و قبل از مداخلات آزمایشی، در مرحله پیش از مداخله پرسشنامه­های اطلاعات جمعیت­شناختی، مقیاس خود مراقبتی دیابت، پرسشنامه کیفیت زندگی، مقیاس سبک­های مقابله­ای، مقیاس حمایت اجتماعی ادراک­شده و پرسشنامۀ پذیرش و عمل دیابت را تکمیل می­کنند و برای تعیین میزان هموگلوبین گلیکوزۀ بیماران، از آنها آزمایش خون HbA1c گرفته می­شود, علاوه بر این و با توجه به شواهد علمی ذکر شده در مورد شیوع افسردگی در این بیماران، برای کنترل افسردگی از مقیاس کوتاه افسردگی بک استفاده می­گردد. مداخله درمانی طی 10 جلسه ACT صورت می­گیرد و در هر گروه درمانی باید 8 تا 10 نفر حضور داشته باشد و هر جلسه یکبار در هفته و به مدت 2 ساعت، زمان می­برد. در پایان مداخلات درمانی، در مرحله پس­آزمون، به منظور سنجش تأثیر روش کاربندی، هر دو گروه مجدداً مقیاس خود مراقبتی دیابت، پرسشنامه کیفیت زندگی، مقیاس سبک­های مقابله­ای، مقیاس حمایت اجتماعی ادراک­شده و پرسشنامۀ پذیرش و عمل دیابت را تکمیل می­کنند و برای بررسی تغییرات هموگلوبین گلیکوزیله، آزمایش خون مجدد گرفته می­شود. همچنین به منظور پیگیری اثرات درمانی، از آزمودنی­ها سه ماه پس از پایان پس آزمون، آزمایش خون مجدد انجام می­شود. پس از انجام تحقیق، برای رعایت اصول اخلاقی بیماران، درمان پذیرش و تعهد برای گروه کنترل نیز اجرا خواهد شد.

**شيوه تصادفي کردن (Random Allocation or Randomization) :**

ابتدا از بین بیماران مبتلا به دیابت نوع II که به بخش غدد و متابولیسم بیمارستان لبافی­نژاد مراجعه می­کنند، با در نظر گرفتن معیارهای ورودی،100 نفر از کسانی که تمایل به شرکت در تحقیق را دارند (با اخذ رضایت کتبی) از طریق جایگزینی تصادفی به دو گروه مداخله و کنترل تقسیم می­شوند (با استفاده از جدول اعداد تصادفی) و پس از جایگزینی تصادفی، برای رعایت اصل کاربندی تصادفی، دوباره از روی تصادف یکی از گروه به عنوان گروه مداخله (گروهی که درمانهای دارویی و کارگاه یک روزه دیابت را به علاوه درمان پذیرش و تعهد دریافت می­کنند) و گروه دیگر به عنوان گروه کنترل (گروهی که تنها درمانهای دارویی و کارگاه یک روزه دیابت را دریافت می­کنند) انتخاب می­گردد.

**تجزیه و تحلیل آماری داده­ها:**

براي تحليل اطلاعات به دست آمده علاوه بر استفاده از شيوه­هاي آمار توصيفي مانند فراواني، درصد، ميانگين و انحراف استاندارد كه براي نمايش و خلاصه كردن اطلاعات به دست آمده استفاده مي­شود، جهت تحلیل اطلاعات و ارزیابی نقش برنامه مداخله و کنترل اثر پیش آزمون از مدل کو واریانس و تحلیل واریانس با اندازه های مکرر و برای بررسی نقش تعدیل کنندگی متغیرهای مورد نظر پژوهش از تحلیل کواریانس استفاده خواهد شد.

1. محدوديتهاي اجرايي طرح وروش كاهش آنها :

همکاری پایین بیماران دیابتی در برنامه مداخله که باید از روشهای تقویتی برای بالا بردن همکاری استفاده کرد. البته خود بیماران پیشنهاد سرویس رایگان برای حضور در بیمارستان را داده اند.

**استفاده­کنندگان از نتیجه رساله:**

متخصصین سلامت و درمان در زمینۀ دیابت، روانپزشکان، روانشناسان و بیماران مبتلا به دیابت نوع II

**جنبۀ جدید بودن و نوآوری:**

جنبۀ نوآوری تحقیق حاضر عبارت است از در نظر گرفتن نقش دو متغیر سبک­های مقابله­ای و حمایت اجتماعی در مدل درمانی پذیرش و تعهد بر تنظیم هموگلوبین گلیکوزیله و شاخص­های روانشناختی بیماران مبتلا به دیابت نوع II است. همانطور که مطالعات فوق نشان داد با اینکه سبک­های مقابله­ای و حمایت اجتماعی اثر معناداری بر خودمراقبتی بیماران دیابت نوع IIداشت اما در مدل درمانی فوق که برای دیابت نوع II به کار رفته است مطرح نشده­اند. لذا در تحقیق حاضر، سعی می­شود تا با استفاده از مدل درمانی پذیرش و تعهد و در نظر گرفتن متغیرهای فوق، به مدل جامع­تری در درمان دیابت نوع II که متناسب با جامعۀ ایرانی بیماران دیابتی باشد، دست یابیم.

**Reference**

برمن، ر.، کلیگمن، ر. . جنسن، ه. (2004). *رشد تکامل*. مترجمان: طاهرپور، آرزو و طاهرپور، ژیوا. (1383). تهران: موسسۀ انتشاراتی تیمورزاده. نشر طبیب. ص63

بنکداران، شکوفه و افخمی زاده، مژگان. (1389). اثر کلسیتریول بر کنترل قند و لیپید در بیماران دیابتی نوع دو. *مجله ی غدد درون ریز و متابولیسم ایران*. دانشگاه علوم پزشکی و خدمات بهداشتی- درمانی شهید بهشتی. 12،5: 519-513

پورشریفی، حمید. (1386). تأثیر مصاحبۀ انگیزشی بر بهبود شاخصهای سلامت در افراد مبتلا به دیابت نوع دو. رساله دکتری. تهران: دانشگاه تهران.

پورفرج عمران، مجید. (1390). اثر درمان گروهی پذیرش و تعهد بر هراس اجتماعی دانشجویان. *فصلنامه دانش و تندرستی*.6، 2، 5-1

دیماتئو، ام.رابین. (1378). *روانشناسی سلامت.* ترجمه جمعی از نویسندگان زیر نظر کیانوش هاشمیان، تهران: انتشارات سمت.

دلاوری، علی­ رضا، مهدوی هزاره، علی­رضا، نوروزی نژاد، عباس و یاراحمدی، شهین، 1383. کارشناس تغذیه و دیابت (*برنامه ی کشوری پیشگیری و کنترل دیابت*). تهران: مرکز نشر صدا.

رحیمیان بوگر ا، بشارت م­ع، مهاجری تهرانی م­ح و طالع­پسند س. (1390). نقش پیشبین خودکارآمدي، باور به اثر درمان و حمایت اجتماعی در خودمدیریتی دیابت قندي. *مجله روانپزشکی و روانشناسی بالینی ایران*، سال 17، شماره3. 240 – 232

سلیمی ع، جوکار ب و نیک­پور ر.(1388). ارتباطات اينترنتي در زندگي: بررسي نقش ادراك حمايت اجتماعي و احساس تنهايي در استفاده از اينترنت. *مجلۀ مطالعات روانشناختی*، 5(3)، 102-81

كرتيس، آنتوني جيمز. (2000). روان‌شناسي سلامت، ترجمه علي فتحي‌آشتياني و همكاران (1385)، تهران، مؤسسه انتشارات بعثت.

نخعی، ن. (1387). روند ابتلا به دیابت در ایران رو به افزایش است*. همایش روز جهانی دیابت*. دانشگاه علوم پزشکی کرمان. آبان.

Alvin CP. (2008). Diabetes Mellitus In: Fauci AS, Braunwald E, Kasper DL, Hauser SL, Longo DL, Jameson JL. *Harrison’s principles of internal medicine*, 17th ed. NY: McGraw Hill: 2275-300.

American Diabetes Association. (2008). [Economic Costs of Diabetes in the U.S.in 2007. *Diabetes Care*](http://www.google.com/url?sa=t&rct=j&q=&esrc=s&frm=1&source=web&cd=1&ved=0CHAQFjAA&url=http%3A%2F%2Fcare.diabetesjournals.org%2Fcontent%2F31%2F3%2F596.abstract&ei=rM7-T8f8IcbPtAabl9jXBQ&usg=AFQjCNElNO_hYKuuPdxYuy5qHXUS0JkBSQ&sig2=l2w_Lk7VCieUxxI-eH2Oqw). 31,3:596-615.

Amsberg S, Anderbro T, Wredling R, Lisspers J, Lins PE, Adamson U, Johansson UB.(2009). A cognitive behavior therapy-based intervention among poorly controlled adult type 1 diabetes patients—A randomized controlled trial. *Patient Education and Counseling*. 77: 72–80

Anderson RJ, Freedland KE, Clouse RE, Lustman PJ. (2001). the prevalence of comorbid depression in adults with diabetes: a meta-analysis. *Diabetes Care*; 24: 1069–1078.

Attari A, Sartippour M, Amini M, Haghighat S. (2006). Effect of stress management training on glycemic control in Patients with type 1 diabetes. *Diabetes Res Clin Pract*; 73: 23-28.

Azizi F, Gouya MM, vazirian P, Dplatshahi P and Habibian S. (2003). The diabetes prevention and control program of the Islamic Republic of Iran. *Eastern Mediterranean Health Journal*. 91: 114–21.

Bach P, Hayes SC. (2002). The use of acceptance and commitment therapy to prevent the rehospitalization of psychotic patients: A randomized controlled trial. *Journal of Consulting and Clinical Psychology*. 70(5):1129-1139.

Basa RP. (1995). Evaluation of a diabetes specialty center: Structure, process and out come. *Patient Educ Couns*, 25:23–29.

Bastiaens H, Sunaert P, Wens J, Sabbe B, Jenkins L, Nobels F, Snauwaert B and Royen PV. (2009). Supporting diabetes self-management in primary care: Pilot-study of a group-based programme focusing on diet and exercise. *primary care diabetes*, 3: 103–109

Beck, AT. (1988). Beck Depression Inventory (BDI). Sidcup: *The Psychological Corporation*.

Belchetz P and Hammond P. (2003). *Diabetes and Endocrinology*. London: Mosby Co. p: 88

Bond FW & Bunce D. (2003). The role of acceptance and job control in mental health, job satisfaction, and work performance. *J Applied Psychology*. 88:1057-1067.

Boyle S, Allan C and Millar K. (2004). Cognitive-behavioural interventions in a patient with an anxiety disorder related to diabetes. *Behaviour Research and Therapy*. 42: 357–366

Bradley C & Speight J. (2002). Patient perceptions of diabetes and diabetes therapy: Assessing quality of life. *Diabetes/Metabolism Reviews*, 18(S3), S64–S69.

Brook C. Clayton P. Brown R. (2006). *Brook's clinical pediatric endocrinology*. Oxford: Blackwell. p:458.

Brunner LSH and Suddarth DS. (2004). *Text book of Medical Surgical Nursing*, 10th Edition. Lippincott Williams. Chapter 2.

Butler JT. (2001). *Principles of health education and health promotion* [2nd Edition](http://www.chegg.com/textbooks/principles-of-health-education-health-promotion-2nd-edition-9780895823403-0895823403): Morton Publishing Company.

Callaghan P, Morrissey J. (1993). Social support and health: a review. *J Adv Nurs*. 18: 203-210.

Campbell LK, White JR, Campbell RK. (1996). Acarbose: its role in the treatment of diabetes mellitus. *Ann Pharmacother*. 30:1255-62.

Caballero AE. (2006). Building cultural bridges: understanding ethnicity to improve acceptance of insulin therapy in patients with type 2 diabetes. *Ethn Dis*. 16(2):559-68.

Carver CS, Scheier MF, & Weintraub JK. (1989). Assessing coping strategies: A theoretically based approach. *Journal of Personality and Social Psychology, 56,267-283.*

Coelho R, Amorim I, Prata J. (2003). Coping styles and quality of life in patients with non-insulin-dependent diabetes mellitus. *Psychosomatics*. 44:312–318

Chinn P, Karmer M. (1999). Theory and nursing. St. Louis: Mosby: 61-70.

Chiu KC, Chu A, Go VL, Saad MF. (2004). Hypovitaminosis D is associated with insulin resistance and beta cell dysfunction. *Am J Clin Nutr*. 79: 820-5.

Cohen M, Crosbie C, Cusworth L, Aimmet P. (1984). Insulin – not always a life sentence; withdrawal of insulin therapy in non-insulin-dependent diabetes. *Diabetes Res*; 1:31–34.

Cooppan R, Wyckoff J & Abrahamson MJ, Joslis'septoplasty. (2005). General Approach to the Treatment of Diabetes Mellitus & Diabetic Complications: Clinical Aspects, Ramachandiran, Diabetes Mellitus, USA, *Lippincott Williams & Wilkins*, 14th Edition, P: 585-794 & 885-1144.

Corey G. (2008). *Theory and practice of group counseling* (8th ed.). Belmont, CA: Thompson Brooks/Cole. Pp:347

Cox D, Gonder-Frederick L, McCall A, Kovatchev B and Clark W. (2002). The effects of glucose fluctuation on cognitive function and quality of life: the functional costs of hypoglycaemia among adults with type 1 or type 2 diabetes. *Int J Clin Pract*; 129: 20–26.

Cusick MC, Meleth AD, Agron E, Fisher MR, Reed GF, Knatterud GL, Barton FB, Davis MD, Ferris FL, and Chew FY. (2005). Associations of Mortality and Diabetes Complications in Patients with Type 1 and Type 2 Diabetes. *Diabetes Care*. 28,3: 617-625.

DCCT Research Group (The Diabetes Control and Complications Trial Research Group). (1993). The effect of intensive treatment of diabetes on the development and progression of long-term complications in insulin-dependent diabetes mellitus. *N Engl J Med*; 329:977-86.

DeCoster V and Cummings S. (2004). Coping with Type 2 Diabetes: Do Race and Gender Matter?*. Social work in health care*. 40, 2: 37-53

De Groot M, Anderson R, Freedland KE, Clouse RE, Lustman PJ. (2001). Association of depression and diabetes complications: a meta-analysis. *Psychosom Med*; 63: 619–630.

Delavari A, Mahdavi Hazaveh A, Norozi Nejad A, Yarahmadi Sh, Taghipour M, editors. (2003). The National Diabetes Prevention and Control Programme. *Iranian Health Ministry*. Diseases Management Centre. Tehran: Seda

DiMatteo, M. R. (1991). *The psychology of health, illness and medical care*: an individual perceptive: California Book/ Cole publishing company.

Edwards LM. (2004). Measuring Perceived Social Support in Mexican American Youth: Psychometric Properties of the Multidimensional Scale of Perceived Social Support Hispanic, *Journal of Behavioral Sciences*. 26, 2: 187-194.

Endocrine and Metabolism Research Center. (2002). Tehran Lipid & Glucose Study Methodology and Summarizde Findings.

Feinglos MN, Hastedt P, Surwit RS. (1987). Effects of relaxation therapy on Patients with type I diabetes mellitus. *Diabetes Care*.10 (1): 72–75.

Fillion L, Kovacs AH, Gagnon P, Ender NS. (2002). Validation of the Shortened COPE for use with Breast Cancer Patients Undergoing Radiation Therapy. Current Psychology*: Developmental Learning Personality Social Spring*. 21, 1: 17-34.

Fisher KL. (2006). Assessing psychosocial variables: a tool for diabetes educators. *Diabetes Educ*. 32, 1:51-58.

Fisher L, Bartz R. (1998). The family and type II diabetes: a framework for intervention*. Diabetes Educ*. 24:599–607

Forman, E. M., & Herbert, J. D. (2009). *New directions in cognitive behavior therapy: Acceptance-based therapies*. In W. O'Donohue & J. E. Fisher (Eds.), General principles and empirically supported techniques of cognitive behavior therapy. 2nd ed. Hoboken, NJ: Wiley: 102–114

Foster D, Wilson J. (2008). Diabetes Mellitus In: Larsen PR, kronenberg H, Melmed S, Polonsky K. Willims text book of endocrinology, 11th ed. PA: *Saunders*: 1329-417.

Fower MJ. (2008). Microvascular and macrovascular complications of diabetes. *Clin Diab*. 26(2): 77-82.

Franken R. (2003). *Human motivation. 5^th^ ed. United States*; [Academic Internet Publishers Incorporated](http://www.fishpond.co.nz/c/Books/p/Academic+Internet+Publishers+Incorporated). Wadsworth/Thomson Learning pp:529.

Franklin MD. (2008). The relationship between psychosocial factors, self- care behaviors, and metabolic control in adolescents with type II diabetes. *Dissertation for psychology*. St. TN: Vanderbil Univ.

Funnell MM, Anderson RM. (2004). Empowerment and self-management education. *Clinical Diabetes*; 22: 123–127.

Funnell MM, CDE RN, and. Anderson RM. (2004). Empowerment and Self-Management of Diabetes. *Clinical Diabetes*. 22, 3: 123-127.

Gafvels C, Wandell PE. (2006). Coping strategies in men and women with type 2 diabetes in Swedish Primary Care. *Diabetes Research and Clinical Practice*; 71: 280–289.

Garratt A. M Schmidt L and Fitzpatrick R. (2002). Patient-assessed health outcome measures for diabetes: a structured review. *Diabetes UK.* Diabetic Medicine.19:1-11

Gifford EV, Kohlenberg BS, Hayes SC, Antonuccio DO, Piasecki MM, Rasmussen-Hall ML. (2004). Acceptance-Based Treatment for Smoking Cessation. *Behavior Therapy*. 35: 689-705.

Gillibrand R, stevenson J. (2006). The extended health belief model applied to the experience of diabetes in young people. *Br J Health Psychol*. 11,1:155-69.

Glasgow RE, Toobert DJ, Gillette CD. (2001). Psychosocial barriers to diabetes self-management and quality of life. *Diabetes Spectrum*. 14, 1: 33-41

Glasgow RE, Hampson SE, Strycker LA, Ruggiero L. (1997). Personal-model beliefs and social-environmental barriers related to diabetes self-management. *Diabetes Care*. 20: 556-61

Gaudiano BA (2009). Öst’s (2008) methodological comparison of clinical trials of Acceptance and Commitment Therapy versus Cognitive Behavior Therapy: matching apples with oranges? *Behaviour Researc and Therapy*. 47: 1066-1070.

Grey M. (2000). Coping and diabetes. *Journal of diabetes spectrum*. 13(3): 167.

Gregg J A, Schmidt E, Ward K, Almada P and Knezevich P. (2010). An alternative model for understanding the diabetes-depression relationship: the meditational role of thought believe ability. *Journal of Behavioral Health and Medicine*. 1**,** 15-23

Gregg J A, Callaghan G M and Hayes S. (2007). Improving diabetes self-management through acceptance, mindfulness, and values: a randomized controlled trial. Journal of Consulting and Clinical Psychology. 75,2 :336-343.

Haque M, Emerson SH, Dennison CR, Navsa M, Levitt NS. (2005). Barriers to initiating insulin therapy in patients with type 2 diabetes mellitus in publicsector primary health care centres in Cape Town. *S Afr Med J*. 95(10):798-802.

Hasker SM. (2010). Evaluation of the Mindfulness-Acceptance-Commitment (MAC) Approach for Enhancing Athletic Performance. *Dissertation for the Degree Doctor of Psychology*. Indiana University of Pennsylvania August. P:16

Hayes SC. (2004). Acceptance and commitment therapy, relational frame theory, and the third wave of behavioral and cognitive therapies. *Behavior Therapy*. 35,4: 639-665.

Hayes SC and Duckworth MP. (2006). Acceptance and Commitment Therapy and Traditional Cognitive Behavior Therapy Approaches to Pain. *Cognitive and Behavioral Practice*. 13:185–187

Hayes SC, Levin ME, Plumb-Vilardaga J, Villatte JL and Pistorello J. (2011). Acceptance and Commitment Therapy and Contextual Behavioral Science: Examining the Progress of a Distinctive Model of Behavioral and Cognitive Therapy. *Behavior Therapy*. 10.1016/j.beth.2009.08.002

Henricsson M, Nystrom L, Blohme G, Ostman J, Kullberg C, Svensson M, Scholin A, Arnqvist HJ, Bjork E, olinder J, Eriksson JW, and Sundkvist G. (2003). The Incidence of Retinopathy 10 Years After Diagnosis in Young Adult People With Diabetes. *Diabetes Care.* 26(2): 349-354.

Hirsch IB. (2003). The burden of diabetes (care). *Diabetes Care*. 26: 1613–4

Hill Golden S, Wang N-YJ, Klag MA, Meoni LL, Brancati F. (2003). *Blood pressure in young adulthood and the risk of type 2 diabetes in middle age*. *Diabetes Care*. 26(4):1110-15

Hobbis IC, Sutton S. (2005). Are techniques used in cognitive behaviour therapy applicable to behaviour change interventions based on the theory of planned behaviour? *J Health Psychol*.10:7–18.

Hofmann, S. G., & Asmundson, G. J. G. (2008). Acceptance and mindfulness-based therapy: New wave or old hat? *Clinical Psychology Review*, 28, 1–16.

Housiaux M., Luminet O. Van Broeck N. Dorchy H. )2010(. Alexithymia is associated with glycaemic control of children with type 1 diabetes. *Diabetes & Metabolism*. 36: 455-462.

Heitzmann CA and Kaplan RM. (1984).Interaction between sex and social support in the control of type II diabetes mellitus. *Journal of Consulting and Clinical Psychology*, 52, 6: 1087-1089

Ikebukuro K, Adachi Y, Yamada Y, Fujimoto S, Seino Y, Oyaizu H, Hioki K and Ikehara S. (2002). Treatment of streptozotocininduced diabetes mellitus by transplantation of islet cells plus bone marrow cells via portal vein in rats. *Transplantation.* Feb 27; 73(4): 512-8.

Jablon SL, Nabiloff BD, Gilmore SL, Rosenthal MJ. (1997). Effects of relaxation training on glucose tolerance and diabetic control in type II diabetes. *APPl Psychophysiol Biofeedback*; 22: 155-169.

Kahn RC, Weir GC, King Alan GL, Jacobson M, Moses AC, Smith RJ. (2005) .General Approach to the Treatment of Diabetes Mellitus & Diabetic Complications: *Clinical Aspects*, Chapter 34.

Kanter JW, Baruch DE, Gaynor ST. (2006). Acceptance and Commitment Therapy and Behavioral Activation for the Treatment of Depression: *Description and Comparison. The Behavior Analyst*. 29:161–185.

[Kaplan RM](http://www.ncbi.nlm.nih.gov/pubmed?term=Kaplan%20RM%5BAuthor%5D&cauthor=true&cauthor_uid=3678167), [Hartwell SL](http://www.ncbi.nlm.nih.gov/pubmed?term=Hartwell%20SL%5BAuthor%5D&cauthor=true&cauthor_uid=3678167). (1987). Differential effects of social support and social network on physiological and social outcomes in men and women with type II diabetes mellitus. *Health Psychology*; 6(5):387-98.

Lappalainen R, Lehtonen T, Skarp E, Taubert E, Ojanen M, & Hayes SC. (2007). The impact of CBT and ACT models using psychology trainee therapists. *Behavior Modification*, *31*(4), 488-511.

Lin EH, Katon W, Vo Korff M, Rutter C, Simon GE, Oliver M, Ciechanowaski p. Lundman EJ, Bush T and Young B. (2004). Relationship of depression and diabetes self-care, medication adherence, and preventive care. *Diabetes Care*; 27: 2154–2160.

[Liu MY](http://www.ncbi.nlm.nih.gov/pubmed?term=%22Liu%20MY%22%5BAuthor%5D), [Tai YK](http://www.ncbi.nlm.nih.gov/pubmed?term=%22Tai%20YK%22%5BAuthor%5D), [Hung WW](http://www.ncbi.nlm.nih.gov/pubmed?term=%22Hung%20WW%22%5BAuthor%5D), [Hsieh MC](http://www.ncbi.nlm.nih.gov/pubmed?term=%22Hsieh%20MC%22%5BAuthor%5D), [Wang RH](http://www.ncbi.nlm.nih.gov/pubmed?term=%22Wang%20RH%22%5BAuthor%5D). (2010). Relationships between emotional distress, empowerment perception and self-care behavior and quality of life in patients with type 2 diabetes*.* [*Hu Li Za Zhi,*](http://www.ncbi.nlm.nih.gov/pubmed/20401867) 57(2):49-60.

[Lustman PJ](http://www.ncbi.nlm.nih.gov/pubmed?term=Lustman%20PJ%5BAuthor%5D&cauthor=true&cauthor_uid=10895843), [Anderson RJ](http://www.ncbi.nlm.nih.gov/pubmed?term=Anderson%20RJ%5BAuthor%5D&cauthor=true&cauthor_uid=10895843), [Freedland KE](http://www.ncbi.nlm.nih.gov/pubmed?term=Freedland%20KE%5BAuthor%5D&cauthor=true&cauthor_uid=10895843), [de Groot M](http://www.ncbi.nlm.nih.gov/pubmed?term=de%20Groot%20M%5BAuthor%5D&cauthor=true&cauthor_uid=10895843), [Carney RM](http://www.ncbi.nlm.nih.gov/pubmed?term=Carney%20RM%5BAuthor%5D&cauthor=true&cauthor_uid=10895843). (2000). Depression and poor glycemic control: A meta-analytic review of the literature. *Diabetes Care*. 23(7): 934- 42.

Lustman PJ and Gavard JA. (2012). Chapter 24; Psychosocial Aspects of Diabetes in Adult Populations. Diabetes in America, 2nd Edition Table of Contents. Washington University School of Medicine, St. Louis, MO: 507-517.*Available: http://diabetes.niddk.nih.gov/dm/pubs/america/contents.aspx*

Melchior WR, Jaber LA.( 1996). Metformin: an antihyperglycemic agent for treatment of type II diabetes. *Ann Pharmacother*. 30:158-64.

Mollema ED, Snoek FJ, Heine RJ, van der Ploeg HM. (2001). Phobia of self-injecting and selftesting in insulin-treated diabetes patients: opportunities for screening. *Diabet Med.*18(8):671-4.

Mooradian AD. (1996) .Drug therapy of non-insulin-dependent diabetes mellitus in the elderly. *rugs*; 51:931-41

Murugesan N, Shobana R, Snehalatha C.(2009). Immediate impact of diabetes training programmer for primary care physicians-An endeavor for national capacity building for diabetes management in India. *Diabetes Res Clin Pract.* 83 (1): 140-4.

Norris SL, Engelgau MM, Naranyan KMV. (2001). Effectiveness of self-management training in type 2 diabetes: a systematic review of randomized controlled trials. *Diabetes Care*; 24: 561–587.

Norris SL, Lau J, Smith SJ, Schmid CH, Engelgau MM. (2002). Self-management education for adults with type 2 diabetes: a meta-analysis on the effect on glycemic control. *Diabetes Care;* 25: 1159–1171.

Orsillo SM, Batten S. (2005). Acceptance and Commitment Therapy in the Treatment of Posttraumatic Stress Disorder. *Behavior Modification*. 29, 1: 95-129.

Peyrot M, Rubin R, Siminerio L. (2002). Physician and nurse use of psychosocial strategies and referrals in diabetes. *Diabetes*; 51 (Suppl. 2): A446.

Pincus T, Burton AK, Vogel S, Field AP. (2002). A systematic review of psychological factors as predictors of chronicity/disability in prospective cohorts of low back pain. *Spine*; 27(5): 109-20.

Polonsky WH. (2002). Emotional and quality of life aspects of diabetes management. *Curr Diab Rep*; 2: 153-9.

Prevedini A B, Presti G, Rabitti E, Miselli G, Moderato P. (2011). Acceptance and Commitment Therapy (ACT): the foundation of the therapeutic model and an overview of its contribution to the treatment of patients with chronic physical diseases. Giornale Italiano di Medicina del Lavoro ed Ergonomia Supplemento A, *Psicologia* 33, 1: A53-A63

Raile k, Galler A, Hofer S, Herbst A, Dunstheimer D, Busch P, and Holl RW.(2007). Diabetic Nephropathy in 27, 805 Children, Adolescents, and Adults with Type 1 Diabetes. *Diabetes Care*. 30,10: 2523-2528.

Rathus SA. (1990). *Psychology*. Holt, Rinehart and Winston. pp:240.

Reaven GM. (1988). Role of insulin resistance in human disease. *Diabetes*; 37: 1595–1607.

Rendell M. (1983). C-peptide levels as a criterion in the treatment of maturity-onset diabetes. *J Clin Endocrinol Metab*; 57: 1198–1206.

Rodriguez LM, Castellanos VH. (2000). Use of low-fat foods by people with diabetes decreases fat, saturated fat, and holesterol intakes. *J Am Diet Assoc*. 100: 531-6.

Rollnick S, Miller WR, Butler CC. (2008). *Motivational interviewing in health care: Helping patients change behavior.* New York: The Gilford Press. p. 17-19

Rubin RR. (2000). Psychotherapy and counseling in diabetes mellitus. In: Snoek FJ, Skinner TC (editors). *Psychology in diabetes care*. John Wiley & Sons, Ltd: 235-264.

Rubin RR. (2002). Hypoglycemia and quality of life. *J* *Diabetes Care*; 26: 60–63.

Rubin RR, Peyrot M. (2001). psychological issues and treatments for people with diabetes. J *Clin Psychol.* 57:1640-1657.

Rubin, R. R., & Napora, J. P. (2001). Behavior change. In: J. F. Marion, K. Polonsky, W. H. Polonsky, P. Yarborough, & V. Zamudio (Eds.), A core curriculum for diabetes education ;4th ed. Chicago, IL*: American Association of Diabetes Educators*: 67– 96

Saarni C. (1999). *The development of emotional competence*. New York: Guilford Press ed.

Saccoa WP, Morrisonb AD, Malone JI. (2004). A brief, regular, proactive telephone ‘‘coaching’’ intervention for diabetes Rationale, description, and preliminary results. *Journal of Diabetes and Its Complications* 18; 113–118

Sarafino Ep. (2002). *Health Psychology*. 4^th^ ed. New York: John Wiley & Sons, Inc.

Sarason IG. (1988). Social support, personality and health. In M. Janniss (Ed.). Individual differences, stress and health psychology. Oxford, England: *John Wiley & Sons. Topics in health psychology*: 245-256

Schafer LC, Mccaul KD, Glasgow RE. (1986). Supportive and Nonsupportive Family Behavior: Relationship to Adherence and Metabolic control in Person with type 1 diabetes. *Diabetes Care*. 9:179-185.

Schreurs KM, Ridder DT de. (1997). Integration of coping and social support perspectives: implications for the study of adaptation to chronic diseases. *Clin Psychol Rev*; 17: 89– 112.

Seibel JA. (2010). Coping with Chronic Illness. American Diabetes Association. [Cited: July 06, 2010], Available from: URL: [*http://diabetes.webmd.com/guide/stress-management*](http://diabetes.webmd.com/guide/stress-management)

Sridhar GR, Madhu K. (2002). Psychosocial and cultural issues in diabetes mellitus. Current Science; 83: 1556-64.

Shapiro AM, Ricordi C, Hering BJ. Auchincloss H, Lindbalad R, Robertson RP, Secchi A, Brendel MD, Berney T, Brennan DC, Cagliero E, Alejandro R, Ryan EA, DiMercurio B, Morel P, Polonsky KS, Reems JA, Bretzel RG, Bertuzzi F, Froud T, Kandaswamy R, Sutherland DE, Eisenbarth G, Segal M, Preiksaitis J, Korbutt GS, Barton FB, Viviano L, Seyfert-Margolis V, Bluestone J, Lakey JR. (2006). International trial of the Edmonton protocol for islet transplantation. *N Engl J Med*; 355: 1318-30.

Shaw BA, Gallant MP, Jacome MR, Spokane LS. (2006). Assessing sources of support for diabetes self- care in urban and rural underserved communities*. J Community Health*. 31(5):393-408.

Siebolds M, Gaedeke O, Schwedes U. (2006). Self-monitoring of blood glucose-Psychological aspects relevant to changes in HbA1c in type 2 diabetic patients treated with diet or diet plus oral antidiabetic. *Patient Education and Counseling*, 62:104–11

Skinner TC, Carey ME, Cradock S, Daly H, Davies MJ, Doherty Y, Heller S, Khunti K and Oliver L. (2006). Diabetes education and self-management for ongoing and newly diagnosed (DESMOND): Process modelling of pilot study .*Patient Education and Counseling* ,64: 369–377

Snoek FJ. (2005). Psychology in Diabetes Care. England; West Sussex: *John Wiley & Sons Ltd*. 2 nd ed.P: 67

Snoek FJ, Skinner TC. (2006). Psychological aspects of diabetes management. *Medicine*; 34: 61-62.

Snoek FJ. and Skinner TC. (2002).Psychological councelling in problematic diabetes: does it help?. *Diabet Med*. 19: 265-273.

Snoek FJ, Van der Ven NCW, lubach C, Chatrou M, Ader HJ, Heine RJ and Jacobson AM. (2001). Effect of cognitive behavioural group training (CBGT) in adult patients with poorly controlled insulin-dependent (type1) diabetes: a pilot study. *Patient Education and Counselling*. 45:143-148.

Snoek FJ, Van der Ven NCW and lubach C. (1999). Cognitive behavioural group training (CBGT) for poorly controlled type1 diabetes patients: a psychoeducational approach. *Diabetes Spectrum*. 12:147-52.

Sommerfield AJ, Deary IJ, Frier BM. (2004). Acute hyperglycemia alters mood state and impairs cognitive performance in people with type 2 diabetes. *Diabetes Care*; 27: 2335–2340.

Speight J, Reaney MD & Barnard KD. (2009). Not all roads lead to Rome—a review of quality of life measurement in adults with diabetes. *Diabetic Medicine*, 26(4), 315–327.

Sridhar GR, Madhu K. (2002). Psychosocial and cultural issues in diabetes mellitus. *Current Science;* 83: 1556-64.

Steinmetz A. (2003). *Treatment of diabetic dislipoproteinemia*. *Exp Clin Endol Diabetes*; 111:239-45.

Stenstrom U, Goth A, Carlsson C, Andersson PO. (2003). Stress management training as related to glycemic control and mood in adults with Type 1 diabetes mellitus. *Diabetes Research and Clinical Practice,* 60: 147_/152

Surwit RS, VanTilburg MA, Zucker N, McCaskill CC, Parekh P, Feinglos, MN, Edwards CL, Williams P and Lane JD. (2002). Stress management improves long-term glycemic control in type 2 diabetes. *Diabetes Care*; 25: 30-34.

Taylor, S. E. (2003). *Health psychology* (5^th^ ed.). New York: McGraw-Hill.

Thompson RA, Meyer S. ( 2007). Socialization of emotion regulation in the family. In: Gross JJ, editor. Handbook of emotion regulation. New York: *Guilford Press ed*. p. 249–68.

The Report of the Expert Committee on the Diagnosis and Classification of Diabetes Mellitus. (2003). *Diabetes Care*. 26,1: S5 –S20

Thrailkill KM, Clay Bunn R, Fowlkes JL. (2009). Matrix metalloproteinases: their potential role in the pathogenesis of diabetic nephropathy.*Endocrine*, 35: 1-10.

Twohig, M. P., Hayes, S. C., & Masuda, A. (2006a). Increasing willingness to experience obsessions: Acceptance and commitment therapy as a treatment for obsessive-compulsive disorder. *Behavior Therapy*, 37, 3-13.

Toobert D J, Hampson SE, & Glasgow RE. (2000). The Summary of Diabetes Self-Care Activities Measure: Results from 7 studies and a revised scale. *Diabetes Care*, 23, 943-950.

Tooke JE, Goh KL. (1999).*Vascular function in type 2 diabetes mellitus and pre-diabetes: the case for intrinsic endotheiopathy*. *Diabet Med*; 16:710–5.

Tuncay T, Musabak I, Engin Gok D, Kutlu M. (2008). The relationship between anxiety, coping strategies and characteristics of patients with diabetes. *Health and Quality of Life Outcomes*; 6(79): 1-9.

Van der Ven NC, Lubach CH, Hogenelst MH, van Iperen A, Tromp-Wever AM, Vriend A, Van der Ploeg HM, Heine RJ and Snoeck FJ. (2005). Cognitive behavioural group training (CBGT) for patients with type 1 diabetes in persistent poor glycaemic control: who do we reach?. *Patient Educ Couns*; 56: 313-22.

Van der Ven NC, Hogenelst MH, Tromp-Wever AM, Twisk JW, van der Ploeg HM, Heine RJ and Snoek FJ. (2005). Short-term effects of cognitive behavioural group training (CBGT) in adult Type 1 diabetes patients in prolonged poor glycaemic control.A randomized controlled trial. *Diabet Med*.22:1619–23.

Vlaeyen JWS & Morley S. (2005). Cognitive-behavioral treatments for chronic pain: What works for whom? *Clinical Journal of Pain*. 21(1): 1-8.

Vlaeyen J, Crombez G, Goubert L. (2007). The psychology of chronic pain and its management*. Phys Ther Rev.* 12: 179-88.

Vowles KE & Thompson M. (2011). Acceptance and Commitment Therapy for chronic pain. In L. M. McCracken (Ed.) Mindfulness and Acceptance in Behavioral Medicine: Current *Theory and Practice*: 31-60.

Wang HH, Wu SZ and Liu YY. (2003). Association between social support and health outcomes: A meta-analysis. *Kaohsiung journal of medical science*, 19: 345-351.

Wang HF and Yeh MC. (2012). The quality of life of adults with type 2 diabetes in a hospital care clinic in Taiwan. *Quality of Life Research.* [Springer](http://dx.doi.org/10.1007/s11136-012-0178-7): DOI: 10.1007/s11136-012-0178-7

Watkin PJ. (2003). ABC of diabetes. London: *BMJ group*: 47-50.

Wen LK, Shepherd MD, Parchman ML. (2004). Family support, diet, exercise among older Mexican American with type 2 diabetes. *Diabetes Edu*. 30(6):980-993.

Wild SH, Roglic G, Sicree R, Green A, King H. (2000). Global burden diabetes mellitus in the year. *Global Burden of Disease*: 1-28

Winkley K, Landau S, Eisler I, Ismail K. (2006). Psychological interventions to improve glycaemic control in patients with type 1 diabetes: systematic review and meta-analysis of randomised controlled trials. Cite this article as: *BMJ, doi:10.1136/bmj.38874.652569.55*.27:1-5

Yadav R, Tiwari P and Dhanaraj E. (2008). Risk factors and complications of type 2 diabetes in Asians. *CRIPS*. 9. 2: 8-12

Yoo JS, Hwan AR, Lee HC, Kim CJ. (2003). Development and validation of a computerized exercise intervention program for patients with type 2 diabetes mellitus in Korea. *Younsei Medical Journal*. 44(5):892-904

Zimmet PZ. (1999). Diabetes epidemiology as a tool to trigger diabetes research and care. *Diabetologia*. 42: 499-518

Zimet GD, Dehlam, NW, Farley GK, Werkan S and Berkoff k. (1988). The Multidimensional Scale of Perceived Social Support. *Journal of Personality Assessment*, 52, 30-41.

1. جدول متغيرها (Variables) و تعريف واژه ها (Definition of Terms) :

| رديف | عنوان متغير | نوع متغير | | كمي | | كيفي | | تعريف علمي - عملي | نحوه اندازه گيري | مقياس |
| --- | --- | --- | --- | --- | --- | --- | --- | --- | --- | --- |
|  |  | مستقل | وابسته | پيوسته | گسسته | اسمي | رتبه‏اي |  |  |  |
| 1 | خودمراقبتی |  | ◼ | ◼ |  |  |  | تعریف علمی: خود مراقبتی اقدامات و فعالیت های آگاهانه ،آموخته شده و هدفداری است که توسط فرد به منظور حفظ حیات و تأمین حفظ و ارتقای سلامت خود و خانواده انجام می شود.  تعریف عملیاتی: در پژوهش حاضر خودمراقبتی با استفاده از مقیاس خودمراقبتی توبرت و همکاران (2000) سنجیده می­شود. | پرسشنامه | مقیاس خود مراقبتی |
| 2 | درمان پذیرش و تعهد | ◼ |  | ◼ |  |  |  | تعریف علمی: درمان پذیرش و تعهد یک مداخله­ی روانشناختی مبتنی بر تجربه است که راهبردهای مبتنی بر آگاهی و پذیرش را همراه با راهبردهای تعهد و تغییر رفتار به منظور افزایش انعطاف پذیری روانشناختی به کار می­گیرد که هستۀ اصلی آن انعطاف پذیری روانشناختی است، به این معنی است که فرد به طور کامل با لحظات کنونی تماس داشته و بر اساس مقتضیات وضعیتی که در آن قرار دارد رفتار خود را در راستای ارزشهایی که برگزیده است تغییر یا ادامه دهد  تعریف عملیاتی: در پژوهش حاضر پروتکل گریگ و همکاران (2007) برای درمان پذیرش و تعهد استفاده خواهد شد. | پرسشنامه | نتیجه مداخله به همراه مقیاس AADQ |
|  | نمايه توده­ي بدني |  | ◼ | ◼ |  |  |  | تعریف علمی: شاخص حجم تودۀ بدنی از طریق اندازه­گیری قد و وزن فرد، محاسبه می­گردد. این شاخص در واقع وزن متناسب با قد را می­سنجد و می­توان به عنوان مناسب­ترین شاخص در حیطه­ی وزن از آن استفاده کرد. شاخص تودۀ بدنی از تقسیم وزن به کیلوگرم بر مجذور قد به متر محاسبه می­شود  تعریف عملیاتی: در اینجا نیز از طریق فرمول بالا محاسبه می­گردد. | وزن/قد^2^ | کيلوگرم بر مترمربع |
|  | هموگلوبین گلیکوزیله |  | ◼ | ◼ |  |  |  | تعریف علمی: **آزمایش خونی است که نشانگر میانگین قند خون فرد در طی 2 تا 3 ماه گذشته است. نتایج این آزمایش نشان می دهد که چند درصد از هموگلوبین خون با قند ترکیب شده است، که هر چه این درصد بالاتر باشد، نشانگر بالا بودن متوسط میزان قند خون است.** | نمونه گیری خون | mg/dl |

نام ونام خانوادگي

امضاي مجري يا مجريان طرح

دکتر پریسا امیری

دکتر زینب شایقیان

## فــرم اخــلاق در پـــژوهش

**عنوان تحقيق:** نقش تعدیل­کننده سبکهای مقابله­ و حمایت اجتماعی در تأثیر درمان گروهی پذیرش و تعهد بر هموگلوبین گلیکوزیله، کیفیت زندگی و خودمراقبتی در بیماران مبتلا به دیابت نوع II

**بله خير**

1. آيا اجراي طرح مغايرتي با اعتقادات باورها و سنتهاي جامعه دارد؟ □ ■
2. در طرح پيشنهادي ، پرسشنامه ها و يا فرمهاي اطلاعاتي، مطالب موهن و زنده بكار برده شده است؟ □ ■
3. در بازنگري منابع و استفاده از مقالاتي كه در فهرست منابع ذكر گرديده رعايت صداقت وامانت شده است؟ ■ □
4. ‌آيا در طراحي پروژه از منابع مشكوك و فاقد اعتبار استفاده گرديده است؟ □ ■
5. آيا از مناسبترين روش تحقيق و جديدترين تكنيك هاي ممكن استفاده شده است؟ ■ □
6. از مواد، ابزار و روشهاي تهاجمي كه موجب آسيب جسمي يا روحي شود استفاده مي گردد؟ □ ■
7. آزادي فردي داوطلبين با بيماران رعايت شده است؟ ■ □
8. در رضايت نامه تنظيم شده رعايت صداقت بعمل آمده و آيا به امضاي افراد خواهد رسيد؟ ( يك نسخه از آن ضميمه گردد ■ □
9. آيا پذيرش مسئوليت جبران خسارات اجتماعي فوق الذكر در فرم رضايت نامه درج گرديده است؟ ■ □
10. تجويز دارو، دارو نما و يا مداخله از نظر اخلاقي اشكالي دارد؟ □ ■
11. آيا استفاده از دارونما به اطلاع بيمار خواهد رسيد؟ ■ □
12. حقوق افراد صغير ويا كسانيكه قيم لازم دارند حفظ شده است؟ ■ □
13. آيا خساراتيكه ممكن است سهواً به افراد مورد بررسي وارد گردد جبران خواهد شد؟ ■ □
14. آيا هزينه هاي تحصيلي در طرح به افراد مورد بررسي پرداخت خواهد شد؟ ■ □
15. آيا تمامي اطلاعات مربوط به افراد مورد بررسي بطور محرمانه ضبط وباقي خواهد ماند؟ ■ □

****************************************************************

**نظريه نهايي و توصيه‌هاي شوراي پژوهشي:**

# نظر كميته اخلاق پژوهشكده در مورد رعايت اصول اخلاقي در پژوهش

**مثبت ■ منفي□**

**اين فرم بايستي توسط پژوهشگر اصلي تكميل و همراه با پروژه حقيقي پيشنهادي به شوراي پژوهشي پژوهشكده ارائه شده است.**

پيوست شماره دو

1. Retinopathy [↑](#footnote-ref-1)
2. Neuropathy [↑](#footnote-ref-2)
3. Nephropathy [↑](#footnote-ref-3)
4. شرح کامل عوارض دیابت و تقسیم بندی آن در پیوست 2 آمده است. [↑](#footnote-ref-4)
5. Impaired glucose tolerance [↑](#footnote-ref-5)
6. Hyperglycemia [↑](#footnote-ref-6)
7. Myocardial Infarction [↑](#footnote-ref-7)
8. Coronary Heart Disease [↑](#footnote-ref-8)
9. Peripheral vessels disease [↑](#footnote-ref-9)
10. Central vessels diesease [↑](#footnote-ref-10)
11. Arterioscleroses [↑](#footnote-ref-11)
12. Syndrome X [↑](#footnote-ref-12)
13. Self-Care [↑](#footnote-ref-13)
14. Peyrot M. [↑](#footnote-ref-14)
15. Lin EH [↑](#footnote-ref-15)
16. Sommerfield [↑](#footnote-ref-16)
17. Cox D [↑](#footnote-ref-17)
18. Hyperglycemic stimulus [↑](#footnote-ref-18)
19. Avoidance [↑](#footnote-ref-19)
20. Denial [↑](#footnote-ref-20)
21. Myocardial Infarction [↑](#footnote-ref-21)
22. Liu [↑](#footnote-ref-22)
23. Stress management [↑](#footnote-ref-23)
24. Relaxation [↑](#footnote-ref-24)
25. Cognitve Behavioral Treatment [↑](#footnote-ref-25)
26. Self Management [↑](#footnote-ref-26)
27. unlearned [↑](#footnote-ref-27)
28. Peyrot M. [↑](#footnote-ref-28)
29. Self-defeating [↑](#footnote-ref-29)
30. Negatve behaviors [↑](#footnote-ref-30)
31. distress [↑](#footnote-ref-31)
32. Self-efficacy [↑](#footnote-ref-32)
33. Acceptance and commitment therapy [↑](#footnote-ref-33)
34. treatments mindfulness- and acceptance-based [↑](#footnote-ref-34)
35. Mindfulness-Based Cognitive Therapy [↑](#footnote-ref-35)
36. Mindfulness-Based Cognitive Therapy [↑](#footnote-ref-36)
37. Dialectical Behavior Therapy [↑](#footnote-ref-37)
38. Third wave [↑](#footnote-ref-38)
39. Contextualism [↑](#footnote-ref-39)
40. Relational Frame Theory [↑](#footnote-ref-40)
41. Contextual behavioral science approach [↑](#footnote-ref-41)
42. Self-defeating [↑](#footnote-ref-42)
43. Negatibe behaviors [↑](#footnote-ref-43)
44. Experiential avoidance [↑](#footnote-ref-44)
45. Behavioral Suppression [↑](#footnote-ref-45)
46. Acceptance [↑](#footnote-ref-46)
47. Defusion [↑](#footnote-ref-47)
48. Present moment [↑](#footnote-ref-48)
49. Self as a context [↑](#footnote-ref-49)
50. Values [↑](#footnote-ref-50)
51. Committed action [↑](#footnote-ref-51)
52. Psychological flexibility [↑](#footnote-ref-52)
53. Psychological acceptance [↑](#footnote-ref-53)
54. Psychological awareness [↑](#footnote-ref-54)
55. Cognitive defusion [↑](#footnote-ref-55)
56. Committed action [↑](#footnote-ref-56)
57. Basa RP [↑](#footnote-ref-57)
